# Risk prediction models for selection of lung cancer screening candidates: A retrospective validation study

**DOI:** 10.1371/journal.pmed.1002277

**Published:** 2017-04-04

**Authors:** Kevin ten Haaf, Jihyoun Jeon, Martin C. Tammemägi, Summer S. Han, Chung Yin Kong, Sylvia K. Plevritis, Eric J. Feuer, Harry J. de Koning, Ewout W. Steyerberg, Rafael Meza

**Affiliations:** 1 Department of Public Health, Erasmus MC University Medical Center Rotterdam, Rotterdam, the Netherlands; 2 Department of Epidemiology, University of Michigan, Ann Arbor, Michigan, United States of America; 3 Department of Health Sciences, Brock University, St. Catharines, Ontario, Canada; 4 Department of Radiology, Stanford University, Palo Alto, California, United States of America; 5 Department of Medicine, Stanford University, Palo Alto, California, United States of America; 6 Department of Radiology, Massachusetts General Hospital, Boston, Massachusetts, United States of America; 7 Division of Cancer Prevention, National Cancer Institute, Bethesda, Maryland, United States of America; University of Texas Southwestern Medical Center at Dallas, UNITED STATES

## Abstract

**Background:**

Selection of candidates for lung cancer screening based on individual risk has been proposed as an alternative to criteria based on age and cumulative smoking exposure (pack-years). Nine previously established risk models were assessed for their ability to identify those most likely to develop or die from lung cancer. All models considered age and various aspects of smoking exposure (smoking status, smoking duration, cigarettes per day, pack-years smoked, time since smoking cessation) as risk predictors. In addition, some models considered factors such as gender, race, ethnicity, education, body mass index, chronic obstructive pulmonary disease, emphysema, personal history of cancer, personal history of pneumonia, and family history of lung cancer.

**Methods and findings:**

Retrospective analyses were performed on 53,452 National Lung Screening Trial (NLST) participants (1,925 lung cancer cases and 884 lung cancer deaths) and 80,672 Prostate, Lung, Colorectal and Ovarian Cancer Screening Trial (PLCO) ever-smoking participants (1,463 lung cancer cases and 915 lung cancer deaths). Six-year lung cancer incidence and mortality risk predictions were assessed for (1) calibration (graphically) by comparing the agreement between the predicted and the observed risks, (2) discrimination (area under the receiver operating characteristic curve [AUC]) between individuals with and without lung cancer (death), and (3) clinical usefulness (net benefit in decision curve analysis) by identifying risk thresholds at which applying risk-based eligibility would improve lung cancer screening efficacy. To further assess performance, risk model sensitivities and specificities in the PLCO were compared to those based on the NLST eligibility criteria. Calibration was satisfactory, but discrimination ranged widely (AUCs from 0.61 to 0.81). The models outperformed the NLST eligibility criteria over a substantial range of risk thresholds in decision curve analysis, with a higher sensitivity for all models and a slightly higher specificity for some models. The PLCOm2012, Bach, and Two-Stage Clonal Expansion incidence models had the best overall performance, with AUCs >0.68 in the NLST and >0.77 in the PLCO. These three models had the highest sensitivity and specificity for predicting 6-y lung cancer incidence in the PLCO chest radiography arm, with sensitivities >79.8% and specificities >62.3%. In contrast, the NLST eligibility criteria yielded a sensitivity of 71.4% and a specificity of 62.2%. Limitations of this study include the lack of identification of optimal risk thresholds, as this requires additional information on the long-term benefits (e.g., life-years gained and mortality reduction) and harms (e.g., overdiagnosis) of risk-based screening strategies using these models. In addition, information on some predictor variables included in the risk prediction models was not available.

**Conclusions:**

Selection of individuals for lung cancer screening using individual risk is superior to selection criteria based on age and pack-years alone. The benefits, harms, and feasibility of implementing lung cancer screening policies based on risk prediction models should be assessed and compared with those of current recommendations.

## Abstract 2

Kevin ten Haaf and colleagues present a new prediction model for candidate screening for lung cancer. The new model considers an individual's risk rather than age and pack years smoked.

## Author summary

**Why was this study done?:**

- In the United States, lung cancer screening is currently recommended based on age, pack-years smoked, and years since smoking cessation, the criteria used to select participants for the National Lung Screening Trial (NLST).
- A number of recent investigations suggest that using lung cancer risk prediction models could lead to more effective screening programs compared to the current recommendations.
- External validation and direct comparisons between risk models are often limited due to insufficient numbers of events or methodological limitations.

**What did the researchers do and find?:**

- Various performance characteristics of nine risk prediction models for lung cancer incidence or mortality were assessed using data from two randomized controlled trials on lung cancer screening: the NLST and the Prostate, Lung, Colorectal and Ovarian Cancer Screening Trial (PLCO).
- The calibration performance of the models was satisfactory, but discrimination ranged widely between models. However, all models had a higher sensitivity—and some models had a slightly higher specificity—than the NLST eligibility criteria.

**What do these findings mean?:**

- Using risk prediction models to select individuals for lung cancer screening is superior to currently recommended selection criteria.
- The benefits, harms, and feasibility of using risk prediction models to select individuals for lung cancer screening should be assessed and compared with current recommendations.

## Introduction

The National Lung Screening Trial (NLST) found that screening with low-dose computed tomography (CT) can reduce lung cancer mortality by 20% [1]. Based on an evidence review, including the results of the NLST and a comparative microsimulation modeling study, the United States Preventive Services Task Force (USPSTF) recommended lung cancer screening for current and former smokers aged 55 through 80 y who smoked at least 30 pack-years and, if quit, quit less than 15 y ago [2–4]. To our knowledge, only the United States has implemented lung cancer screening policies. Although the province of Ontario, Canada, recommends screening individuals at high risk for lung cancer through an organized program, no program has yet been established [5]. Cancer Care Ontario (the provincial cancer agency of Ontario) is currently evaluating the feasibility of implementing such a program [6]. European countries have not yet made any recommendations on lung cancer screening, as the final results of the Dutch-Belgian Lung Cancer Screening Trial (Nederlands-Leuvens Longkanker Screenings Onderzoek [NELSON] trial), potentially pooled with high-quality data from other trials, are still awaited [7–9].

The screening eligibility criteria used in the current USPSTF recommendations are based on age and pack-years, a measure of cumulative smoking exposure. Thus, these recommendations do not take other important risk factors into account, such as family history, nor other relevant aspects of smoking, such as smoking duration or intensity. Recently, a number of investigations have suggested that determining screening eligibility using an individual’s risk based on age, more detailed smoking history, and other risk factors such as ethnicity and family history of lung cancer could lead to more effective screening programs compared with the USPSTF recommendations [10–13]. Indeed, some lung cancer screening guidelines already encourage assessment of an individual’s risk to determine screening eligibility [14].

While various lung cancer risk prediction models have been developed, external validation and direct comparisons between models have been limited due to insufficient numbers of events or methodological limitations [15–21]. Such validations are essential, as risk prediction models generally have optimistic performance within their development dataset [15–17]. This study aims to externally validate and directly compare the performance of nine currently available lung cancer risk prediction models for stratifying lung cancer risk groups and determining screening eligibility.

## Methods

### Ethics statement

No identifiable information was used; therefore, no institutional review board (IRB) approval was needed. Nonetheless, a determination of exempt was given by the University of Michigan IRB (HUM00054750), and a determination of this not being human subjects research was given by the Fred Hutchinson Cancer Research Center (former affiliation of J. J.) IRB (6007–680).

### Study population

We used data from two large randomized controlled screening trials: the NLST and the Prostate, Lung, Colorectal and Ovarian Cancer Screening Trial (PLCO) [1,22–24]. All participants in the CT arm (*n =* 26,722) and chest radiography (CXR) arm (*n =* 26,730) of the NLST and ever-smoking participants in the CXR arm (*n =* 40,600) and control arm (*n =* 40,072) of the PLCO were included in the analysis. Never-smokers in the PLCO were not considered, as (1) not all lung cancer risk prediction models can be applied to never-smokers and (2) never-smokers are unlikely to reach levels of risk that allow them to benefit from screening [13,25].

Data on the predictor variables in each trial were collected through epidemiologic questionnaires administered at study entry and harmonized across both trials. Reported average numbers of cigarettes smoked per day above 100 were considered implausible and recoded as 100 cigarettes per day (*n =* 11). Furthermore, body mass index values less than 14 and over 60 kg/m^2^ were considered implausible for enrollment in both trials and recoded as 14 (*n =* 5) and 60 kg/m^2^ (*n =* 18), respectively. Lung cancer diagnoses (1,925 in the NLST and 1,463 in the PLCO) and lung cancer deaths (884 in the NLST and 915 in the PLCO) that occurred between study entry and 6 y of follow-up were included in the final dataset and were considered as binary outcomes.

### Lung cancer risk prediction models

Our study includes nine risk prediction models for lung cancer incidence or death that have been used frequently in the literature. Risk prediction models were not considered for this investigation, if they (1) were developed for specific ethnicities and are therefore not broadly applicable [26–28], (2) used information on biomarkers or lung nodules and are therefore not readily applicable for the prescreening selection of individuals [29–33], (3) were developed for identifying symptomatic patients [34,35], (4) did not incorporate smoking behavior [36], (5) did not provide information on parameter estimates (e.g., baseline risk parameters) necessary to allow replication of the model [11,12], or (6) had poor discriminative ability in their development dataset [37].

Nine models remained and were investigated: the Bach model, the Liverpool Lung Project (LLP) model, the PLCOm2012 model, the Two-Stage Clonal Expansion (TSCE) model for lung cancer incidence, the Knoke model, two versions of the TSCE model for lung cancer death [10,38–44], and simplified versions of the PLCOm2012 and LLP models. The characteristics of these models are shown in Table 1. The TSCE and Knoke models consider only age, gender, and smoking-related characteristics as risk factors [40–43]. The Bach model considers asbestos exposure as an additional risk factor, while the LLP and PLCOm2012 models consider multiple additional risk factors [10,38,39]. The simplified versions of the PLCOm2012 and LLP models considered only age, gender, and smoking variables. A detailed description of each model can be found in S1 Appendix.

**Table 1:** Characteristics of investigated risk models.

Model	Predicted outcome	Model prediction time frame	Development dataset(s)	Risk factors incorporated in model	Reference
Bach model*	Lung cancer incidence	1 y (iterative)	Carotene and Retinol Efficacy Trial (CARET)	Age, gender, smoking duration, smoking intensity, years since cessation, asbestos exposure	[39]
Liverpool Lung Project (LLP) model^†^	Lung cancer incidence	5 y	Liverpool Lung Project (LLP) case–control study	Age, gender, smoking duration, personal history of cancer, family history of lung cancer, personal history of pneumonia, asbestos exposure	[44]
PLCOm2012 model^†^	Lung cancer incidence	6 y	Prostate, Lung, Colorectal and Ovarian Cancer Screening Trial (PLCO)	Age, race, education, BMI, COPD, personal history of cancer, family history of lung cancer, smoking status, smoking duration, smoking intensity, years since cessation	[10]
Two-Stage Clonal Expansion (TSCE) lung cancer incidence model	Lung cancer incidence	1 y (iterative)	Nurses’ Health Study (NHS), Health Professionals Follow-up Study (HPFS)	Age, gender, smoking status, smoking duration, smoking intensity, years since cessation	[43]
Knoke model	Lung cancer death	1 y (iterative)	American Cancer Society’s first Cancer Prevention Study (CPS-I)	Age, smoking status, smoking duration, smoking intensity, years since cessation	[40]
Two-Stage Clonal Expansion (TSCE) CPS lung cancer death model	Lung cancer death	1 y (iterative)	British Doctors Study, American Cancer Society’s first Cancer Prevention Study (CPS-I), American Cancer Society’s second Cancer Prevention Study (CPS-II)	Age, gender, smoking status, smoking duration, smoking intensity, years since cessation	[41]
Two-Stage Clonal Expansion (TSCE) NHS/HPFS lung cancer death model	Lung cancer death	1 y (iterative)	Nurses’ Health Study (NHS), Health Professionals Follow-up Study (HPFS)	Age, gender, smoking status, smoking duration, smoking intensity, years since cessation	[42]

*Data on asbestos exposure was not available for PLCO participants and could not be accurately derived for NLST participants. Therefore, only age, gender, and smoking-related characteristics were considered for this model.

^†^Simplified versions of these models, using only age, gender, and smoking-related characteristics, were considered as well.

BMI, body mass index; COPD, chronic obstructive pulmonary disease.

Data on frequency and intensity of asbestos exposure, used in the LLP and Bach models, was not available for the PLCO participants and could not be accurately derived for the NLST participants [38,39]. Therefore, we assumed that none of the participants were exposed to asbestos, even though this assumption may lead to biased estimates [45]. However, as the potential number of individuals with asbestos exposure was low (less than 5% of the NLST participants reported ever working with asbestos), this bias is expected to be minor [46].

The LLP model incorporates age at lung cancer diagnosis of a first-degree relative: early age (60 y or younger) versus late age (older than 60 y) [38]. However, while both the PLCO and the NLST had information about the occurrence of family history of lung cancer (yes/no), neither had information on the age of diagnosis for the affected relative(s). Since the median age of lung cancer diagnosis in the United States is 70 y and the majority of lung cancers occur after the age of 65 y (68.6%), we assumed that lung cancer in first-degree relatives in the PLCO and the NLST always occurred after the age of 60 y [47,48].

In addition, the LLP model incorporates a history of pneumonia as a risk factor [38]. While information on this risk factor was available in the NLST, it was not available in the PLCO. Therefore, we assumed that none of the PLCO participants had a history of pneumonia for the complete case analyses. While 22.1% of NLST participants had a history of pneumonia (Table 2), the association of a history of pneumonia with a lung cancer diagnosis within 6 y was not clear (*p* = 0.3378 in the CT arm and *p* = 0.0035 in the CXR arm). Missing history of pneumonia for PLCO participants was imputed by using information from the NLST participants [49].

**Table 2:** Baseline characteristics of National Lung Screening Trial and Prostate, Lung, Colorectal and Ovarian Cancer Screening Trial participants according to 6-y lung cancer incidence.

Characteristic	NLST computed tomography arm			NLST chest radiography arm			PLCO chest radiography arm			PLCO control arm		
	No lung cancer	Lung cancer	*p*-Value	No lung cancer	Lung cancer	*p*-Value	No lung cancer	Lung cancer	*p*-Value	No lung cancer	Lung cancer	*p*-Value
**Number (percent) of participants**	25,692 (96.15%)	1,030 (3.85%)		25,835 (96.65%)	895 (3.35%)		39,846 (98.14%)	754 (1.86%)		39,363 (98.23%)	709 (1.77%)	
**Age (years)**												
Median (IQR)	60 (57–65)	63 (59–68)	<0.001	60 (57–65)	64 (60–68)	<0.001	62 (58–66)	65 (60.25–69)	<0.001	62 (58–66)	65 (60–69)	<0.001
Missing	0 (0%)	0 (0%)		0 (0%)	0 (0%)		0 (0%)	0 (0%)		0 (0%)	0 (0%)	
**Gender**												
Male	15,148 (58.96%)	621 (60.29%)	0.401	15,235 (58.97%)	526 (58.77%)	0.917	23,228 (58.29%)	475 (63.00%)	0.010	22,775 (57.86%)	438 (61.78%)	0.038
Female	10,544 (41.04%)	409 (39.71%)		10,600 (41.03%)	369 (41.23%)		16,618 (41.71%)	279 (37.00%)		16,588 (42.14%)	271 (38.22%)	
Missing	0 (0%)	0 (0%)		0 (0%)	0 (0%)		0 (0%)	0 (0%)		0 (0%)	0 (0%)	
**Hispanic ethnicity**												
No	25,070 (97.58%)	1,008 (97.86%)	0.041	25,158 (97.38%)	881 (98.44%)	0.004	38,116 (95.66%)	731 (96.95%)	0.314	37,597 (95.51%)	690 (97.32%)	0.093
Yes	469 (1.83%)	10 (0.97%)		451 (1.75%)	5 (0.56%)		872 (2.19%)	12 (1.59%)		873 (2.22%)	9 (1.27%)	
Missing	153 (0.60%)	12 (1.17%)		226 (0.87%)	9 (1.01%)		858 (2.15%)	11 (1.46%)		893 (2.27%)	10 (1.41%)	
**Race or ethnic group**												
White	23,019 (89.60%)	933 (90.58%)	0.323	23,143 (89.58%)	806 (90.06%)	0.090	35,180 (88.29%)	644 (85.41%)	<0.001	34,787 (88.37%)	630 (88.86%)	0.007
Black	1,140 (4.44%)	47 (4.56%)		1,123 (4.35%)	51 (5.70%)		2,252 (5.65%)	73 (9.68%)		2,201 (5.59%)	53 (7.48%)	
Hispanic	337 (1.31%)	7 (0.68%)		314 (1.22%)	4 (0.45%)		810 (2.03%)	12 (1.59%)		807 (2.05%)	8 (1.13%)	
Asian	541 (2.11%)	18 (1.75%)		522 (2.02%)	14 (1.56%)		1232 (3.09%)	16 (2.12%)		1199 (3.05%)	13 (1.83%)	
Native Hawaiian or Pacific Islander	88 (0.34%)	3 (0.29%)		100 (0.39%)	2 (0.22%)		219 (0.55%)	8 (1.06%)		244 (0.62%)	1 (0.14%)	
American Indian or Alaskan Native	86 (0.33%)	6 (0.58%)		95 (0.37%)	3 (0.34%)		130 (0.33%)	1 (0.13%)		105 (0.27%)	4 (0.56%)	
Missing	481 (1.87%)	16 (1.55%)		538 (2.08%)	15 (1.68%)		23 (0.06%)	0 (0%)		20 (0.05%)	0 (0%)	
**Education**												
Less than high school graduate	1,552 (6.04%)	89 (8.64%)	<0.001	1,525 (5.90%)	83 (9.27%)	<0.001	3,424 (8.59%)	123 (16.31%)	<0.001	3,418 (8.68%)	96 (13.54%)	<0.001
High school graduate	5,989 (23.31%)	284 (27.57%)		6,177 (23.91%)	261 (29.16%)		8,775 (22.20%)	199 (26.39%)		8,647 (21.97%)	197 (27.79%)	
Post-high-school training	3,587 (13.96%)	146 (14.17%)		3,562 (13.79%)	139 (15.53%)		5,341 (13.40%)	92 (12.20%		5,393 (13.70%)	98 (13.82%)	
Some college	5,957 (23.19%)	232 (22.52%)		5,893 (22.81%)	195 (21.79%)		9,269 (23.26%)	165 (21.88%)		9,159 (23.27%)	161 (22.71%)	
College graduate	4,357 (16.96%)	148 (14.37%)		4,342 (16.81%)	99 (11.06%)		6,626 (16.63%)	97 (12.86%)		6,385 (16.22%)	94 (13.26%)	
Postgraduate/professional	3,679 (14.32%)	101 (9.81%)		3,718 (14.39%)	102 (11.40%)		6,352 (15.94%)	77 (10.21%)		6,218 (15.80%	59 (8.32%0	
Missing	571 (2.22%)	30 (2.91%)		618 (2.39%)	16 (1.79%)		59 (0.15%)	1 (0.13%)		143 (0.36%)	4 (0.56%)	
**BMI (kg/m**^**2**^**)**												
Median (IQR)	27.32 (24.46–30.73)	26.38 (23.94–29.28)	<0.001	27.38 (24.50–30.63)	26.22 (23.62–29.25)	<0.001	26.68 (24.19–30.02)	26.16 (23.48–28.77)	<0.001	26.68 (24.18–29.90)	25.88 (23.45–28.81)	<0.001
Missing	146 (0.57%)	13 (1.26%)		206 (0.80%)	7 (0.78%)		494 (1.24%)	10 (1.33%)		742 (1.89%)	15 (2.12%)	
**COPD**												
No	21,283 (82.84%)	765 (74.27%)	<0.001	21,435 (82.97%)	643 (71.84%)	<0.001	36,381 (91.30%)	602 (79.84%)	<0.001	35,899 (91.20%)	567 (79.97%)	<0.001
Yes	4,409 (17.16%)	265 (25.73%)		4,400 (17.03%)	252 (28.16%)		3,465 (8.70%)	152 (20.16%)		3,464 (8.80%)	142 (20.03%)	
Missing	0 (0%)	0 (0%)		0 (0%)	0 (0%)		0 (0%)	0 (0%)		0 (0%)	0 (0%)	
**Emphysema**												
No	23,661 (92.09%)	878 (85.24%)	<0.001	23,734 (91.87%)	766 (85.59%)	<0.001	38,011 (95.39%)	655 (86.87%)	<0.001	37,404 (95.02%)	599 (84.49%)	<0.001
Yes	1,910 (7.43%)	146 (14.17%)		1,915 (7.41%)	122 (13.63%)		1,650 (4.14%)	96 (12.73%)		1,612 (4.10%)	99 (13.96%)	
Missing	121 (0.47%)	6 (0.58%)		186 (0.72%)	7 (0.78%)		185 (0.46%)	3 (0.40%)		347 (0.88%)	11 (1.55%)	
**Personal history of cancer**												
No	24,588 (95.70%)	956 (92.82%)	<0.001	24,554 (95.04%)	833 (93.07%)	0.007	38,033 (95.45%)	709 (94.03%)	0.078	37,532 (95.35%)	653 (92.10%)	<0.001
Yes	1,028 (4.00%)	68 (6.60%)		1,154 (4.47%)	58 (6.48%)		1,813 (4.55%)	45 (5.97%)		1,831 (4.65%)	56 (7.90%)	
Missing	76 (0.30%)	6 (0.58%)		127 (0.49%)	4 (0.45%)		0 (0%)	0 (0%)		0 (0%)	0 (0%)	
**Family history of lung cancer**												
No	19,741 (76.84%)	746 (72.43%)	0.004	19,812 (76.69%)	640 (71.51%)	0.001	33,718 (84.62%)	565 (74.93%)	<0.001	33,485 (85.07%)	541 (76.30%)	<0.001
Yes	5,554 (21.62%)	261 (25.34%)		5,570 (21.56%)	236 (26.37%)		4,514 (11.33%)	139 (18.44%)		4,414 (11.21%)	130 (18.34%)	
Missing	397 (1.55%)	23 (2.23%)		453 (1.75%)	19 (2.22%)		1614 (4.05%)	50 (6.63%)		1464 (3.72%)	38 (5.36%)	
**Personal history of pneumonia**												
No	19,905 (77.48%)	781 (75.83%)	0.338	20,023 (77.50%)	657 (73.41%)	0.004	Not measured in PLCO		—	Not measured in PLCO		—
Yes	5,690 (22.15%)	240 (23.30%)		5,646 (21.85%)	233 (26.03%)		Not measured in PLCO		—	Not measured in PLCO		—
Missing	97 (0.38%)	9 (0.87%)		166 (0.64%)	5 (0.56%)		Not measured in PLCO		—	Not measured in PLCO		—
**Smoking status**												
Current smoker	12,183 (47.42%)	601 (58.35%)	<0.001	12,274 (47.51%)	558 (62.35%)	<0.001	7,744 (19.43%)	332 (44.04%)	<0.001	7,655 (19.45%)	324 (45.70%)	<0.001
Former smoker	13,509 (52.58%)	429 (41.65%)		13,561 (52.49%)	337 (37.65%)		32,102 (80.57%)	422 (55.97%)		31,708 (80.55%)	385 (54.30%)	
Missing	0 (0%)	0 (0%)		0 (0%)	0 (0%)		0 (0%)	0 (0%)		0 (0%)	0 (0%)	
**Smoking duration (years)**												
Median (IQR)	40 (35–44)	44 (40–49)	<0.001	40 (35–44)	44 (40–49)	<0.001	28 (16–39)	42 (35–48)	<0.001	28 (16–39)	42 (35–47)	<0.001
Missing	0 (0%)	0 (0%)		0 (0%)	0 (0%)		766 (1.92%)	10 (1.33%)		877 (2.23%)	17 (2.40%)	
**Smoking intensity (cigarettes per day)**												
Median (IQR)	25 (20–35)	30 (20–40)	<0.001	25 (20–30.5)	25 (20–40)	0.029	20 (10–30)	30 (20–40)	<0.001	20 (10–30)	30 (20–40)	<0.001
Missing	0 (0%)	0 (0%)		0 (0%)	0 (0%)		78 (0.20%)	4 (0.53%)		112 (0.28%)	2 (0.28%)	
**Pack-years of smoking**												
Median (IQR)	48 (39–66)	57 (45–82)	<0.001	48 (39–66)	55.5 (44–78)	<0.001	28.5 (14–48)	51 (38–74)	<0.001	29 (14–49)	54 (40–75)	<0.001
Missing	0 (0%)	0 (0%)		0 (0%)	0 (0%)		827 (2.1%)	13 (1.7%)		957 (2.4%	19 (2.7%)	
**Years since cessation**												
Median (IQR)	7 (3–11)	5 (2–10)	<0.001	7 (3–11)	6 (2–11)	0.067	20 (10–30)	10 (4–19)	<0.001	20 (10–30)	10 (4–18.25)	<0.001
Missing	219 (0.9%)	5 (0.5%)		216 (0.8%)	8 (0.9%)		561 (1.4%)	5 (0.7%)		679 (1.7%)	5 (0.7%)	

Data are given as *n* (percent) or median (IQR).

BMI, body mass index; COPD, chronic obstructive pulmonary disease; IQR, interquartile range; NLST, National Lung Screening Trial; PLCO, Prostate, Lung, Colorectal and Ovarian Cancer Screening Trial.

### Statistical analyses

To assess the performance of the risk prediction models, several metrics were employed: calibration, discrimination, and clinical usefulness (net benefit over a range of risk thresholds) [50]. The performance of the investigated risk prediction models was assessed in each trial arm separately, for both lung cancer incidence and lung cancer mortality. We assessed both lung cancer incidence and mortality in both arms of both trials for all investigated risk models, as these outcomes may be influenced differently by screening. Screening may affect the predictive performance for lung cancer incidence, due to the advance in time of detection due to screening (lead time) and the detection of cancers that would never have been detected if screening had not occurred (overdiagnosis) [51–53]. Furthermore, CT screening reduces lung cancer mortality compared to CXR screening, which may influence the predictive performance of models for lung cancer mortality in the CT arm of the NLST [1]. Furthermore, the sensitivity and specificity of each model in the PLCO cohorts were compared to the sensitivity and specificity of the NLST/USPSTF smoking eligibility criteria (being a current or former smoker who smoked at least 30 pack-years and, if quit, quit less than 15 y ago). Model performance was assessed by varying follow-up duration and outcome (5- and 6-y lung cancer incidence or mortality) to investigate the effect of follow-up duration on the discrimination performance of each model [54]. The 5- and 6-y time frames were chosen because the LLP and PLCOm2012 models were calibrated to these respective time frames, and complete follow-up of NLST participants was limited to 6 y [10,38]. Since performance was similar for 5- and 6-y outcomes, only the results of the 6-y outcomes are presented. Performance was evaluated for the risk prediction models as presented in their original publication, without any recalibration or reparameterization to the NLST and the PLCO. The only exception is the PLCOm2012 model, which was originally developed based on data from the control arm of the PLCO [10]. All analyses were performed in R (version 3.3.0) [55].

### Aspects of calibration performance

Calibration plots were constructed for the observed proportions of outcome events against the predicted risks for individuals grouped by similar ranges of predicted risk [56]. Perfect predictions should show an ideal 45-degree line that can be described by an intercept of 0 and a slope of 1 in the calibration plot [57]. The calibration intercept quantifies the extent to which a model systematically under- or overestimates a person’s risk; an intercept value of 0 represents perfect calibration in the large. The calibration slope was estimated by logistic regression analysis, using the log odds of the predictions for the single predictor of the binary outcome [50]. For a (near-)perfect calibration in the large, a calibration slope less than 1 reflects that predictions for individuals with low risk are too low and predictions for individuals with high risk are too high [50]. The calibration plots, calibration in the large, and calibration slopes for each model were obtained using the R package rms [58].

### Discrimination

Discrimination reflects the capability of a model to distinguish individuals with the event from those without the event; the risk predicted by the model should be higher for individuals with the event compared with those without the event [59]. The area under the receiver operating characteristic curve (AUC) was used to assess discrimination, which ranges between 0.5 and 1.0 for sensible models. The AUCs for each model were obtained using the R package rms [58].

### Clinical usefulness

While discrimination and calibration are important statistical properties of a risk prediction model, they do not assess its clinical usefulness [50,54,59]. For example, if a false-negative result causes greater harm than a false-positive result, one would prefer a model with a higher sensitivity over a model that has a greater specificity but a slightly lower sensitivity, even though the latter might have a higher AUC [60].

In the context of selecting individuals for lung cancer screening, a model is clinically useful if applying that model to determine screening eligibility yields a better ratio of benefits to harms than not applying it. Decision curve analysis has been proposed to assess the net benefit of using a risk prediction model [60,61]. Decision curve analysis evaluates the net benefit of a model over a range of risk thresholds, i.e., the level of risk used to classify predictions as positive or negative for the predicted outcome. For example, for the PLCOm2012 model, a risk threshold of 1.51% has been suggested, meaning that individuals with an estimated risk of 1.51% or higher are classified as positive (and thus eligible for screening) and individuals with an estimated risk lower than 1.51% as negative (and thus ineligible for screening) [13].

The net benefit is defined as:

$$\text{net benefit}=\;\frac{\text{true positive count}-\left(\text{false positive count }*\text{ weighting factor}\right)}{\text{number of individuals assessed for screening eligibility}}$$

where the weighting factor is defined as:

$$\text{weighting factor}=\;\frac{\text{risk threshold}}{\left(1-\text{risk threshold}\right)}$$

This weighting factor represents how the relative harms of false-positive (classifying a person as eligible for screening who does not develop, or die from, lung cancer) and false-negative (classifying a person as ineligible for screening who develops, or dies from, lung cancer) results are valued at a given risk threshold, i.e., the ratio of harm to benefit, and is estimated by the threshold odds. For example, a risk threshold of 2.5% yields the following weighting factor:

$$\text{weighting factor}=\;\frac{0.025}{\left(1-0.025\right)}=\frac{1}{39}\;$$

This weighting factor implies that missing one case of lung cancer that could be detected through screening is valued as 39 times worse than unnecessarily screening one person, or that one case should be detected per 40 screened persons. Consequently, the less relative weight one gives to detecting a lung cancer case, the higher the risk threshold one will favor.

The net benefit can then be interpreted as follows: if the net benefit at a risk threshold of 2.5% is 0.002 greater compared with screening all persons eligible according to the NLST criteria, taking the weighing factor into account, this is equivalent to a net improvement in true-positive results of 0.002 × 1,000 = 2 per 1,000 persons assessed for screening eligibility, or a net reduction in false-positive results of 0.002 × 1,000/(0.025/0.975) = 78 per 1,000 persons assessed for screening eligibility [60]. Thus, if the risk model has a positive net benefit at the preferred risk threshold, this indicates that applying the model at this risk threshold provides a better ratio of benefits to harms than current screening guidelines based on pack-years. Decision curves visualize the net benefit over a range of risk thresholds, allowing one to discern whether and at which risk thresholds applying the risk model can be clinically useful [61]. Decision curves were used to determine at which range of risk thresholds applying the models provides a net benefit over using the NLST eligibility criteria for selecting individuals for lung cancer screening.

Finally, we identified the risk threshold for each model in the PLCO cohorts that selected a similar number of individuals for screening as the NLST eligibility criteria, on which most lung cancer screening recommendations are currently based. We then assessed the sensitivity (the number of individuals with lung cancer incidence or death classified as eligible for screening divided by the total number of individuals with lung cancer incidence or death) and specificity (the number of individuals without lung cancer incidence or death classified as ineligible for screening divided by the total number of individuals without lung cancer incidence or death) for each model compared to the NLST criteria at the chosen risk threshold, as reported before by Tammemägi et al. [13].

### Multiple imputation of missing values

Multiple imputation of missing data for all considered risk factors was performed through the method of chained equations using the R package MICE [62]. History of pneumonia was not measured in the PLCO but was measured in the NLST; therefore, data from the NLST were used to impute history of pneumonia for PLCO participants [49]. Analyses were performed using 20 imputations, and the results were pooled through applying Rubin’s rules [63]. The results of the analyses with imputation of missing variables were similar to those obtained from complete case analyses. The Transparent Reporting of a multivariable prediction model for Individual Prognosis Or Diagnosis (TRIPOD) guidelines suggest applying multiple imputation when missing data are present, as complete case analyses can lead to inefficient estimates [64,65]. Therefore, all analyses reported here were performed with multiple imputation of missing values.

## Results

### Characteristics of study populations

An overview of the characteristics of the four study cohorts (two trial arms in each trial) is given in Table 2, stratified by 6-y lung cancer incidence. A similar table stratifying participants by 6-y lung cancer mortality is provided in S2 Appendix. An overview of the proportion of individuals with complete information on all risk factors, stratified by trial arm and 6-y outcome, is given in S3 Appendix. Overall, approximately 93% of the study population had complete information for all considered risk factors.

### Differences in levels of absolute risk

**Fig 1:**
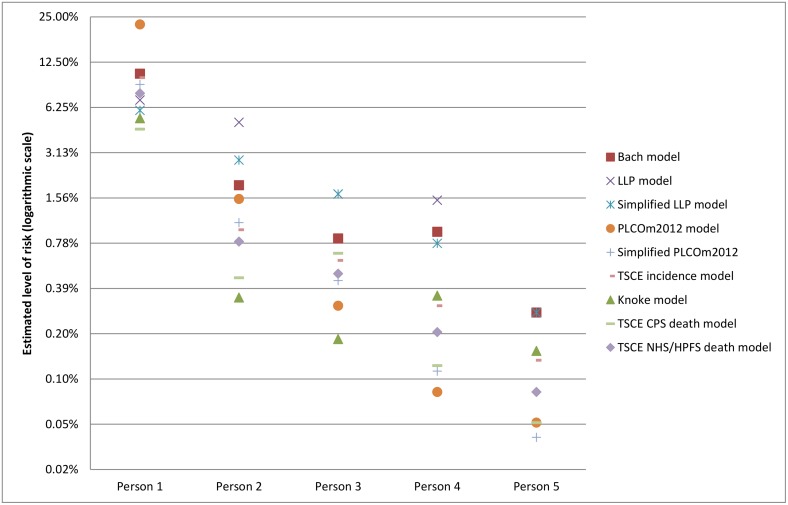
Examples of projected absolute risk for individuals with different risk factor profiles by model. Person 1: 70-y-old, high-school-graduated white male, current smoker, who smoked 30 cigarettes per day for 55 y, has a BMI of 28 kg/m^2^, has COPD, no asbestos exposure, no personal history of cancer, no personal history of pneumonia, but has a family history of lung cancer (relative was diagnosed at age > 60 y). Person 2: 63-y-old, college-graduated black woman, former smoker who quit 10 y ago, who smoked 15 cigarettes per day for 40 y, has a BMI of 25 kg/m^2^, does not have COPD, no asbestos exposure, no personal history of cancer, has a personal history of pneumonia, and no family history of lung cancer. Person 3: 65-y-old Asian male with some college education, former smoker who quit 14 y ago, who smoked 10 cigarettes per day for 30 y, has a BMI of 24 kg/m^2^, does not have COPD, has asbestos exposure, no personal history of cancer, no personal history of pneumonia, and no family history of lung cancer. Person 4: 58-y-old, post-graduate-educated Hispanic woman, current smoker, who smoked 5 cigarettes per day for 38 y, has a BMI of 22 kg/m^2^, does not have COPD, no asbestos exposure, has a personal history of cancer, no personal history of pneumonia, and no family history of lung cancer. Person 5: 50-y-old, college-educated white woman, current smoker, who smoked 5 cigarettes per day for 30 y, has a BMI of 22 kg/m^2^, does not have COPD, no asbestos exposure, no personal history of cancer, no personal history of pneumonia, and no family history of lung cancer. BMI, body mass index; COPD, chronic obstructive pulmonary disease; CPS, Cancer Prevention Study; HPFS, Health Professionals Follow-up Study; LLP, Liverpool Lung Project; NHS, Nurses’ Health Study; TSCE, Two-Stage Clonal Expansion.

The risk prediction models included in this study were developed in different populations (Table 1) and incorporate risk factors, specifically smoking behavior, in different ways (S1 Appendix). In addition, some models predict lung cancer incidence, while others predict lung cancer mortality. Therefore, the estimated absolute risk for the same individual varies between models [66]. Fig 1 shows the estimated 6-y risk of lung cancer incidence or mortality (depending on the target outcome of the model) across the models for five individuals with different risk factor profiles. This difference in estimated absolute risk between models suggests that specific risk thresholds might be needed for each model.

### Aspects of calibration performance

Overall, all models showed satisfactory calibration performance (S4 Appendix). The models showed the best calibration performance when they were applied to their target outcome, i.e., lung cancer incidence rather than lung cancer mortality for lung cancer incidence models. The calibration was better for all models in the PLCO datasets than in the NLST datasets.

### Discrimination

**Fig 2:**
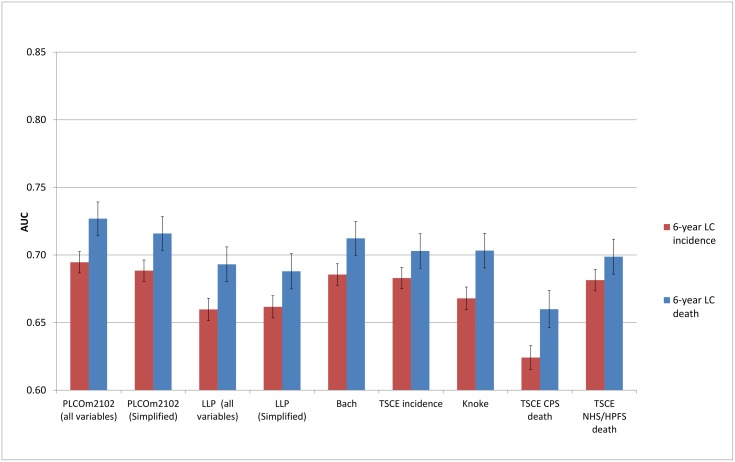
Area under the receiver operating characteristic curve of the investigated risk models (with 95% confidence interval) in the National Lung Screening Trial computed tomography arm by predicted outcome. AUC, area under the receiver operating characteristic curve; CPS, Cancer Prevention Study; HPFS, Health Professionals Follow-up Study; LC, lung cancer; LLP, Liverpool Lung Project; NHS, Nurses’ Health Study; TSCE, Two-Stage Clonal Expansion.

**Fig 3:**
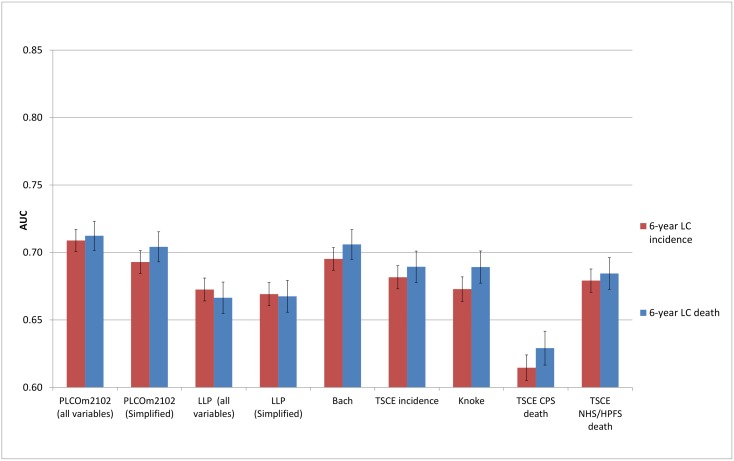
Area under the receiver operating characteristic curve of the investigated risk models (with 95% confidence interval) in the National Lung Screening Trial chest radiography arm by predicted outcome. AUC, area under the receiver operating characteristic curve; CPS, Cancer Prevention Study; HPFS, Health Professionals Follow-up Study; LC, lung cancer; LLP, Liverpool Lung Project; NHS, Nurses’ Health Study; TSCE, Two-Stage Clonal Expansion.

**Fig 4:**
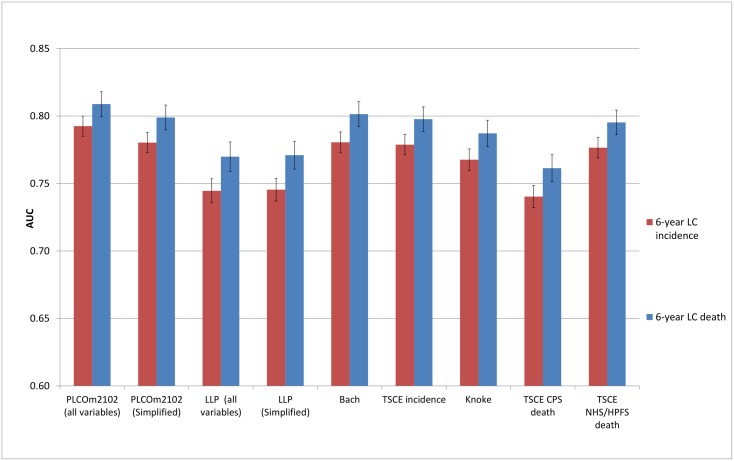
Area under the receiver operating characteristic curve of the investigated risk models (with 95% confidence interval) in the Prostate, Lung, Colorectal and Ovarian Cancer Screening Trial chest radiography arm by predicted outcome. AUC, area under the receiver operating characteristic curve; CPS, Cancer Prevention Study; HPFS, Health Professionals Follow-up Study; LC, lung cancer; LLP, Liverpool Lung Project; NHS, Nurses’ Health Study; TSCE, Two-Stage Clonal Expansion.

**Fig 5:**
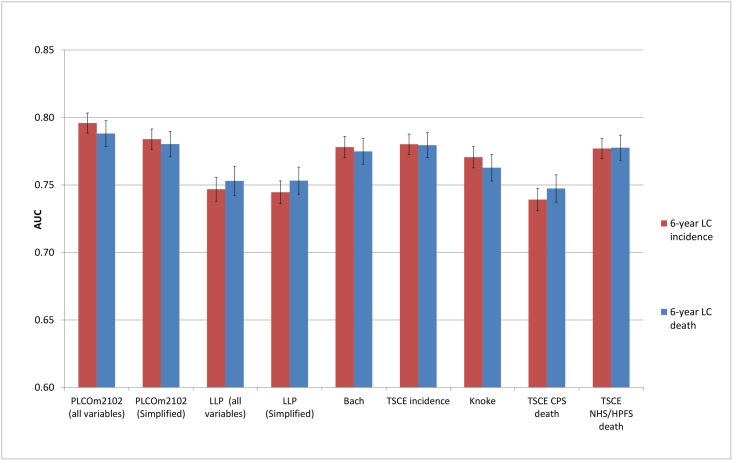
Area under the receiver operating characteristic curve of the investigated risk models (with 95% confidence interval) in the Prostate, Lung, Colorectal and Ovarian Cancer Screening Trial control arm by predicted outcome. AUC, area under the receiver operating characteristic curve; CPS, Cancer Prevention Study; HPFS, Health Professionals Follow-up Study; LC, lung cancer; LLP, Liverpool Lung Project; NHS, Nurses’ Health Study; TSCE, Two-Stage Clonal Expansion.

The discriminative performance of the models (Figs 2–5) was better in the PLCO datasets (AUCs ranging from 0.74 to 0.81) than in the NLST datasets (AUCs ranging from 0.61 to 0.73). The discriminative performance of most models was better for lung cancer mortality than for lung cancer incidence (i.e., the AUCs of most models were higher for lung cancer mortality than for lung cancer incidence) in all datasets, except for the PLCO control arm. The PLCOm2012 model (and its simplified version), the Bach model, and the TSCE incidence model showed the best discriminative performance across all datasets regardless of the type of predicted outcome. The discriminative performance of the models was similar for 5- and 6-y time frames, as shown in S5 Appendix.

### Clinical usefulness

Decision curve analysis for each risk prediction model provided a range of risk thresholds that yield a positive net benefit compared with the NLST eligibility criteria. Table 3 shows the lower and upper bounds for these ranges of risk thresholds for 6-y lung cancer incidence across all datasets. Overall, the lower and upper thresholds varied by model, but the ranges were roughly consistent across models, going from approximately 0.1% to 16.7%. This suggests that applying the models is useful for determining screening eligibility if missing one case of lung cancer that could be detected through screening is perceived as being between 999 and 5 times worse than unnecessarily screening one person. More detailed results for the decision curve analyses for both lung cancer incidence and mortality are shown in S6 Appendix.

**Table 3:** Lower and upper risk thresholds for which the risk prediction models have a positive net benefit compared with the National Lung Screening Trial criteria for 6-y lung cancer incidence.

Model	NLST computed tomography-arm		NLST chest radiography arm		PLCO chest radiography arm		PLCO control arm	
	Lower risk threshold (WF*)	Upper risk threshold (WF*)	Lower risk threshold (WF*)	Upper risk threshold (WF*)	Lower risk threshold (WF*)	Upper risk threshold (WF*)	Lower risk threshold (WF*)	Upper risk threshold (WF*)
Bach model	0.5% (199.0)	12.7% (6.9)	0.9% (110.1)	13.5% (6.4)	0.3% (332.3)	10.4% (8.6)	0.2% (499.0)	8.9% (10.2)
LLP model	1.8% (54.6)	8.0% (11.5)	1.2% (82.3)	7.3% (12.7)	0.4% (249.0)	6.5% (14.4)	0.4% (249.0)	5.8% (16.2)
Simplified LLP model	1.9% (51.6)	8.5% (10.8)	1.9% (51.6)	8.5% (10.8)	0.4% (249.0)	8.5% (10.8)	0.3% (332.3)	5.3% (17.9)
PLCOm2012 model	0.9% (110.1)	16.1% (5.2)	0.1% (999.0)	10.5% (8.5)	0.2% (499.0)	9.0% (10.1)	0.1% (999.0)	11.0% (8.1)
Simplified PLCOm2012 model	0.7% (141.9)	13.6% (6.4)	0.7% (141.9)	12.0% (7.3)	0.3% (332.3)	9.3% (9.8)	0.2% (499.0)	8.5% (10.8)
TSCE lung cancer incidence model	2.0% (49.0)	12.1% (7.3)	1.1% (89.9)	8.0% (11.5)	0.3% (332.3)	7.9% (11.7)	0.2% (499.0)	6.9% (13.5)
Knoke model	3.5% (27.6)	13.3% (6.5)	2.8% (34.7)	8.8% (10.4)	3.0% (32.3)	7.7% (12.0)	2.9% (33.5)	7.2% (12.9)
TSCE CPS lung cancer death model	3.4% (28.4)	7.1% (13.1)	2.8% (34.7)	6.2% (15.1)	2.8% (34.7)	6.2% (15.1)	2.8% (34.7)	6.0% (15.7)
TSCE NHS/HPFS lung cancer death model	2.7% (36.0)	16.7% (5.0)	2.0% (49.0)	9.9% (9.1)	0.2% (499.0)	7.8% (11.8)	2.3% (42.5)	6.6% (14.2)

*Weighting factor corresponding to the risk threshold, i.e., the ratio of how much worse missing one case of lung cancer that could be detected through screening is valued compared to unnecessarily screening one person.

CPS, Cancer Prevention Study; HPFS, Health Professionals Follow-up Study; LC, lung cancer; LLP, Liverpool Lung Project; NHS, Nurses’ Health Study; NLST, National Lung Screening Trial; PLCO, Prostate, Lung, Colorectal and Ovarian Cancer Screening Trial; TSCE, Two-Stage Clonal Expansion; WF, weighting factor.

### Comparison to National Lung Screening Trial eligibility criteria

**Fig 6:**
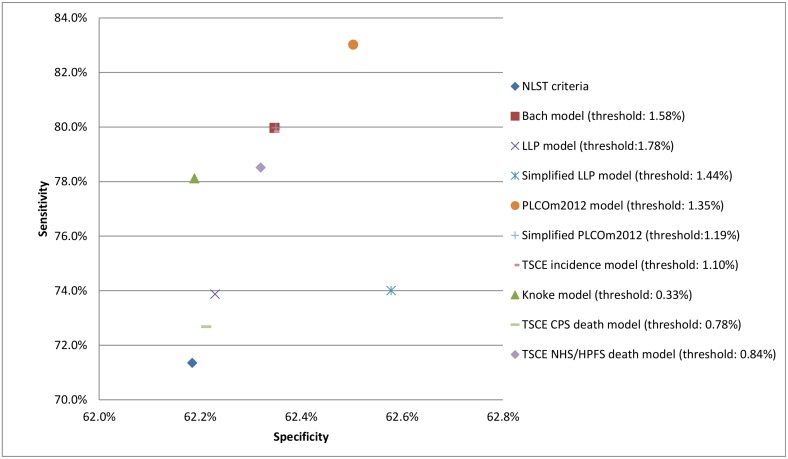
Sensitivity, specificity, and risk thresholds for the investigated risk prediction models and the National Lung Screening Trial criteria for 6-y lung cancer incidence in the Prostate, Lung, Colorectal and Ovarian Cancer Screening Trial chest radiography arm. CPS, Cancer Prevention Study; HPFS, Health Professionals Follow-up Study; LLP, Liverpool Lung Project; NHS, Nurses’ Health Study; NLST, National Lung Screening Trial; TSCE, Two-Stage Clonal Expansion.

Applying the NLST eligibility criteria yielded a sensitivity of 71.4% (95% confidence interval: 68.0%–74.6%) and a specificity of 62.2% (95% confidence interval: 61.7%–62.7%) for 6-y lung cancer incidence in the PLCO CXR arm (Fig 6; Table 4). The sensitivity and specificity of each of the risk prediction models were higher than those of the NLST eligibility criteria. The PLCOm2012 model, in particular, followed by the Bach model and the TSCE incidence model had the highest sensitivities (all three models >79.8%) and specificities (all three models >62.3%) among all evaluated models. Fig 6 also shows the risk thresholds for each model that select a similar number of individuals for screening as the NLST eligibility criteria. Similar results were found for the PLCO control arm and for using 6-y lung cancer death as the outcome measure (S7 Appendix).

**Table 4:** Sensitivities and specificities corresponding to the suggested risk thresholds for the investigated risk prediction models and the National Lung Screening Trial criteria for 6-y lung cancer incidence in the Prostate, Lung, Colorectal and Ovarian Cancer Screening Trial chest radiography arm.

Measure	NLST criteria	Bach model	LLP model	Simplified LLP model	PLCOm2012 model	Simplified PLCOm2012 model	TSCE incidence model	Knoke model	TSCE CPS lung cancer death model	TSCE NHS/HPFS lung cancer death model
Sensitivity	71.4% (68.0%–74.6%)	80.0% (76.9%–82.8%)	73.9% (70.6%–77.0%)	74.0% (70.7%–77.1%)	83.0% (80.2%–85.6%)	80.0% (76.9%–82.8%)	79.8% (76.8%–82.6%)	78.1% (75.0%–81.0%)	72.7% (69.3%–75.8%)	78.5% (75.4%–81.4%)
Specificity	62.2% (61.7%–62.7%)	62.4% (61.9%–62.8%)	62.2% (61.8%–62.7%)	62.6% (62.1%–63.1%)	62.5% (61.9%–62.8%)	62.4% (61.9%–62.8%)	62.3% (61.9%–62.8%)	62.2% (61.7%–62.7%)	62.2% (61.7%–62.7%)	62.3% (61.8%–62.8%)

Data given as percent (95% confidence interval).

CPS, Cancer Prevention Study; HPFS, Health Professionals Follow-up Study; LLP, Liverpool Lung Project; NHS, Nurses’ Health Study; NLST, National Lung Screening Trial; TSCE, Two-Stage Clonal Expansion.

## Discussion

This study assessed the performance of nine lung cancer risk prediction models in two large randomized controlled trials: the NLST and the PLCO. The models had satisfactory calibration, had modest to good discrimination, and provided a substantial range of risk thresholds with a positive net benefit compared with the NLST eligibility criteria. Given appropriate model-specific risk thresholds, all risk prediction models had a better sensitivity and specificity than the NLST eligibility criteria. This implies that lung cancer risk prediction models, when coupled with model-specific risk thresholds, outperform currently recommended lung cancer screening eligibility criteria (Tables 3 and 4; Fig 6).

The risk prediction models considered in this study were developed in various cohorts for different outcome measures (lung cancer incidence versus mortality), with fundamental differences in model structures. Consequently, the absolute risk estimates differed between models, which led to differences in calibration performance between the models, specifically in the NLST cohorts. In addition, there were clear differences in discriminative ability between the models. The discriminative ability of all models was better in the PLCO cohorts than in the NLST cohorts, which may be caused by the higher heterogeneity in risk factor profiles among individuals in the PLCO compared with the NLST [67,68]. The NLST required individuals to have smoked at least 30 pack-years and included only current and former smokers (who quit less than 15 y ago), whereas the PLCO did not have any criteria for enrollment with regards to smoking history. In line with these criteria, the average NLST participant had a higher lung cancer risk than the average PLCO participant. The results of our investigation suggest that the discriminative ability of the evaluated models may be lower in groups at elevated risk, which may be due to the lower heterogeneity in risk among participants in these groups [67,68]. However, randomized clinical trials suggest that the results of CT screening may provide an opportunity to improve risk stratification in these groups. In the NLST, participants with a negative prevalence screen had a substantially lower risk of developing lung cancer than participants with a positive prevalence screen [69]. Similarly, in the NELSON trial, the 2-y probability of developing lung cancer after a CT screen varied substantially by pulmonary nodule size and the volume doubling time of these pulmonary nodules [8]. Therefore, incorporating the results of CT screening could improve the risk stratification in groups of individuals at elevated risk. Finally, while there was little difference in specificity between the models at risk thresholds similar to the NLST eligibility criteria, there was a clear difference in sensitivity. In particular, the PLCOm2012 model, followed by the Bach model and the TSCE incidence model, had the best performance across all aspects investigated in this study.

Previous studies have also compared the performance of different lung cancer risk prediction models [20,21]. D’Amelio et al. examined the discriminatory performance of three risk prediction models for lung cancer incidence in a case–control study and found modest differences between the models [20]. However, this study considered a limited number of participants (1,066 cases and 677 controls) and did not consider other aspects of model performance such as calibration or clinical usefulness. Li et al. examined four risk prediction models for lung cancer incidence in German participants of the European Prospective Investigation into Cancer and Nutrition cohort [21]. They found that while the differences between most of the evaluated models were modest, generally only the Bach and the PLCOm2012 models had similar or better sensitivity and specificity compared to the eligibility criteria used in the NLST and other eligibility criteria that were used in various European lung cancer screening trials (which applied less restrictive smoking eligibility criteria than the NLST). This cohort consisted of 20,700 individuals, but fewer than 100 lung cancer cases occurred, which limits statistical power for external validation [18,19].

In contrast to these previous studies, we performed a comprehensive validation, including aspects of calibration, discriminative ability, and clinical usefulness, for many models, in a large sample (*n =* 134,124) with 3,388 lung cancer cases and 1,799 lung cancer deaths. In addition, while our study supports earlier findings that risk prediction models outperform the NLST eligibility criteria, it also suggests that the PLCOm2012 model followed by the Bach and TSCE incidence models perform better than other models in all investigated aspects.

Our study has some limitations. While our results provide indications regarding at which risk thresholds the investigated risk models can be clinically useful, the optimal thresholds to apply remain uncertain. Determining optimal thresholds requires information on the long-term benefits (such as life-years gained and mortality reduction) and harms (such as overdiagnosis) of applying these thresholds [60]. Natural history modeling may provide further information on the trade-off between the long-term benefits and harms for screening programs with different risk thresholds, similarly to how our previous study informed the USPSTF on its recommendations for lung cancer screening [2].

Another limitation is that information on some of the predictor variables included in the evaluated risk prediction models was not available in the NLST and the PLCO, e.g., asbestos exposure was missing in both cohorts. However, only a few variables were unavailable. Furthermore, some of the evaluated models that used only age, gender, and smoking behavior, such as the TSCE models and the Knoke model, performed similarly to the other models that used additional information on risk factors, suggesting that age, gender, and smoking behavior are the most important risk factors for lung cancer. Thus, the improved performance of these models over the NLST eligibility criteria may primarily be due to the inclusion of detailed smoking behavior in these models. The NLST eligibility criteria use a dichotomized criterion for accumulated pack-years, e.g., an exposure of at least 30 pack-years, which leads to a loss of information for continuous variables [70]. Furthermore, pack-years are estimated by smoking duration and intensity (cigarettes per day), and previous studies indicate that both components contribute independently to an individual’s risk for developing lung cancer; an aggregation of both may not fully capture the effects of smoking on lung cancer risk [10,43,71].

We chose to evaluate the models for varying follow-up lengths (5- and 6-y time frames) to investigate the effect of follow-up duration on the discrimination performance of each model [54]. Although the discriminative performance of the models was similar for 5- and 6-y time frames (S5 Appendix), this may not be the case for more disparate time frames.

A number of pertinent questions remain with regards to the implementation of lung cancer screening [9]. Current guidelines like the USPSTF recommendations suggest that individuals should be asked, at a minimum, about their age and smoking history [3]. A number of the models evaluated in our study use information on additional risk factors, such as personal history of cancer, which could be a potential barrier for implementing lung cancer screening based on risk prediction models. However, the LLP and PLCOm2012 models were successfully used to recruit individuals for the UK Lung Cancer Screening Trial (UKLS) and the Pan-Canadian Early Detection of Lung Cancer Study (PanCan), respectively, through short questionnaires [33,72]. This suggests that acquiring information on the risk factors required for these models does not pose a major barrier for implementation. Furthermore, for some risk models, such as the Bach and PLCOm2012 models, online calculators are available, which provide opportunities for fast risk estimation in clinical practice [73–76]. For example, the PLCOm2012 model has been embedded in a lung cancer screening decision aid that has been widely adopted and that can be used to satisfy the Centers for Medicare & Medicaid Services reimbursement requirement for shared decision making [75–77].

In conclusion, our study suggests that lung cancer screening selection criteria can be improved through the explicit application of risk prediction models rather than using criteria based on age and pack-years as a summary measure of smoking exposure. These models might also be helpful for improving the shared decision-making process for lung cancer screening recommended by the USPSTF and required in the US by the Centers for Medicare & Medicaid Services [3,75,78]. However, recommendations for the implementation of risk-based lung cancer screening require a thorough evaluation of the benefits and harms of risk-based screening, as well as an assessment of the feasibility of implementing strategies based on risk models. Therefore, future studies need to evaluate the long-term benefits and harms of applying risk prediction models at different risk thresholds, while considering the potential challenges for implementation, and compare these with the expected benefits and harms of current guidelines.

## Supporting information

S1 Appendix Lung cancer risk prediction model descriptions. (DOCX)

S2 Appendix National Lung Screening Trial and Prostate, Lung, Colorectal and Ovarian Cancer Screening Trial participant characteristics. (DOCX)

S3 Appendix Overview of National Lung Screening Trial and Prostate, Lung, Colorectal and Ovarian Cancer Screening Trial participants with complete information. (DOCX)

S4 Appendix Calibration aspects of the evaluated lung cancer risk prediction models for 6-y lung cancer incidence and mortality. (DOCX)

S5 Appendix Discriminative performance of the investigated risk models, by dataset, predicted outcome, and time frame. (DOCX)

S6 Appendix Decision curve analyses for the evaluated lung cancer risk prediction models for 6-y lung cancer incidence and mortality. (DOCX)

S7 Appendix Comparison of the evaluated lung cancer risk prediction models at risk thresholds similar to the National Lung Screening Trial eligibility criteria. (DOCX)

S8 Appendix TRIPOD checklist. (DOCX)

## Abbreviations

AUC: area under the receiver operating characteristic curve
CT: computed tomography
CXR: chest radiography
LLP: Liverpool Lung Project
NLST: National Lung Screening Trial
PLCO: Prostate, Lung, Colorectal and Ovarian Cancer Screening Trial
TSCE: Two-Stage Clonal Expansion
USPSTF: United States Preventive Services Task Force
