# Correction: Risk prediction models for selection of lung cancer screening candidates: A retrospective validation study

**DOI:** 10.1371/journal.pmed.1003403

**Published:** 2020-09-25

**Authors:** Kevin ten Haaf, Jihyoun Jeon, Martin C. Tammemägi, Summer S. Han, Chung Yin Kong, Sylvia K Plevritis, Eric J. Feuer, Harry J. de Koning, Ewout W. Steyerberg, Rafael Meza

The authors discovered a typographical error in S1 Appendix, page 2, regarding the description of one of the risk-prediction models (the Bach model) used. Some mathematical operators (+/- signs) were inadvertently reversed in the description of the 1-year lung cancer probability.

The mathematical operators associated with the "SMK" terms of the equation should have read:

+ (0.11425297 * SMK 〖- (0.000080091477 * (SMK– 27.6577))〗^3 for all values SMK>27

instead of + (0.11425297 * SMK 〖+ (0.000080091477 * (SMK– 27.6577))〗^3 for all values SMK>27

,

+(0.00017069483* (SMK– 40)^3) for all values SMK>40

instead of–(0.00017069483* (SMK– 40)^3) for all values SMK>40

,

-(0.000090603358* (SMK-50.910335)^3) for all values SMK>50

instead of +(0.000090603358* (SMK-50.910335)^3) for all values SMK>50

While the operators associated with the "Age"terms of the equation should have read:

+ (0.070322812 * AGE〖 - (0.00009382122* (AGE– 53.459001))〗^3 for all values AGE>53

instead of + (0.070322812 * AGE〖 + (0.00009382122* (AGE– 53.459001))〗^3 for all values AGE>53

+(0.00018282661* (AGE– 61.954825)^3) for all values AGE>61

instead of–(0.00018282661* (AGE– 61.954825)^3) for all values AGE>61

-(0.000089005389* (AGE– 70.910335)^3) for all values AGE>70

instead of +(0.000089005389* (AGE– 70.910335)^3) for all values AGE>70

The typographical error was only present in the supplementary description, the equations were correctly specified in the code of the Bach model that was applied for the analyses demonstrated throughout the main paper and the supplementary material. Please see the corrected version of S1 Appendix.

## Supporting information

S1 Appendix Lung cancer risk prediction model descriptions. (DOCX)

## References

[1] ten Haaf K, Jeon J, Tammemägi MC, Han SS, Kong CY, Plevritis SK, et al (2017) Risk prediction models for selection of lung cancer screening candidates: A retrospective validation study. PLoS Med 14(4): e1002277 10.1371/journal.pmed.1002277 28376113 PMC5380315

