# Supplementary material for: Risk prediction models for selection of lung cancer screening candidates: A retrospective validation study
Source: PLoS Med. 2017 Apr 4;14(4):e1002277. doi: 10.1371/journal.pmed.1002277 (PMC5380315; doi:10.1371/journal.pmed.1002277)
Supplement: S1 Appendix — (DOCX) [file pmed.1002277.s001.docx]

**S1 Appendix: Lung cancer risk prediction model descriptions**

**Description of the Bach model**

The Bach model was developed in the Carotene and Retinol Efficacy Trial (CARET) using 36,286 individuals (1,070 lung cancer cases) [[1](#_ENREF_1)]. The Bach model consists of two components, i.e. a model for lung cancer diagnosis and a model for death in the absence of lung cancer diagnosis, estimated through Cox proportional hazards regression, and together predict lung cancer incidence for a 1-year timeframe (as a binary outcome event). Applying the models iteratively allows for predictions over longer timeframes. The model predictors include: age, gender, asbestos exposure, smoking intensity (cigarettes per day), smoking duration and quit-time in former smokers. The component for estimating the one-year probability of death in the absence of lung cancer diagnosis is:

$$1-S_{0}^{e(model)}$$

With $S_{0}=0.9917663$ and $model$ being represented by the following equation, where CPD = cigarettes per day, SMK = duration of smoking, QUIT = duration of quitting, AGE = age, ASB = asbestos exposure, and GENDER = gender;

$-7.2036219 + (0.015490665 * CPD)$ $-(0.00001737645 * \left( CPD - 15 \right)^{3})$ for all values CPD>15

$+(0.000021924149 * \left( CPD - 20.185718 \right)^{3})$ for all values CPD>20

$-(0.0000045476985* \left( CPD - 40 \right)^{3})$ for all values CPD>40

$+ (0.020041889 * SMK$ ${+ (0.0000065443781 * (SMK - 27.6577))}^{3}$ for all values SMK>27

$-(0.000013947696* \left( SMK - 40 \right)^{3})$ for all values SMK>40

$+(0.0000074033175* \left( SMK-50.910335 \right)^{3})$ for all values SMK>50

$- (0.023358962 * QUIT$ ${+ ( (0.0019208669 * QUIT)}^{3})$ for all values

$-(0.0020031611 * \left( QUIT - 0.50513347 \right)^{3})$ for all values QUIT>0

$+(0.000082294194 * \left( QUIT - 12.295688 \right)^{3})$ for all values QUIT>12

$+ (0.099168033 * AGE{+ (0.0000062174577* (AGE - 53.459001))}^{3}$ for all values AGE>53

$-(0.000012115774* \left( AGE - 61.954825 \right)^{3})$ for all values AGE>61

$+(0.0000058983164 * \left( AGE - 70.910335 \right)^{3})$ for all values AGE>70

$+ (0.06084611$) if ASB = yes

$- (0.49042298$) if GENDER=female

The component for estimating the one-year probability diagnosis of lung cancer is:

$$1-S_{0}^{e(model)}$$

With $S_{0}=0.99629$ and $model$ being represented by the following equation, where CPD = cigarettes per day, SMK = duration of smoking, QUIT = duration of quitting, AGE = age, ASB = asbestos exposure, and GENDER = gender;

$-9.7960571 + (0.060818386 * CPD)$ $-(0.00014652216 * \left( CPD - 15 \right)^{3})$ for all values CPD>15

$+(0.00018486938 * \left( CPD - 20.185718 \right)^{3})$ for all values CPD>20

$-(0.000038347226 * \left( CPD - 40 \right)^{3})$ for all values CPD>40

$+ (0.11425297 * SMK$ ${+ (0.000080091477 * (SMK - 27.6577))}^{3}$ for all values SMK>27

$-(0.00017069483* \left( SMK - 40 \right)^{3})$ for all values SMK>40

$+(0.000090603358* \left( SMK-50.910335 \right)^{3})$ for all values SMK>50

$- (0.085684793 * QUIT$ ${+ ( (0.0065499693 * QUIT)}^{3})$ for all values

$-(0.0068305845 * \left( QUIT - 0.50513347 \right)^{3})$ for all values QUIT>0

$+(0.00028061519* \left( QUIT - 12.295688 \right)^{3})$ for all values QUIT>12

$+ (0.070322812 * AGE{+ (0.00009382122* (AGE - 53.459001))}^{3}$ for all values AGE>53

$-(0.00018282661* \left( AGE - 61.954825 \right)^{3})$ for all values AGE>61

$+(0.000089005389* \left( AGE - 70.910335 \right)^{3})$ for all values AGE>70

$+ (0.2153936$) if ASB = yes

$- (0.0582726$) if GENDER=female

The model has been externally by the authors in 6,239 smokers (with 333 lung cancer cases) from the placebo arm of the Alpha-Tocopherol, Beta-Carotene Cancer Prevention (ATBC) Study [[2](#_ENREF_2)].

**Description of the Liverpool Lung Project (LLP) model**

The Liverpool Lung Project (LLP) model was based on data from the Liverpool Lung Project case-control study [[3](#_ENREF_3)]. The model was estimated through multivariate conditional logistic regression and predicts lung cancer incidence for a 5-year timeframe using 579 individuals with lung cancer and 1,157 age- and gender-matched population-based controls. The risk factors incorporated in the model are listed in Table A, along with their log odds ratios and corresponding model coefficients.

As the model intercept could not be estimated using case-control data, the authors derived the age-group and gender-specific model intercepts through age- and gender-specific lung cancer incidence rates in the Liverpool area, as shown in Table B [[3](#_ENREF_3)]. The model intercept for an individual aged *x*+*y* years, where *x* is a multiple of 5 and *y* is 0, 1, 2, 3 or 4, can be calculated as follows:

${Intercept}_{Age x+y, sex}=\frac{\left( 5-y-0.5 \right)*{Intercept}_{Age x, sex}+\left( y+0.5 \right)*{Intercept}_{Age x+5, sex}}{5}$

The model has been externally validated twice by the authors: once in 1,066 cases and 677 controls treated at the Thoracic Surgery, Thoracic Oncology, or Pulmonary Units at the Massachusetts General Hospital (Boston, MA, USA) and once in 585 cases and 1,283 controls from the European Early Lung Cancer case–control study, 1,738 cases and 1,184 controls from the Harvard case–control study and 7,652 individuals (with 420 lung cancer cases) from the Liverpool Lung Project Cohort study [[4](#_ENREF_4),[5](#_ENREF_5)].

**Table A: Risk factors considered in the Liverpool Lung Project model**

| **Risk factor** | **Log odds ratio** | **Model coefficient** |
| --- | --- | --- |
| *Smoking duration* |  |  |
| Never | 1.00 (reference) | 0.000 (reference) |
| 1-20 years | 2.16 | 0.769 |
| 21-40 years | 4.27 | 1.452 |
| 41-60 years | 12.27 | 2.507 |
| >60 years | 15.25 | 2.724 |
| *Prior diagnosis of pneumonia* |  |  |
| No | 1.00 (reference) | 0.000 (reference) |
| Yes | 1.83 | 0.602 |
| *Occupational exposure to asbestos* |  |  |
| No | 1.00 (reference) | 0.000 (reference) |
| Yes | 1.89 | 0.634 |
| *Prior diagnosis of malignant tumor* |  |  |
| No | 1.00 (reference) | 0.000 (reference) |
| Yes | 1.96 | 0.675 |
| *Family history of lung cancer* |  |  |
| No | 1.00 (reference) | 0.000 (reference) |
| Early onset (age < 60 years) | 2.02 | 0.703 |
| Late onset (age ≥ 60 years) | 1.18 | 0.168 |

**Table B: Age- and gender-specific lung cancer incidence rates (per 100,000 person-years) in the Liverpool area (2002-2004)**

|  | **Men** | | **Women** | |
| --- | --- | --- | --- | --- |
| **Age-group** | **Lung cancer incidence rate** | **Corresponding model intercept** | **Lung cancer incidence rate** | **Corresponding model intercept** |
| 40-44 | 15.5 | -9.06 | 5.97 | -9.90 |
| 45-49 | 37.87 | -8.16 | 37.34 | -8.06 |
| 50-54 | 88.65 | -7.31 | 68.14 | -7.46 |
| 55-59 | 172.26 | -6.63 | 175.24 | -6.50 |
| 60-64 | 329.02 | -5.97 | 230.60 | -6.22 |
| 65-69 | 487.42 | -5.56 | 288.06 | -5.99 |
| 70-74 | 616.45 | -5.31 | 464.99 | -5.49 |
| 75-79 | 950.61 | -4.83 | 594.19 | -5.23 |
| 80-84 | 1096.42 | -4.68 | 497.09 | -5.42 |

**Description of the simplified Liverpool Lung Project (LLP) model**

The simplified version of the LLP model uses the same parameter estimates as the original LLP model. However, when applying this model to a participant, it is assumed that only information on age and smoking history is known. Thus, the simplified model assumes that the participant had no prior diagnosis of pneumonia, no occupational exposure to asbestos, no prior diagnosis of a malignant tumor and no family history of lung cancer.

**Description of the PLCOm2012 model**

The PLCOm2012 model was developed in the control arm of the Prostate, Lung, Colorectal and Ovarian Cancer Screening Trial (PLCO), using 36,286 individuals (630 lung cancer cases) [[6](#_ENREF_6)]. The model was estimated through multivariate logistic regression and predicts lung cancer incidence for a 6-year timeframe. It was initially validated in 37,332 individuals (678 lung cancer cases) of the PLCO intervention arm (in which chest radiography screening occurred).

The model predictors include seven non-smoking variables: age, race/ethnicity, education (an estimator of socioeconomic circumstance), body mass index, personal history of cancer, family history of lung cancer and chronic obstructive pulmonary disease. The model includes four smoking variables: smoking status (former vs. current), smoking intensity (cigarettes per day), smoking duration and quit-time in former smokers. Using multivariable fractional polynomials, smoking intensity was shown to have a nonlinear relationship with lung cancer, and this nonlinear effect is incorporated into PLCOm2012. The risk factors incorporated in the model are listed in Table C, along with their log odds ratios and corresponding model coefficients.

The initial predictive performance evaluation of the PLCOm2012 in the PLCO intervention arm demonstrated high discrimination (AUC = 0.80) and calibration (predicted probabilities / observed = 0.95). The model has also been externally validated by the authors in 51,033 (1,826 cases) participants of the National Lung Screening Trial [[6](#_ENREF_6)].

**Table C: Risk factors considered in the PLCOm2012 model**

| **Risk factor** | **Log odds ratio** | **Model coefficient** |
| --- | --- | --- |
| Age, per 1-year increase (centered on age 62) | 1.081 | 0.0778868 |
| *Race or ethnic group (self-reported)* |  |  |
| White (non-Hispanic) | 1.00 (reference) | 0.000 (reference) |
| Black (non-Hispanic) | 1.484 | 0.3944778 |
| Hispanic | 0.475 | −0.7434744 |
| Asian | 0.627 | −0.466585 |
| Native Hawaiian or Pacific Islander | 1.00 | 0.000 |
| American Indian or Alaskan Native | 2.793 | 1.027152 |
| Education, per increase of 1 level. Education was centered on level 4* | 0.922 | −0.0812744 |
| Body-mass index, per 1-unit increase (centered on 27) | 0.973 | −0.0274194 |
| *Chronic obstructive pulmonary disease* |  |  |
| No | 1.00 (reference) | 0.000 (reference) |
| Yes | 1.427 | 0.3553063 |
| *Personal history of cancer* |  |  |
| No | 1.00 (reference) | 0.000 (reference) |
| Yes | 1.582 | 0.4589971 |
| *Family history of lung cancer* |  |  |
| No | 1.00 (reference) | 0.000 (reference) |
| Yes | 1.799 | 0.587185 |
| *Smoking status* |  |  |
| Former | 1.00 (reference) | 0.000 (reference) |
| Current | 1.297 | 0.2597431 |
| Smoking intensity** | This variable is nonlinear so no single odds ratio represents the entire association | −1.822606 |
| Duration of smoking, per 1-year increase (centered on 27 years) | 1.032 | 0.0317321 |
| Smoking quit time, per 1-year increase (centered on 10 years) | 0.970 | −0.0308572 |
| Model constant |  | −4.532506 |

**Table notes:**

* Education was measured in six ordinal levels: less than high-school graduate (level 1), high-school graduate (level 2), some training after high school (level 3), some college (level 4), college graduate (level 5), and postgraduate or professional degree (level 6).

** For smoking intensity, the contribution of the variable to the model should be calculated by dividing the number of cigarettes per day by 10, exponentiating by the power −1, centering by subtracting 0.4021541613, and multiplying this number by the beta coefficient of the variable.

**Description of the Simplified PLCOm2102 model**

The simplified version of the PLCOm2012 model uses the same parameter estimates as the original PLCOm2012 model. However, similarly to the simplified LLP model, it is assumed that only information on age and smoking history is known. Thus, the simplified model assumes that the participant was white, had a body mass index of 27 (center value), some college education (center value), no chronic obstructive pulmonary disease, no personal history of cancer, and no family history of lung cancer.

**Description of the Two-Stage Clonal Expansion (TSCE) models**

The Two-Stage Clonal Expansion (TSCE) model represents the process of carcinogenesis in three phases. In the first phase (initiation), a susceptible stem cell acquires one or more mutations resulting in an initiated cell, which has partially escaped growth control. In the second phase (promotion), initiated cells undergo clonal expansion, either spontaneously or in response to endogenous or exogenous promoters. Finally, in the third phase (malignant conversion), one of the initiated cells acquires further mutational changes leading to a malignant cell.

The TSCE model assumes that normal stem cells become initiated according to a Poisson process with intensity “νX”, where X is the number of normal stem cells (X) in the lungs and “ν” represents the initiation rate of normal stem cells. Once a cell is initiated, it undergoes a stochastic clonal expansion (promotion) with cell division rate “α” and cell death/differentiation rate “β”. An initiated cell can also divide into one initiated and one malignant cell (malignant conversion) with rate “μ”. A constant lag time, or lag time distribution, is commonly used to represent the time between the onset of the first malignant cell and lung cancer incidence or mortality depending on the model outcome (progression). Figure A shows the pictorial description of the TSCE model.

To model the effects of smoking on lung cancer risk, the model initiation, promotion, and malignant transformation parameters are assumed to be altered during periods of smoking exposure through flexible dose-response relationships:

$$\theta\left( t \right)=\theta_{0}\times\left( 1+\theta_{1}\times{d\left( t \right)}^{\theta_{2}} \right),$$

where θ represents identifiable biological parameters (i.e., combinations of ν, α, β, μ, and X), θ_0_ the background rate, θ_1_ the dose-response coefficient, θ_2_ the non-linearity of the dose-response, and d(t) is smoking dose at time t. This dose-response relationship links the individual smoking history to the cell kinetic parameters in the TSCE model. The TSCE model was previously calibrated to lung cancer incidence or mortality data in several large prospective smoking cohorts: the Nurses' Health Study (NHS) for women, the Health Professionals Follow-up Study (HPFS) for men [[7](#_ENREF_7),[8](#_ENREF_8)], the British doctors cohort, the American Cancer Society Cancer Prevention Studies I and II (CPS-I and CPS-II) cohorts [[9](#_ENREF_9)]. Here we used the CPS-I, NHS, and HPFS versions of the model. Tables D and E present the structure and parameter values for each of the different TSCE model versions. To compute the 6-year probability to develop lung cancer (for incidence models) or die from lung cancer (for mortality models) given that the person survives by the age at the entry (say t_a_), we assume that the smoking patterns at the entry remain the same for the next 6 years of follow-up. The conditional 6-year probability is computed by

$$\Pr(T\leq t_{a}+6|T>t_{a})=\frac{\Pr(t_{a}<T\leq t_{a}+6)}{\Pr(T>t_{a})}=1-\frac{S(t_{a}+6)}{S(t_{a})},$$

where S(t) is the survival function from the TSCE model [[10](#_ENREF_10)].

**Figure A: Representation of the Two-Stage Clonal Expansion model**


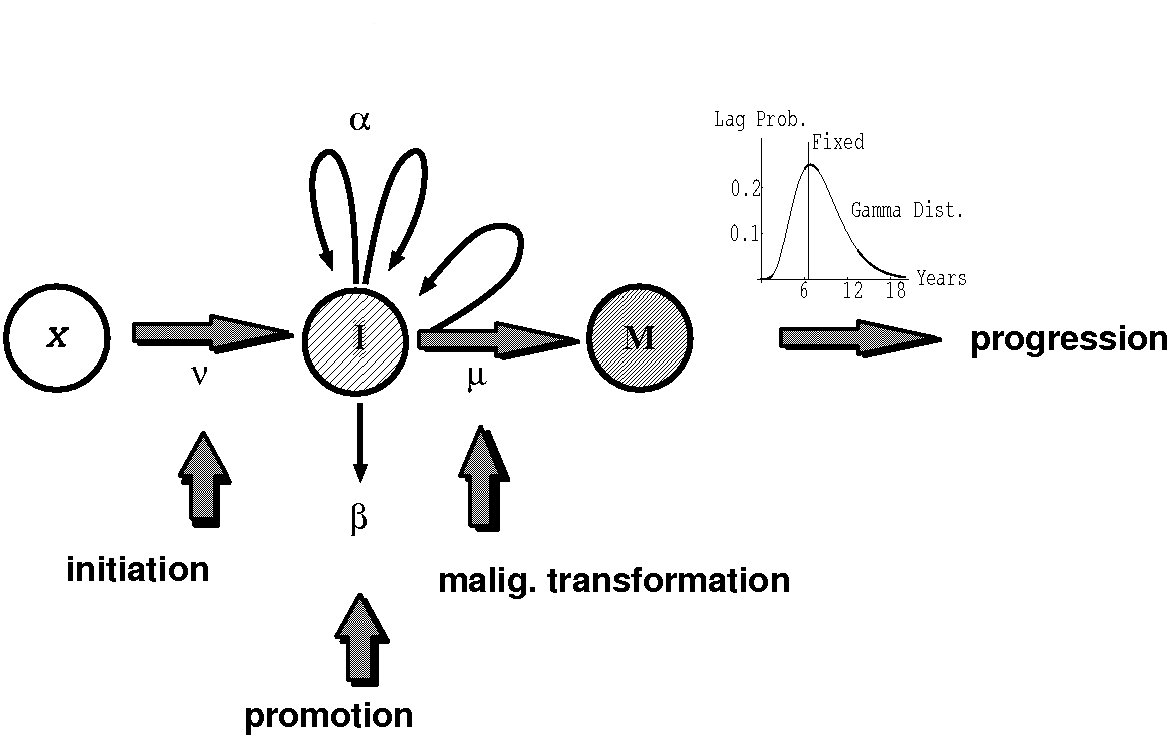


**Figure notes:** The Two-Stage Clonal Expansion (TSCE) model is a stochastic representation of the cell events during carcinogenesis. The carcinogenic process may be thought of as consisting of three phases: initiation,

promotion, and malignant transformation. Normal stem cells (labeled *X*) may mutate at rate ν to create an initiated cell (labeled *I*). An initiated cell may divide at rate α, die or differentiate at rate β, and mutate at rate μ to create a malignant cell. A lag time, or lag time distribution, is used to represent the time from occurrence of the first malignant cell to lung cancer death.

**Table D: Model parameters for background rate and dose –response relationship**

| **Background variables for CPS-I, NHS, and HPFS models** | |
| --- | --- |
| X=10^7^ | Assume 10^7^ normal stem cells in both lungs |
| α_0_ | Background cell division rate (per cell per year) |
| g_0_=α_0_-β_0_-μ_0_ | Background net cell promotion rate (per cell per year) |
| ν_0_=μ_0_ | Background initiation rate; Background malignant transformation rate (per cell per year) |
| t_lag_=5 years | Fixed constant lag time |
| **Dose-response variables** | |
| **CPS-I model** | |
| ν_i_=ν_0_(1+p_1_); p_1_=0 for nonsmokers | Initiation rate (per cell per year) |
| g_i_=g_0_(1+p_2_×dose_i_^p3^) | Net initiated cell promotion rate (per cell per year) |
| α_i_=α_0_(1+p_2_×dose_i_^p3^) | Initiated cell division rate (per cell per year) |
| μ_i_=μ_0_ | Malignant transformation rate (per cell per year); No dose-response |
| **NHS and HPFS models** | |
| ν_i_=ν_0_ | Initiation rate (per cell per year); No dose-response |
| g_i_=g_0_(1+p_2_×dose_i_^p3^) | Net initiated cell promotion rate (per cell per year) |
| α_i_=α_0_(1+p_2_×dose_i_^p3^) | Initiated cell division rate (per cell per year) |
| μ_i_=μ_0_(1+p_4_×dose_i_^p5^) | Malignant transformation rate (per cell per year) |

**Table E: Parameter estimates for CPS-I, NHS, and HPFS models**

| Model | α_0_ | g_0_ | ν_0_ (=μ_0_) | p_1_ | p_2_ | p_3_ | p_4_ | p_5_ |
| --- | --- | --- | --- | --- | --- | --- | --- | --- |
| **Lung Cancer mortality models** | | | | | | | | |
| CPS-I females | 71.56 | 0.086 | 8.93×10^-8^ | 1.23 | 0.04 | 0.98 | - | - |
| CPS-I  males | 22.65 | 0.075 | 1.40×10^-7^ | 1.79 | 0.21 | 0.47 | - | - |
| NHS | 3.00 | 0.076 | 1.03×10^-7^ | - | 0.20 | 0.50 | 0.05 | 0.60 |
| HPFS | 3.00 | 0.076 | 1.03×10^-7^ | - | 0.33 | 0.35 | 0.21 | 0.18 |
| **Lung Cancer incidence models** | | | | | | | | |
| NHS | 3.00 | 0.077 | 1.26×10^-7^ | - | 0.17 | 0.53 | 0.10 | 0.62 |
| HPFS | 3.00 | 0.077 | 1.26×10^-7^ | - | 0.26 | 0.39 | 0.33 | 0.25 |

**Description of the Knoke model**

The Knoke model was developed using lung cancer mortality data from the American Cancer Society Cancer Prevention Study I (CPS-I) [[11](#_ENREF_11)]. The model is specific for white males of ages 40-79. For never smokers, the absolute risk of death due to lung cancer was modeled as a two-parameter Poisson regression model on attained age in years:

$$R_{NS}=9.21\times10^{-13}\times age^{4.6}$$

For continuing smokers, the excess risk of death due to lung cancer was modeled as a Poisson regression model with modified offset, assuming the mean value function to be a power function:

$$ER_{S}=1.51\times10^{-13}\times age^{2.38}\times CPD^{0.867}\times dur^{2.87},$$

where *CPD* is the number of cigarettes per day, and *dur* the duration of smoking in years. The mean absolute risk of death due to lung cancer for continuing smokers is then given by $R_{S}=R_{NS}+ER_{S}.$

For former smokers, the absolute risk of death due to lung cancer was modeled as:

$$R_{FS}=R_{NS}+f(qt\_yrs,qt\_age)\times ER_{S},$$

where *R_NS_* is the plug-in absolute risk function for never smokers of the same age; *ER_S_* is a plug-in excess risk function for continuing smokers of the same age, CPD, and duration; and *f* is a function of the decrease in excess risk for former smokers, which was assumed to be a non-increasing function of time in years since cessation (*qt_yrs*) and age in years at cessation (*qt_age*). A negative exponential function with a lag of two years was used to model the function *f*: $f(qt\_yrs,qt\_age)=\exp[-(0.274-0.00279\times qt\_age)\times(qt\_yrs-2)].$

To compute the 6-year probability to die due to lung cancer in the follow-up given that the person hasn’t died from cancer by the age at the entry (say *t_a_*), we assume that the smoking patterns at age at entry remain the same for the next 6 years of follow-up. The conditional 6-year probability is computed by

$$\Pr(T\leq t_{a}+6|T>t_{a})=\frac{\Pr(t_{a}<T\leq t_{a}+6)}{\Pr(T>t_{a})}=1-\frac{S(t_{a}+6)}{S(t_{a})},$$

where S(t) is the survival function and computed by $\exp[-\int_{0}^{t} R_{i}(u)du]$, *i=NS, S, FS* corresponding to never, continuing, former smokers.

**References**

1. Bach PB, Kattan MW, Thornquist MD, Kris MG, Tate RC, Barnett MJ, et al. Variations in Lung Cancer Risk Among Smokers. J Natl Cancer Inst. 2003;95(6):470-8. doi: 10.1093/jnci/95.6.470.

2. Cronin KA, Gail MH, Zou Z, Bach PB, Virtamo J, Albanes D. Validation of a Model of Lung Cancer Risk Prediction Among Smokers. J Natl Cancer Inst. 2006;98(9):637-40. doi: 10.1093/jnci/djj163.

3. Cassidy A, Myles JP, van Tongeren M, Page RD, Liloglou T, Duffy SW, et al. The LLP risk model: an individual risk prediction model for lung cancer. Br J Cancer. 2007;98(2):270-6.

4. D'Amelio AM, Jr., Cassidy A, Asomaning K, Raji OY, Duffy SW, Field JK, et al. Comparison of discriminatory power and accuracy of three lung cancer risk models. Br J Cancer. 2010;103(3):423-9.

5. Raji OY, Duffy SW, Agbaje OF, Baker SG, Christiani DC, Cassidy A, et al. Predictive Accuracy of the Liverpool Lung Project Risk Model for Stratifying Patients for Computed Tomography Screening for Lung CancerA Case–Control and Cohort Validation Study. Ann Intern Med. 2012;157(4):242-50.

6. Tammemagi MC, Katki HA, Hocking WG, Church TR, Caporaso N, Kvale PA, et al. Selection criteria for lung-cancer screening. N Engl J Med. 2013;368(8):728-36. PubMed PMID: 23425165.

7. Meza R, Hazelton WD, Colditz GA, Moolgavkar SH. Analysis of lung cancer incidence in the nurses’ health and the health professionals’ follow-up studies using a multistage carcinogenesis model. Cancer Causes Control. 2008;19(3):317-28. doi: 10.1007/s10552-007-9094-5.

8. Hazelton WD, Jeon J, Meza R, Moolgavkar SH. Chapter 8: The FHCRC Lung Cancer Model. Risk Anal. 2012;32:S99-S116. doi: 10.1111/j.1539-6924.2011.01681.x.

9. Hazelton WD, Clements MS, Moolgavkar SH. Multistage Carcinogenesis and Lung Cancer Mortality in Three Cohorts. Cancer Epidemiol Biomarkers Prev. 2005;14(5):1171-81. doi: 10.1158/1055-9965.epi-04-0756.

10. Heidenreich WF, Luebeck EG, Moolgavkar SH. Some Properties of the Hazard Function of the Two-Mutation Clonal Expansion Model. Risk Anal. 1997;17(3):391-9. doi: 10.1111/j.1539-6924.1997.tb00878.x.

11. Knoke JD, Burns DM, Thun MJ. The change in excess risk of lung cancer attributable to smoking following smoking cessation: an examination of different analytic approaches using CPS-I data. Cancer Causes Control. 2008;19(2):207-19. doi: 10.1007/s10552-007-9086-5.
