# Supplementary material for: Risk prediction models for selection of lung cancer screening candidates: A retrospective validation study
Source: PLoS Med. 2017 Apr 4;14(4):e1002277. doi: 10.1371/journal.pmed.1002277 (PMC5380315; doi:10.1371/journal.pmed.1002277)
Supplement: S2 Appendix — (DOCX) [file pmed.1002277.s002.docx]

**S2 Appendix: National Lung Screening Trial and Prostate, Lung, Colorectal and Ovarian Cancer Screening Trial participant characteristics**

**Table A: Characteristics of National Lung Screening Trial (NLST) and Prostate, Lung, Colorectal and Ovarian cancer screening trial (PLCO) participants according to 6-year lung cancer mortality**

|  | **NLST (computed tomography-arm)** | |  | **NLST (chest radiography arm)** | |  | **PLCO (chest radiography arm)** | |  | **PLCO (control arm)** | |  |
| --- | --- | --- | --- | --- | --- | --- | --- | --- | --- | --- | --- | --- |
| **Characteristics (N,%)** | **No lung cancer mortality** 26,325 (98.51%) | **Lung cancer mortality** 397 (1.49%) | **P-value** | **No lung cancer mortality** 26,243 (98.18%) | **Lung cancer mortality** 487 (1.82%) | **P-value** | **No lung cancer mortality** 40,155 (98.90%) | **Lung cancer mortality** 445 (1.10%) | **P-value** | **No lung cancer mortality** 39,602 (98.83%) | **Lung cancer mortality** 470 (1.17%) | **P-value** |
| **Age (median, IQR)** | 60 (57-65) | 64 (60-68) | <0.0001 | 60 (57-65) | 63 (59.5-68) | <0.0001 | 62 (58-66) | 66 (61-69) | <0.0001 | 62 (58-66) | 65 (61-69) | <0.0001 |
| **Missing (N,%)** | 0 (0%) | 0 (0%) |  | 0 (0%) | 0 (0%) |  | 0 (0%) | 0 (0%) |  | 0 (0%) | 0 (0%) |  |
| **Gender** |  |  |  |  |  |  |  |  |  |  |  |  |
| Male | 15,503 (58.89%) | 266 (67.00%) | 0.0012 | 15,465 (58.93%) | 296 (60.78%) | 0.4295 | 23,408 (58.29%) | 295 (66.29%) | 0.0007 | 22,906 (57.84%) | 307 (65.32%) | 0.0012 |
| Female | 10,822 (41.11%) | 131 (33.00%) |  | 10,778 (41.07%) | 191 (39.22%) |  | 16,747 (41.71%) | 150 (33.71%) |  | 16,696 (42.16%) | 163 (34.68%) |  |
| **Missing (N,%)** | 0 (0%) | 0 (0%) |  | 0 (0%) | 0 (0%) |  | 0 (0%) | 0 (0%) |  | 0 (0%) | 0 (0%) |  |
| **Hispanic ethnicity** |  |  |  |  |  |  |  |  |  |  |  |  |
| No | 25,689 (97.58%) | 389 (97.98%) | 0.0530 | 25,565 (97.42%) | 474 (97.33%) | 0.0725 | 38,418 (95.67%) | 429 (96.40%) | 0.8704 | 37,830 (95.53%) | 457 (97.23%) | 0.1113 |
| Yes | 477 (1.81%) | 2 (0.50%) |  | 453 (1.73%) | 3 (0.62%) |  | 874 (2.18%) | 10 (2.25%) |  | 877 (2.21%) | 5 (1.06%) |  |
| **Missing (N,%)** | 159 (0.60%) | 6 (1.51%) |  | 225 (0.86%) | 10 (2.05%) |  | 863 (2.15%) | 6 (1.35%) |  | 895 (2.26%) | 8 (1.70%) |  |
| **Race or ethnic group*** |  |  |  |  |  |  |  |  |  |  |  |  |
| White | 23,593 (89.62%) | 359 (90.43%) | 0.0489 | 23,514 (89.60%) | 435 (89.32%) | 0.2235 | 35,449 (88.29%) | 375 (84.27%) | 0.0025 | 35,003 (88.39%) | 414 (88.09%) | 0.0440 |
| Black | 1,170 (4.44%) | 17 (4.28%) |  | 1,145 (4.36%) | 29 (5.95%) |  | 2,279 (5.68%) | 46 (10.34%) |  | 2,214 (5.59%) | 40 (8.51%) |  |
| Hispanic | 343 (1.30%) | 1 (0.25%) |  | 316 (1.20%) | 2 (0.41%) |  | 812 (2.02%) | 10 (2.25%) |  | 810 (2.05%) | 5 (1.06%) |  |
| Asian | 552 (2.10%) | 7 (1.76%) |  | 530 (2.02%) | 6 (1.23%) |  | 1239 (3.09%) | 9 (2.02%) |  | 1202 (3.04%) | 10 (2.13%) |  |
| Native Hawaiian or Pacific Islander | 88 (0.33%) | 3 (0.76%) |  | 101 (0.38%) | 1 (0.21%) |  | 223 (0.56%) | 4 (0.90%) |  | 244 (0.62%) | 1 (0.21%) |  |
| American Indian or Alaskan Native | 88 (0.33%) | 4 (1.01%) |  | 97 (0.37%) | 1 (0.21%) |  | 130 (0.32%) | 1 (0.22%) |  | 109 (0.28%) | 0 (0%) |  |
| **Missing (N,%)** | 491 (1.87%) | 6 (1.51%) |  | 540 (2.06%) | 13 (2.67%) |  | 23 (0.06%) | 0 (0.0%) |  | 20 (0.05%) | 0 (0.0%) |  |
| **Education** |  |  |  |  |  |  |  |  |  |  |  |  |
| Less than high school grad | 1,597 (6.07%) | 44 (11.08%) | <0.0001 | 1,563 (5.96%) | 45 (9.24%) | <0.0001 | 3,470 (8.64%) | 77 (17.30%) | <0.0001 | 3,445 (8.70%) | 69 (14.68%) | <0.0001 |
| High school grad | 6,161 (23.40%) | 112 (28.21%) |  | 6,299 (24.00%) | 139 (28.54%) |  | 8,860 (22.06%) | 114 (25.62%) |  | 8,704 (21.98%) | 140 (29.79%) |  |
| Post high school training | 3,678 (13.97%) | 55 (13.85%) |  | 3,620 (13.79%) | 81 (16.63%) |  | 5,378 (13.39%) | 55 (12.36%) |  | 5,433 (13.72%) | 58 (12.34%) |  |
| Some college | 6,099 (23.17%) | 90 (22.67%) |  | 5,982 (22.79%) | 106 (21.77%) |  | 9,336 (23.25%) | 98 (22.02%) |  | 9,223 (23.29%) | 97 (20.64%) |  |
| College grad | 4,455 (16.92%) | 50 (12.59%) |  | 4,385 (16.71%) | 56 (11.50%) |  | 6,667 (16.60%) | 56 (12.58%) |  | 6,421 (16.21%) | 58 (12.34%) |  |
| Postgraduate/professional | 3,749 (14.24%) | 31 (7.81%) |  | 3,771 (14.37%) | 49 (10.06%) |  | 6,384 (15.90%) | 45 (10.11%) |  | 6,232 (15.74%) | 45 (9.57%) |  |
| **Missing (N,%)** | 586 (2.23%) | 15 (3.78%) |  | 623 (2.37%) | 11 (2.26%) |  | 60 (0.15%) | 0 (0.0% |  | 144 (0.36%) | 3 (0.64%) |  |
| **BMI (median, IQR)** | 27.32 (24.46-30.69) | 26.22 (24.00-29.63 | 0.0002 | 27.38 (24.46-30.61) | 25.90 (23.45-29.29) | <0.0001 | 26.68 (24.19-29.93) | 26.16 (23.32-28.95) | 0.0002 | 26,68 (24.18-29.89) | 25.88 (23.44-28.94) | <0.0001 |
| **Missing (N,%)** | 154 (0.58%) | 5 (1.26%) |  | 206 (0.78%) | 7 (1.44%) |  | 401 (1.00%) | 3 (0.67%) |  | 750 (1.89%) | 7 (1.49%) |  |
| **COPD** |  |  |  |  |  |  |  |  |  |  |  |  |
| No | 21,768 (82.69%) | 280 (70.53%) | <0.0001 | 21,709 (82.72%) | 369 (75.77%) | <0.0001 | 36,635 (91.23%) | 348 (78.20%) | <0.0001 | 36,095 (91.14%) | 371 (78.94%) | <0.0001 |
| Yes | 4,557 (17.31%) | 117 (29.47%) |  | 4,534 (17.28%) | 118 (24.23%) |  | 3,520 (8.77%) | 97 (21.80%) |  | 3,507 (8.86%) | 99 (21.06%) |  |
| **Missing (N,%)** | 0 (0%) | 0 (0%) |  | 0 (0%) | 0 (0%) |  | 0 (0%) | 0 (0%) |  | 0 (0%) | 0 (0%) | 0 (0%) |
| **Emphysema** |  |  |  |  |  |  |  |  |  |  |  |  |
| No | 24,217 (91.99%) | 322 (81.11%) | <0.0001 | 24,088 (91.79%) | 412 (84.60%) | <0.0001 | 38,287 (95.35%) | 379 (85.17%) | <0.0001 | 37,607 (94.96%) | 396 (84.26%) | <0.0001 |
| Yes | 1,985 (7.54%) | 71 (17.88%) |  | 1,967 (7.50%) | 70 (14.37%) |  | 1,681 (4.19%) | 65 (14.61%) |  | 1,641 (4.14%) | 70 (14.89%) |  |
| **Missing (N,%)** | 123 (0.47%) | 4 (1.01%) |  | 188 (0.72%) | 5 (1.03%) |  | 187 (0.47%) | 1 (0.22%) |  | 354 (0.89%_ | 4 (0.85%) |  |
| **Personal history of cancer** |  |  |  |  |  |  |  |  |  |  |  |  |
| No | 25,183 (95.66%) | 361 (90.93%) | 0.0003 | 24,929 (94.99%) | 458 (94.05%) | 0.6584 | 38,321 (95.43%) | 421 (94.61%) | 0.4229 | 37,751 (95.33%) | 434 (92.34%) | 0.0042 |
| Yes | 1,064 (4.04%) | 32 (8.06%) |  | 1,188 (4.53%) | 24 (4.53%) |  | 1,834 (4.57%) | 24 (5.39%) |  | 1,851 (4.67%) | 36 (7.66%) |  |
| **Missing (N,%)** | 78 (0.30%) | 4 (1.01%) |  | 126 (0.48%) | 5 (1.03%) |  | 0 (0%) | 0 (0%) |  | 0 (0%) | 0 (0%) |  |
| **Family history of lung cancer** |  |  |  |  |  |  |  |  |  |  |  |  |
| No | 20,199 (76.73%) | 288 (72.54%) | 0.1224 | 20,103 (76.60%) | 349 (71.66%) | 0.0142 | 33,952 (84.55%) | 331 (74.39%) | <0.0001 | 33,658 (84.99%) | 368 (78.30%) | 0.0006 |
| Yes | 5,717 (21.72%) | 98 (24.69%) |  | 5,678 (21.64%) | 128 (26.28%) |  | 4,568 (11.38%) | 85 (19.10%) |  | 4,467 (11.28%) | 77 (16.38%) |  |
| **Missing (N,%)** | 409 (1.55%) | 11 (2.77%) |  | 462 (1.76%) | 10 (2.05%) |  | 1,635 (4.07%) | 29 (6.52%) |  | 1,477 (3.73%) | 25 (5.32%) |  |
| **Personal history of pneumonia** |  |  |  |  |  |  |  |  |  |  |  |  |
| No | 20,402 (77.50%) | 284 (71.54%) | 0.0143 | 20,319 (77.43%) | 361 (74.13%) | 0.1209 | **Not measured** | **Not measured** | **Not measured** | **Not measured** | **Not measured** | **Not measured** |
| Yes | 5,822 (22.12%) | 108 (27.20%) |  | 5,758 (21.94%) | 121 (24.85%) |  | **Not measured** | **Not measured** |  | **Not measured** | **Not measured** |  |
| **Missing (N,%)** | 101 (0.38%) | 5 (1.26%) |  | 166 (0.63%) | 5 (1.03%) |  | **Not measured** | **Not measured** |  | **Not measured** | **Not measured** |  |
| **Smoking status** |  |  |  |  |  |  |  |  |  |  |  |  |
| Current smoker | 13,794 (52.40%) | 144 (36.27%) | <0.0001 | 13,736 (52.34%) | 162 (33.26%) | <0.0001 | 32,285 (80.40%) | 239 (53.71%) | <0.0001 | 31,824 (80.36%) | 269 (57.23%) | <0.0001 |
| Former smoker | 12,531 (47.60%) | 253 (63.73%) |  | 12,507 (47.66%) | 325 (66.74%) |  | 7,870 (19.60%) | 206 (46.29%) |  | 7,778 (19.64%) | 201 (42.77%) |  |
| **Missing (N,%)** | 0 (0%) | 0 (0%) |  | 0 (0%) | 0 (0%) |  | 0 (0%) | 0 (0%) |  | 0 (0%) | 0 (0%) |  |
| **Smoking duration (years) (median, IQR)** | 40 (35-45) | 45 (40-50) | <0.0001 | 40 (35-45) | 44 (40-49) | <0.0001 | 28 (16-39) | 43 (37-48) | <0.0001 | 28 (16-39) | 42 (34-47) | <0.0001 |
| **Missing (N,%)** | 0 (0%) | 0 (0%) |  | 0 (0%) | 0 (0%) |  | 767 (1.91%) | 9 (2.02%) |  | 881 (2.22%) | 13 (2.77%) |  |
| **Smoking intensity (cigarettes per day) (median, IQR)** | 25 (20-35) | 30 (20-40) | 0.0006 | 25 (20-32) | 30 (20-40) | 0.0029 | 20 (10-30) | 30 (20-40) | <0.0001 | 20 (10-30) | 30 (20-40) | <0.0001 |
| **Missing (N,%)** | 0 (0%) | 0 (0%) |  | 0 (0%) | 0 (0%) |  | 81 (0.20%) | 1 (0.22%) |  | 113 (0.29%) | 1 (0.21%) |  |
| **Pack-years of smoking (median, IQR)** | 48 (39-66) | 58 (46-86) | <0.0001 | 48 (39-66) | 57 (45.5-80) | <0.0001 | 28.5 (14-48) | 52 (40-75) | <0.0001 | 29 (14-49.5) | 53 (40-75) | <0.0001 |
| **Missing (N,%)** | 0 (0%) | 0 (0%) |  | 0 (0%) | 0 (0%) |  | 830 (2.1%) | 10 (2.2%) |  | 962 (2.4%) | 14 (3.0%) |  |
| **Smoking quit time (years) (median, IQR)** | 7 (3-11) | 5 (2-10) | 0.0002 | 7 (3-11) | 6 (2-11) | 0.2844 | 20 (10-30) | 9 (3-18.75) | <0.0001 | 20 (10-30) | 10 (4-19) | <0.0001 |
| **Missing (N,%)** | 223 (0.8%) | 1 (0.3%) |  | 220 (0.8%) | 4 (0.8%) |  | 561 (1.4%) | 5 (1.1%) |  | 680 (1.7%) | 4 (0.9%) |  |
