# Supplementary material for: Risk prediction models for selection of lung cancer screening candidates: A retrospective validation study
Source: PLoS Med. 2017 Apr 4;14(4):e1002277. doi: 10.1371/journal.pmed.1002277 (PMC5380315; doi:10.1371/journal.pmed.1002277)
Supplement: S3 Appendix — (DOCX) [file pmed.1002277.s003.docx]

**S3 Appendix: Overview of National Lung Screening Trial and Prostate, Lung, Colorectal and Ovarian Cancer Screening Trial participants with complete information**

**Table A: Proportions of National Lung Screening Trial (NLST) and Prostate, Lung, Colorectal and Ovarian Cancer Screening Trial (PLCO) participants with complete information for all considered risk factors**

| **6-year lung cancer incidence** | **All participants** | **All participants with complete information** |  | **Participants who were not diagnosed with lung cancer** | **Participants who were not diagnosed with lung cancer with complete information** |  | **Participants who were diagnosed with lung cancer** | **Participants who were diagnosed with lung cancer with complete information** |
| --- | --- | --- | --- | --- | --- | --- | --- | --- |
| **NLST-CT** | 26,722 | 25,032 (93.68%) |  | 25,692 | 24,076 (93.71%) |  | 1,030 | 956 (92.82%) |
| **NLST-chest radiography** | 26,730 | 24,998 (93.52%) |  | 25,835 | 24,155 (93.50%) |  | 895 | 843 (94.19%) |
| **PLCO- chest radiography** | 40,600 | 37,657 (92.75%) |  | 39,846 | 36,974 (92.79%) |  | 754 | 683 (90.58%) |
| **PLCO-Control** | 40,072 | 36,951 (92.21%) |  | 39,363 | 36,313 (92.25%) |  | 709 | 638 (89.99%) |
| **6-year lung cancer mortality** | **All participants** | **All participants with complete information** |  | **Participants who were not diagnosed with lung cancer** | **Participants who were not diagnosed with lung cancer with complete information** |  | **Participants who were diagnosed with lung cancer** | **Participants who were diagnosed with lung cancer with complete information** |
| **NLST-CT** | 26,722 | 25,032 (93.68%) |  | 26,325 | 24,661 (93.68%) |  | 397 | 371 (93.45%) |
| **NLST- chest radiography** | 26,730 | 24,998 (93.52%) |  | 26,243 | 24,540 (93.51%) |  | 487 | 458 (94.05%) |
| **PLCO- chest radiography** | 40,600 | 37,657 (92.75%) |  | 40,155 | 37,254 (92.78%) |  | 445 | 403 (90.56%) |
| **PLCO-Control** | 40,072 | 36,951 (92.21%) |  | 39,602 | 36,527 (92.24%) |  | 470 | 424 (90.21%) |
