# Supplementary material for: Risk prediction models for selection of lung cancer screening candidates: A retrospective validation study
Source: PLoS Med. 2017 Apr 4;14(4):e1002277. doi: 10.1371/journal.pmed.1002277 (PMC5380315; doi:10.1371/journal.pmed.1002277)
Supplement: S4 Appendix — (DOCX) [file pmed.1002277.s004.docx]

**S4 Appendix: Calibration aspects of the evaluated lung cancer risk prediction models for 6-year lung cancer incidence and mortality**

**Figure A: Calibration plots for the Bach model for 6-year lung cancer incidence in all datasets**

**NLST CT arm NLST CXR arm**


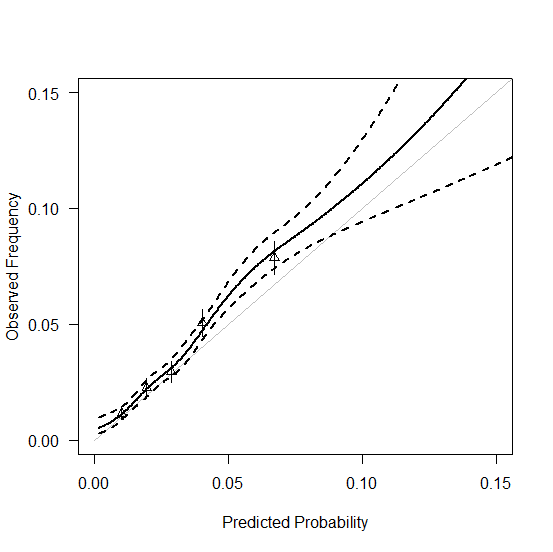

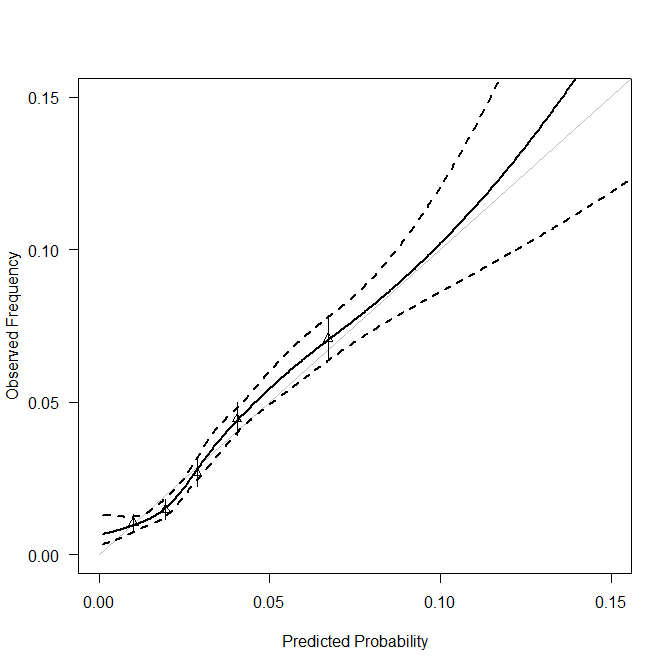


**PLCO CXR arm PLCO Control arm**

**
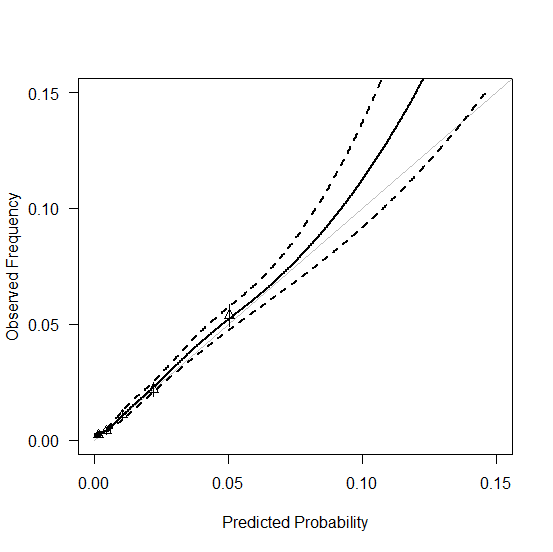

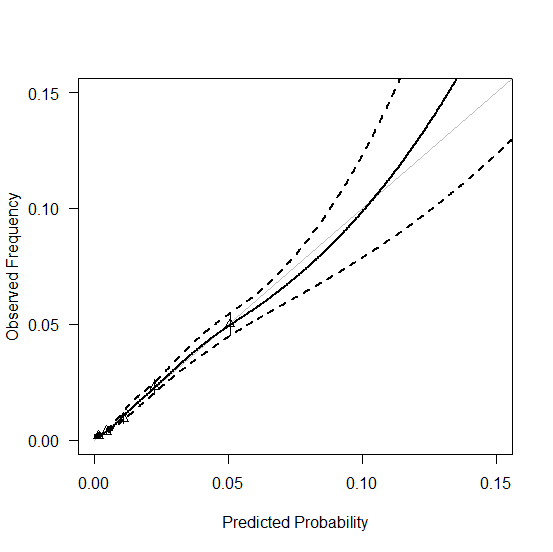
**

**Figure B: Calibration plots for the Bach model for 6-year lung cancer mortality in all datasets**

**NLST CT arm NLST CXR arm**

**
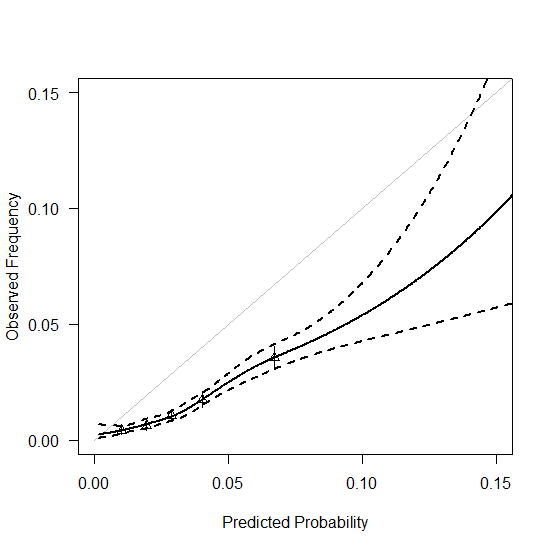
**
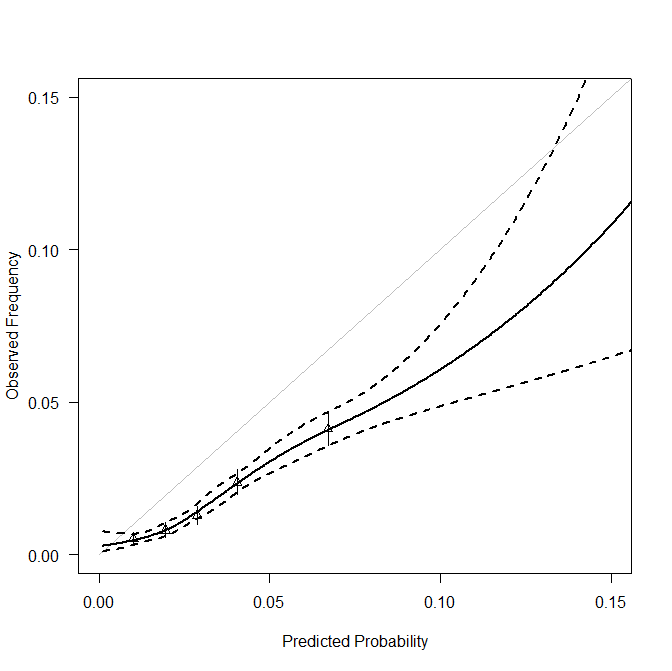


**PLCO CXR arm PLCO Control arm**

**
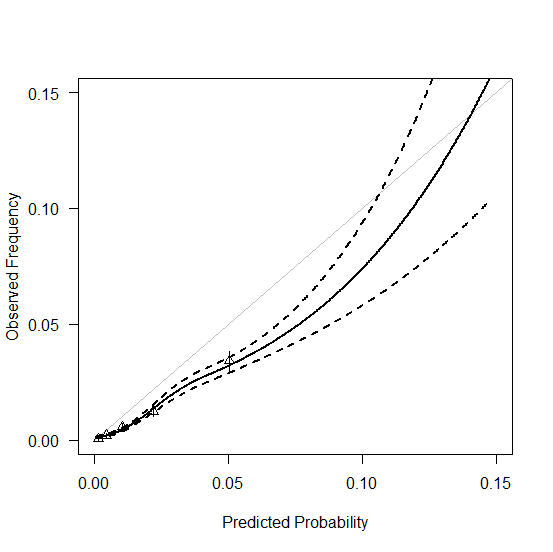

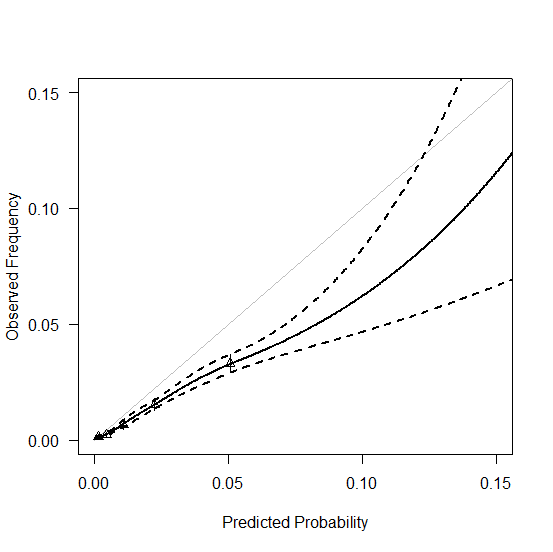
**

**Figure C: Calibration plots for the LLP model for 6-year lung cancer incidence in all datasets**

**NLST CT arm NLST CXR arm**

**
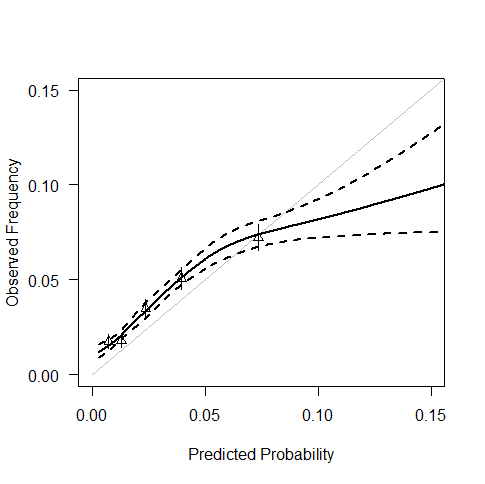
**
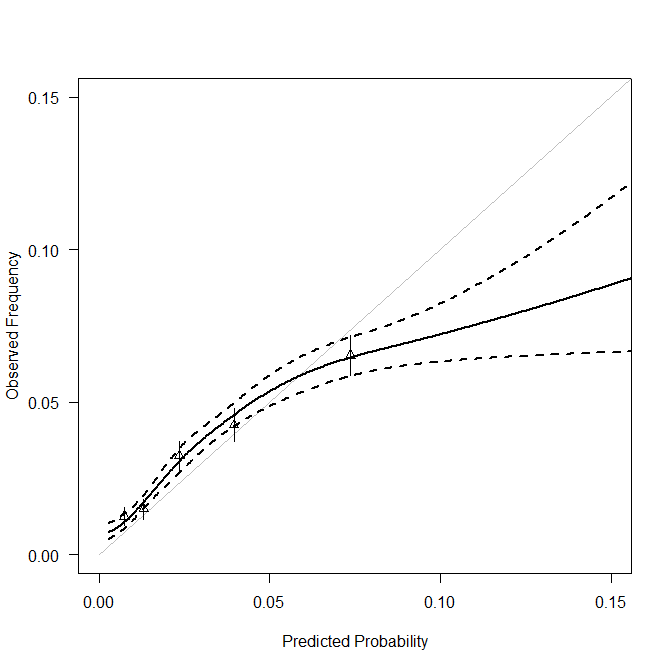


**PLCO CXR arm PLCO Control arm**

**
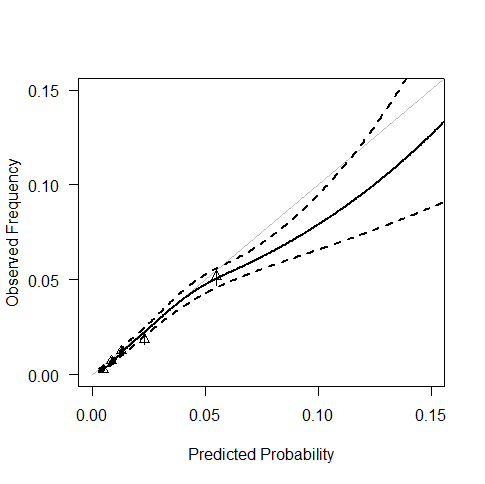

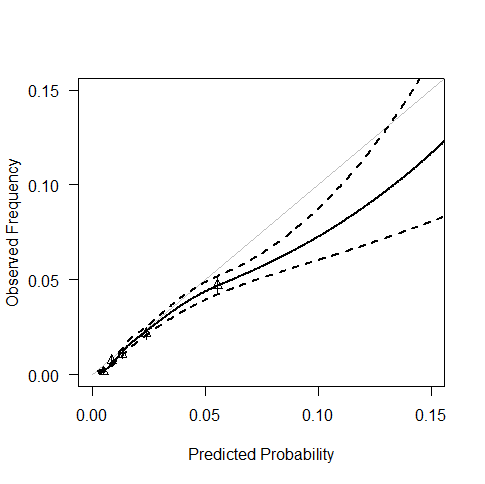
**

**Figure D: Calibration plots for the LLP model for 6-year lung cancer mortality in all datasets**

**NLST CT arm NLST CXR arm**

**
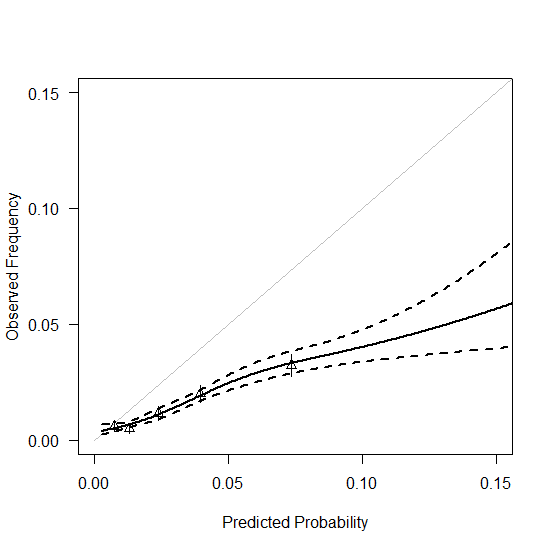
**
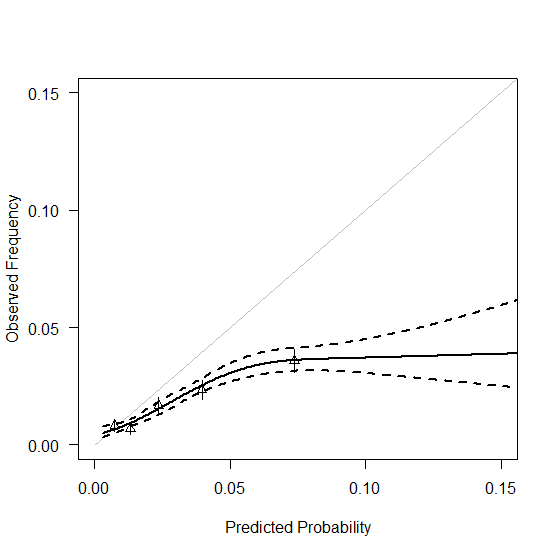


**PLCO CXR arm PLCO Control arm**

**
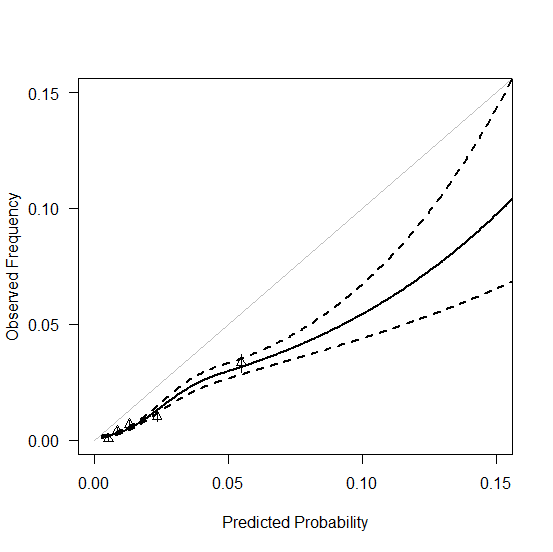

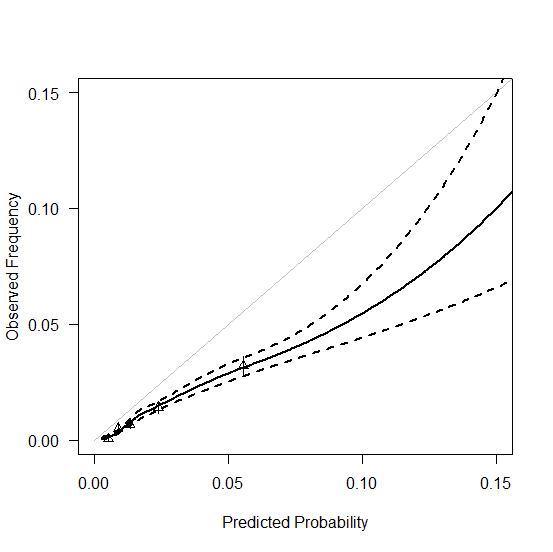
**

**Figure E: Calibration plots for the simplified LLP model for 6-year lung cancer incidence in all datasets**

**NLST CT arm NLST CXR arm**

**
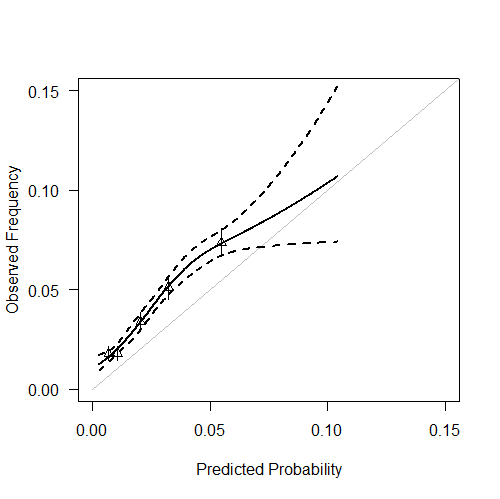
**
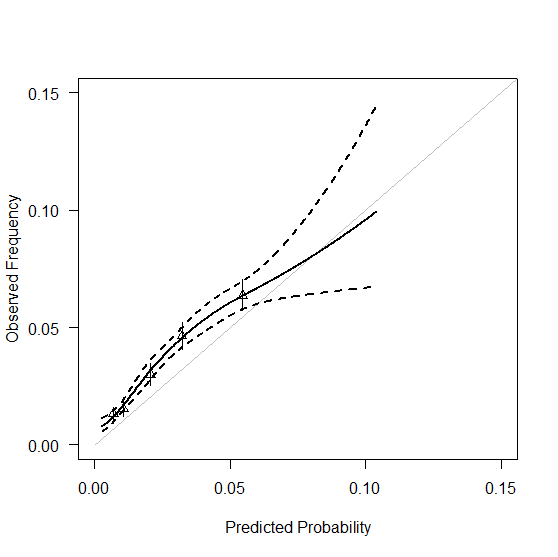


**PLCO CXR arm PLCO Control arm**

**
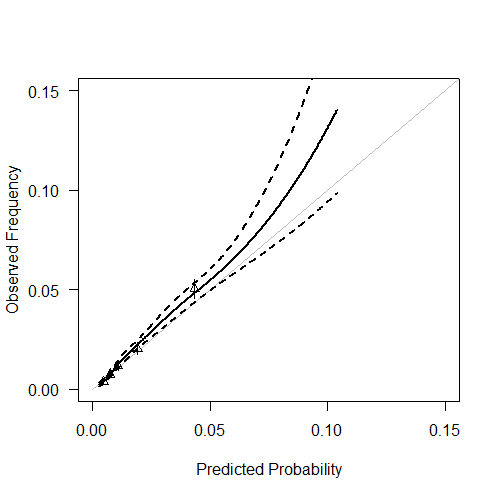

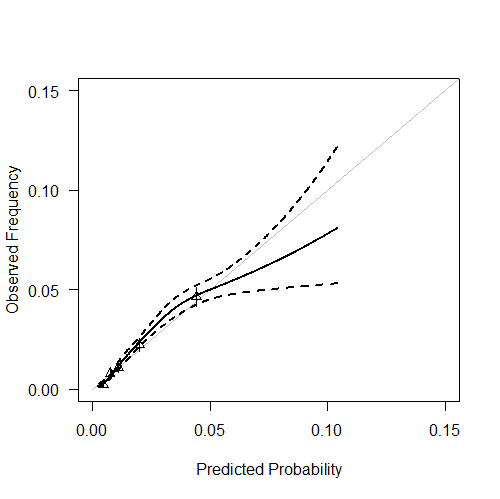
**

**Figure F: Calibration plots for the simplified LLP model for 6-year lung cancer mortality in all datasets**

**NLST CT arm NLST CXR arm**

**
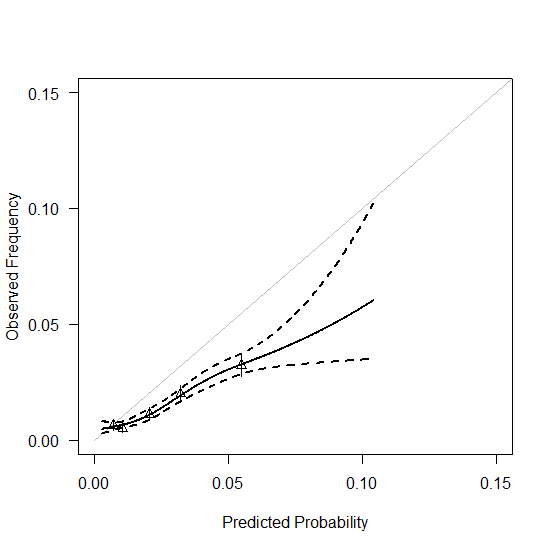
**
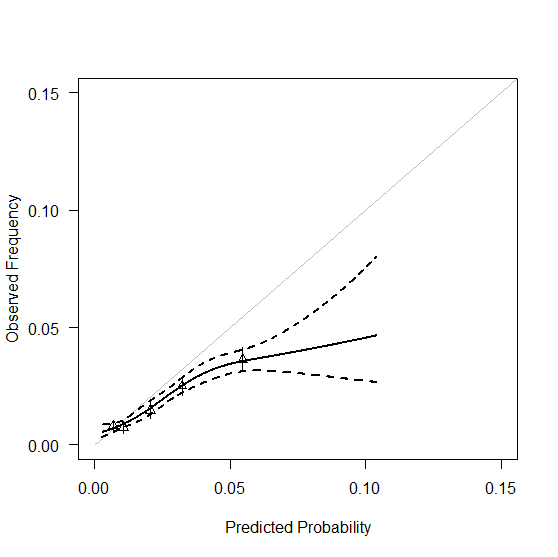


**PLCO CXR arm PLCO Control arm**

**
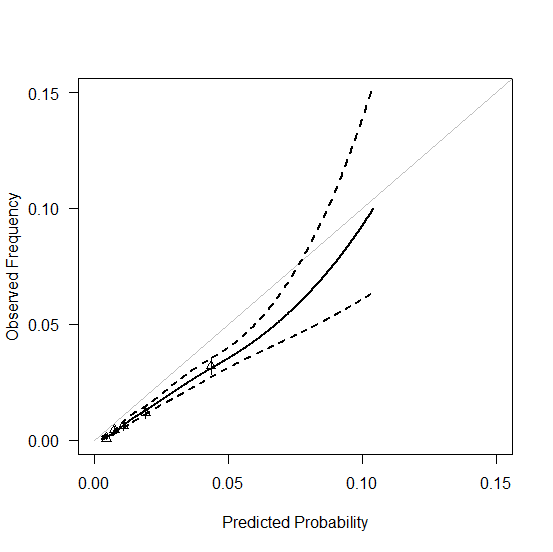

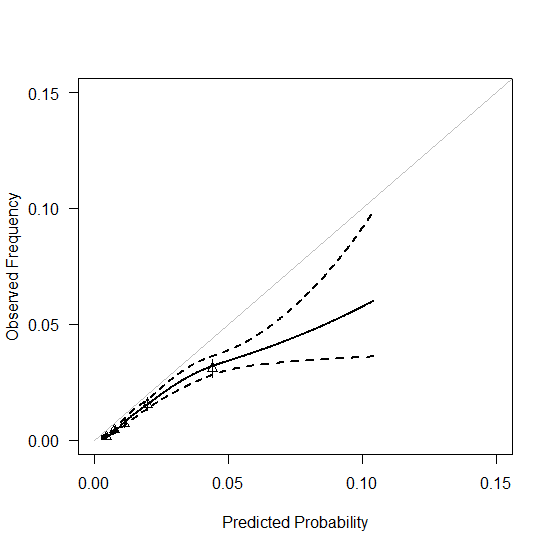
**

**Figure G: Calibration plots for the PLCOm2012 model for 6-year lung cancer incidence in all datasets**

**NLST CT arm NLST CXR arm**

**
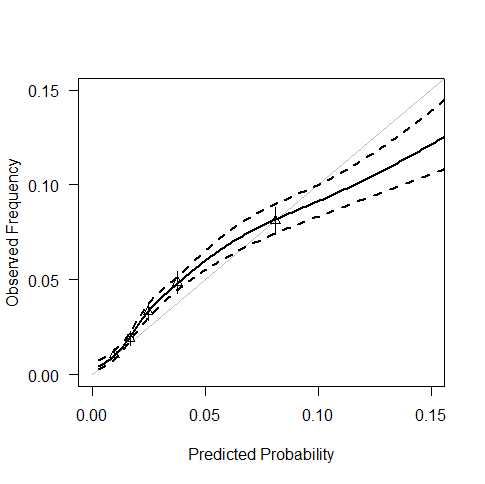
**
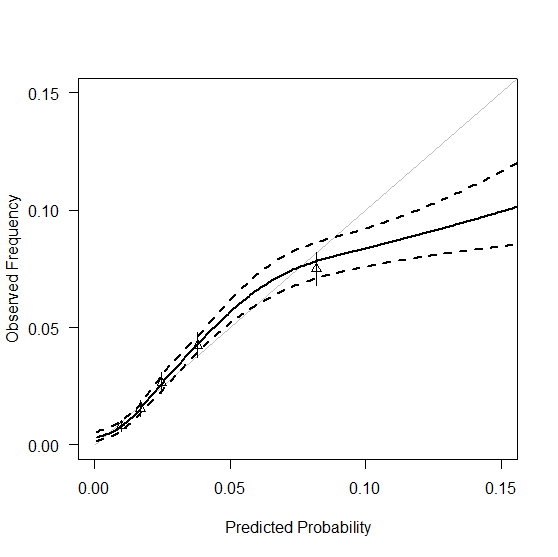


**PLCO CXR arm PLCO Control arm
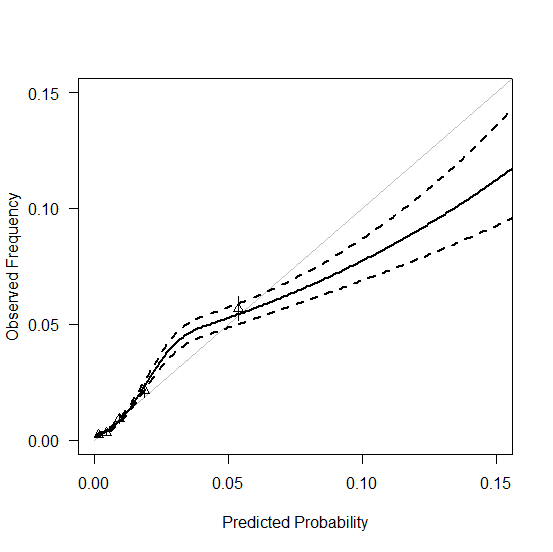

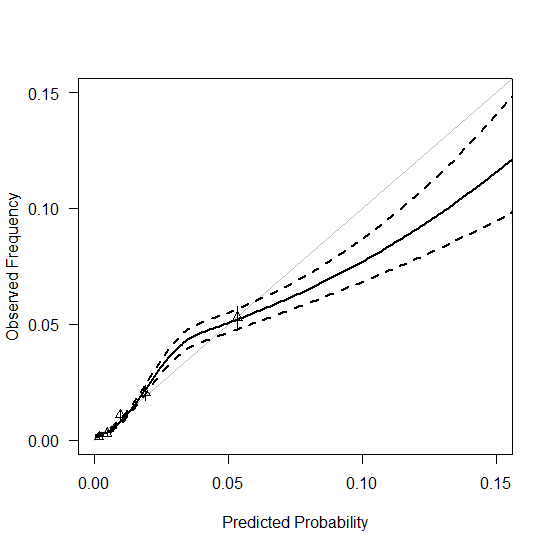
**

**Figure H: Calibration plots for the PLCOm2012 model for 6-year lung cancer mortality in all datasets**

**NLST CT arm NLST CXR arm**

**
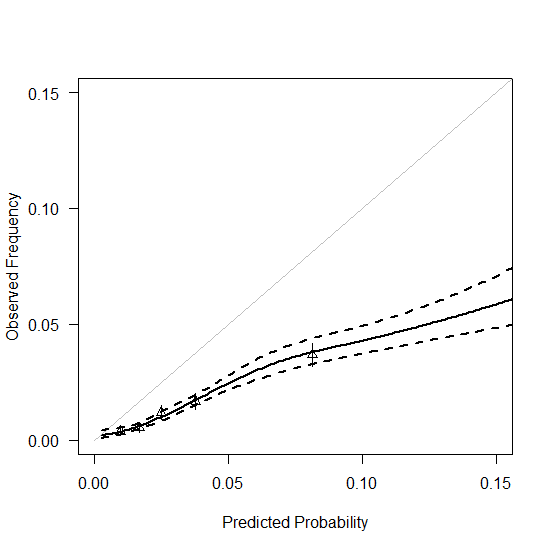
**
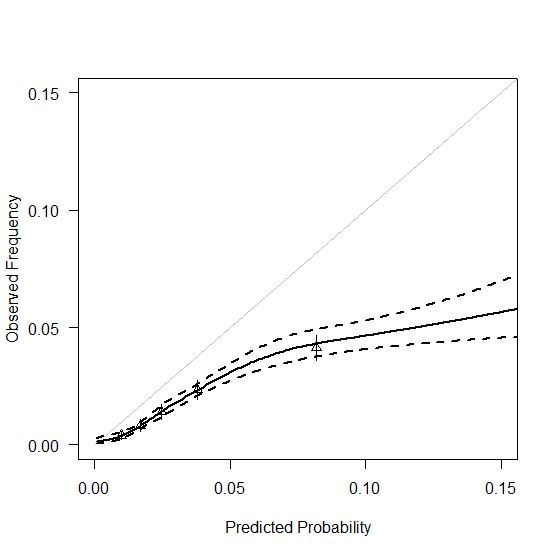


**PLCO CXR arm PLCO Control arm**

**
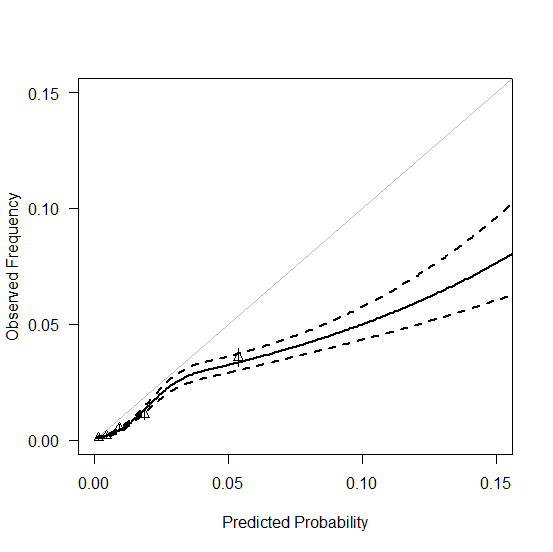

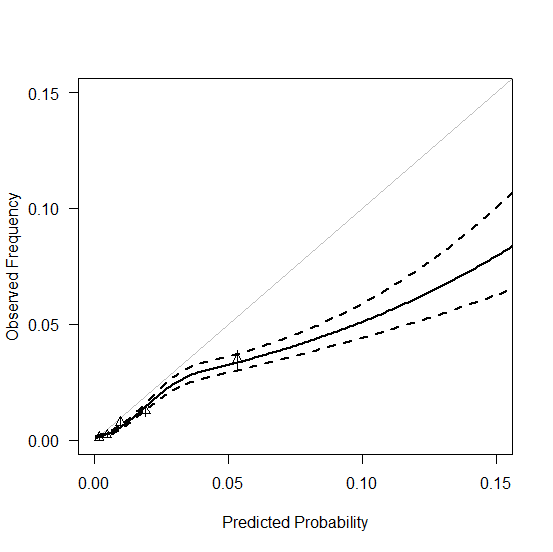
**

**Figure I: Calibration plots for the simplified PLCOm2012 model for 6-year lung cancer incidence in all datasets**

**NLST CT arm NLST CXR arm**

**
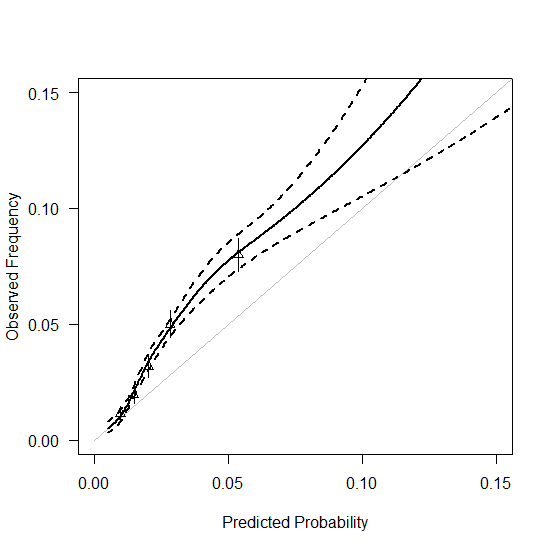
**
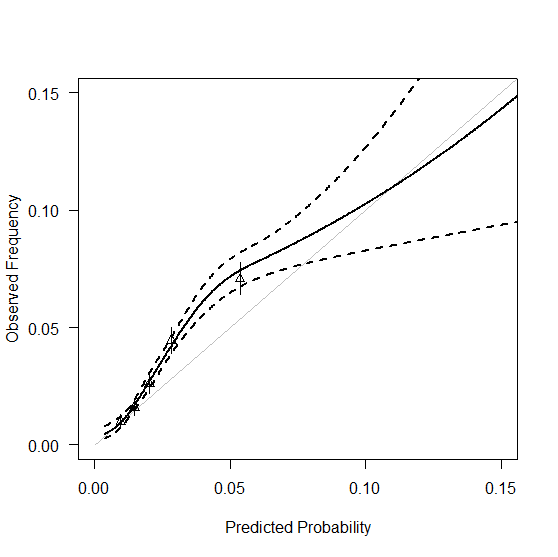


**PLCO CXR arm PLCO Control arm**

**
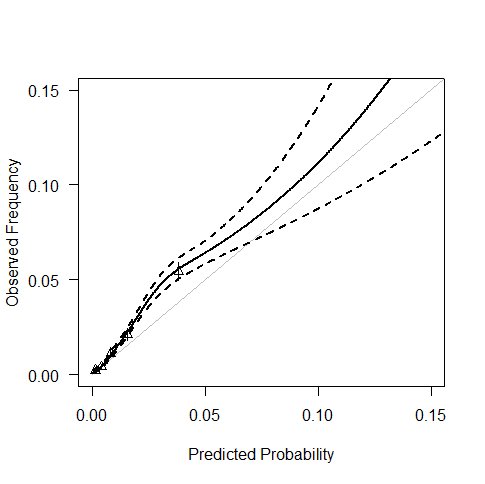

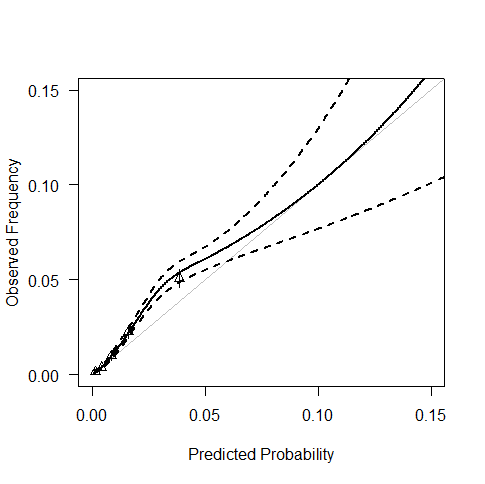
**

**Figure J: Calibration plots for the simplified PLCOm2012 model for 6-year lung cancer mortality in all datasets**

**NLST CT arm NLST CXR arm**

**
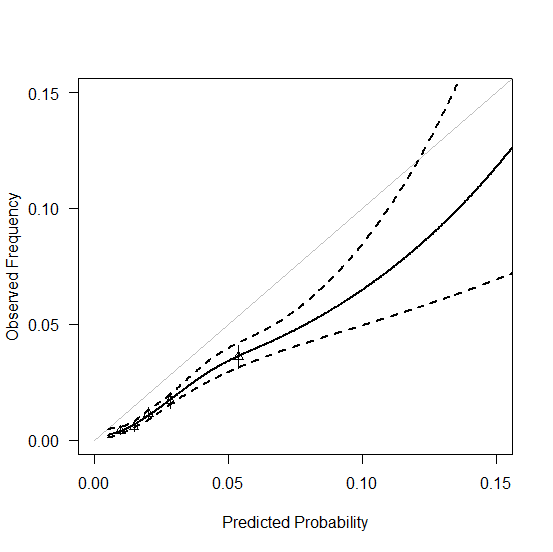
**
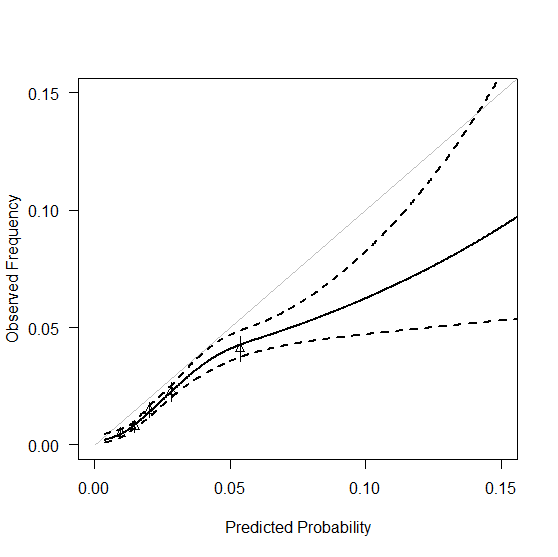


**PLCO CXR arm PLCO Control arm**

**
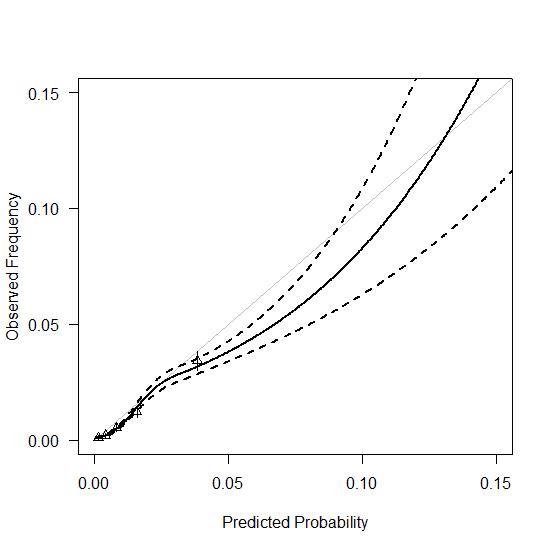

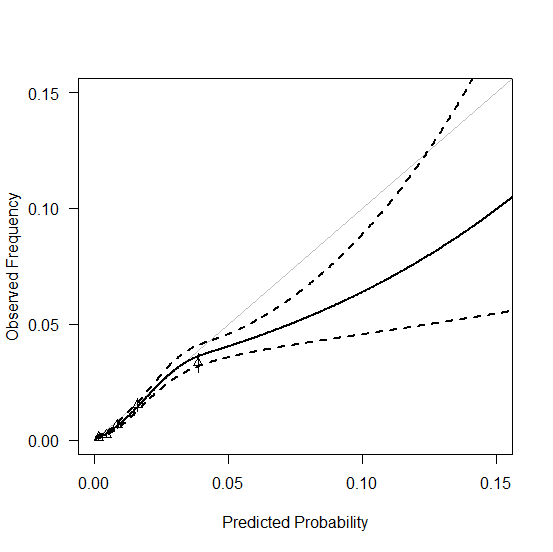
**

**Figure K: Calibration plots for the TSCE incidence model for 6-year lung cancer incidence in all datasets**

**NLST CT arm NLST CXR arm**

**
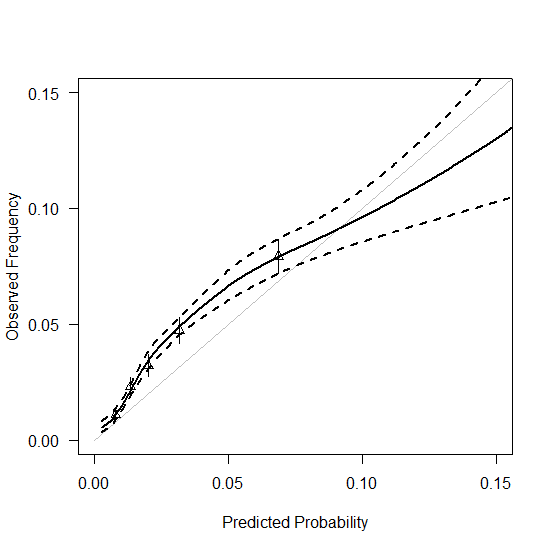
**
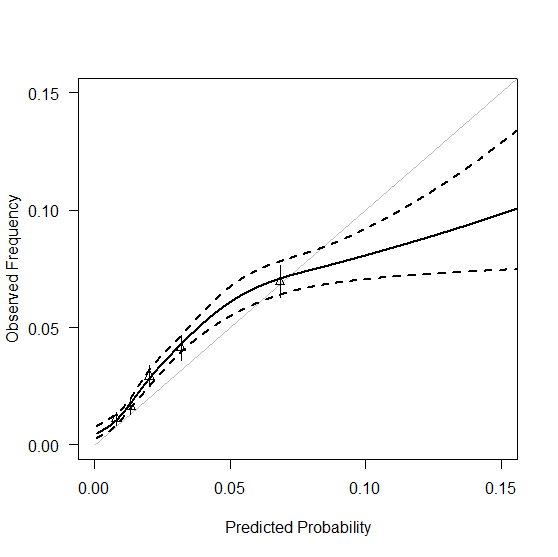


**PLCO CXR arm PLCO Control arm**


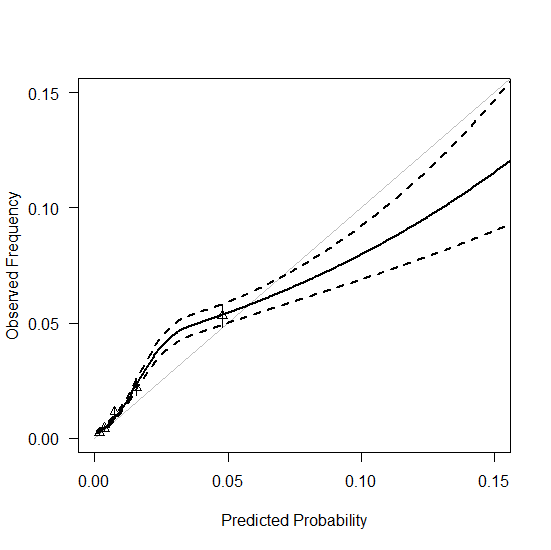

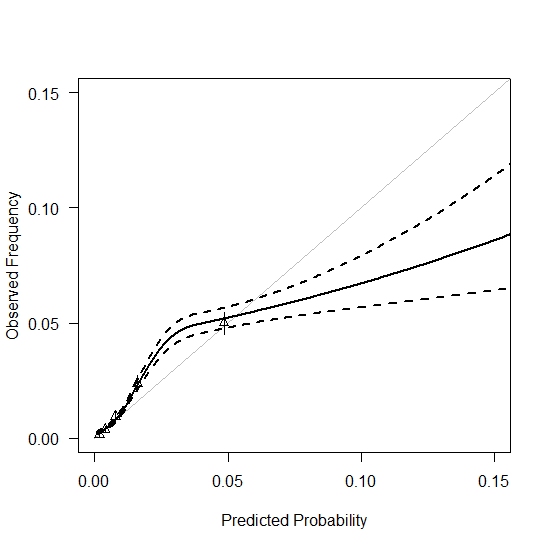


**Figure L: Calibration plots for the TSCE incidence model for 6-year lung cancer mortality in all datasets**

**NLST CT arm NLST CXR arm**

**
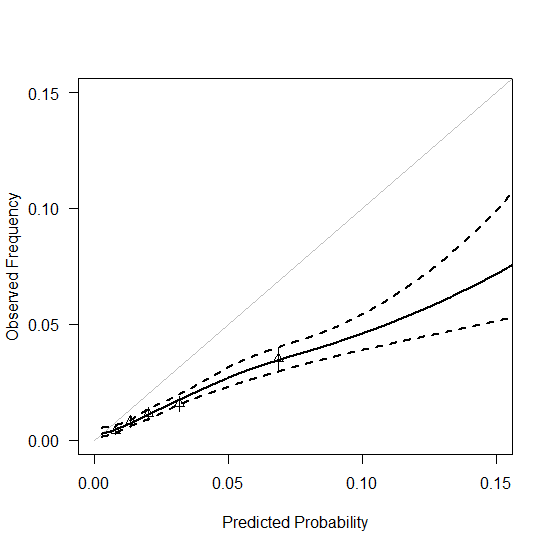
**
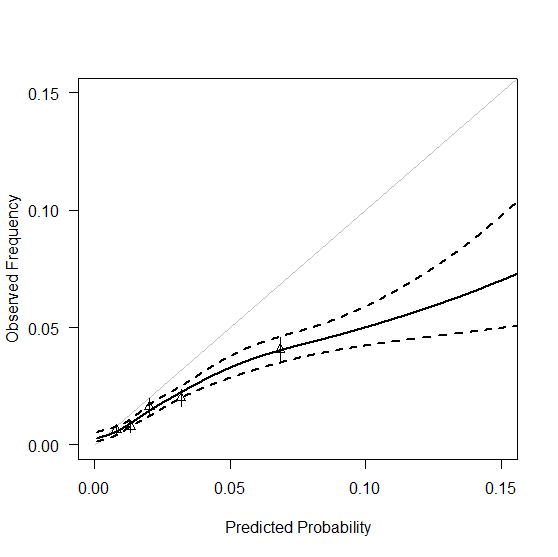


**PLCO CXR arm PLCO Control arm**


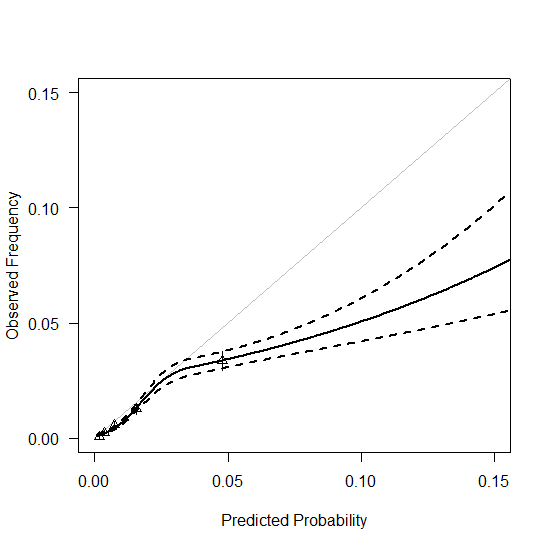

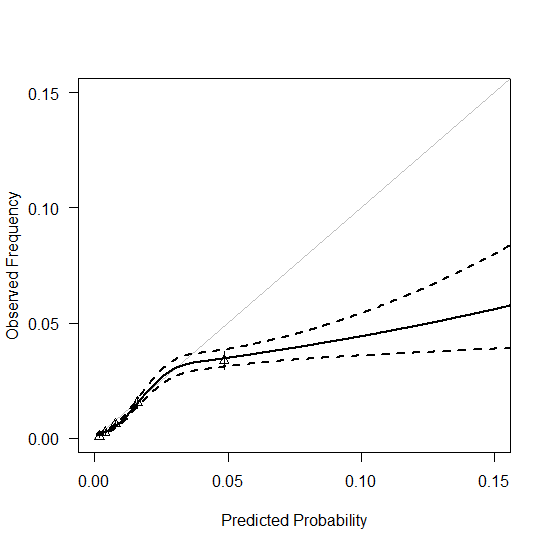


**Figure M: Calibration plots for the Knoke model for 6-year lung cancer incidence in all datasets**

**NLST CT arm NLST CXR arm**

**
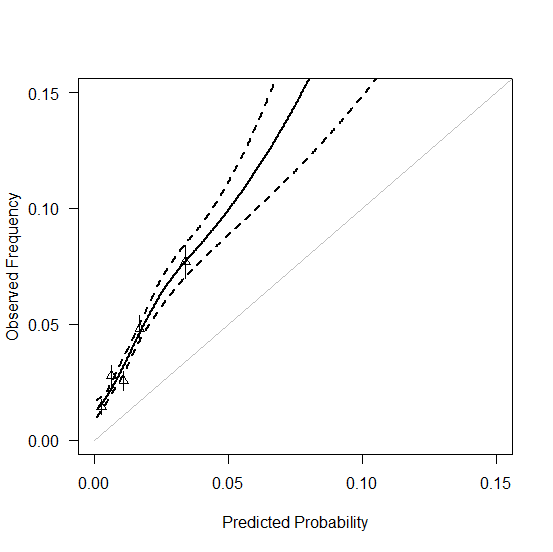
**
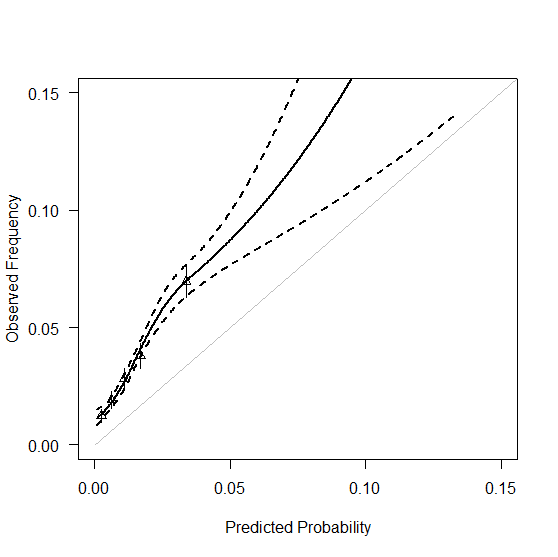


**PLCO CXR arm PLCO Control arm**


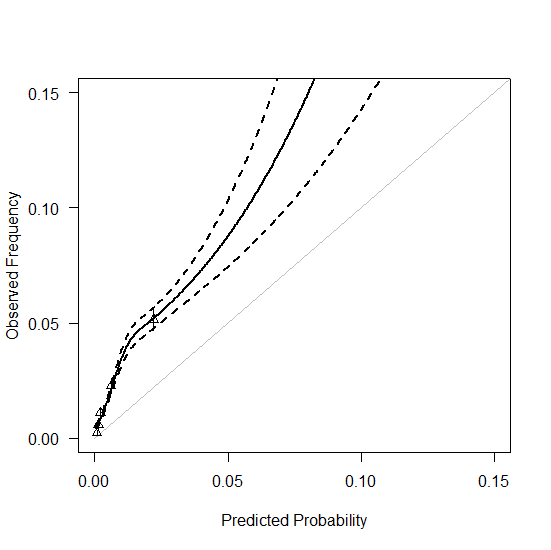

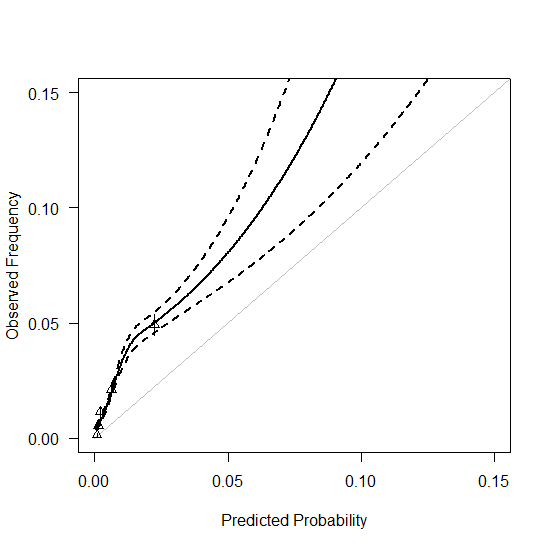


**Figure N: Calibration plots for the Knoke model for 6-year lung cancer mortality in all datasets**

**NLST CT arm NLST CXR arm**

**
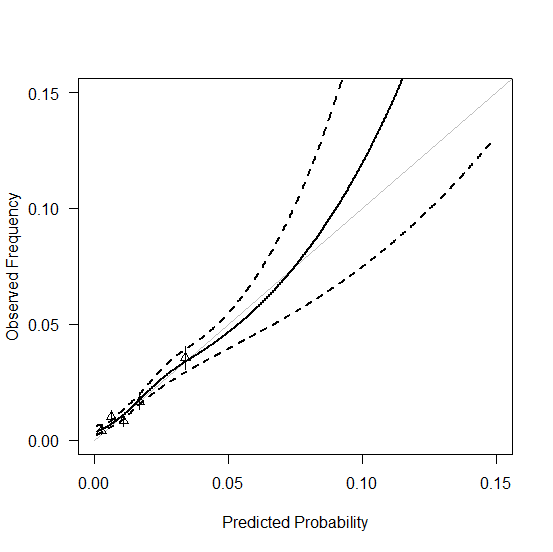
**
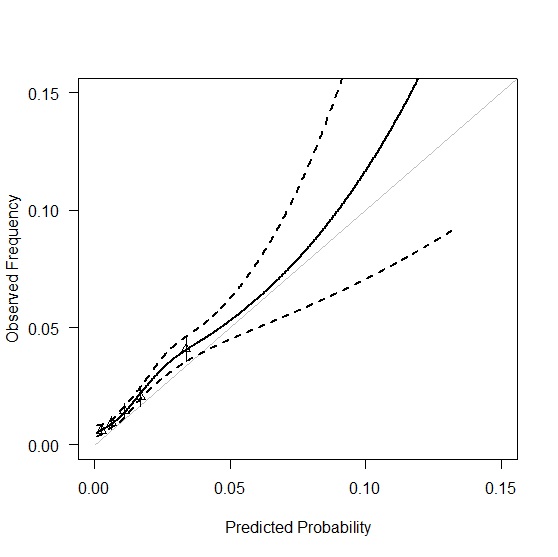


**PLCO CXR arm PLCO Control arm**


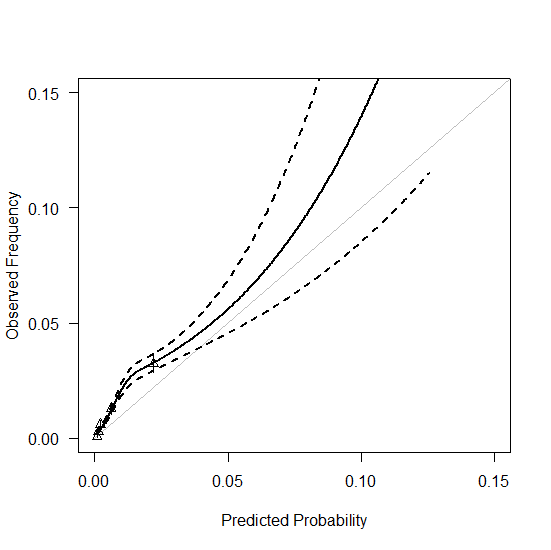

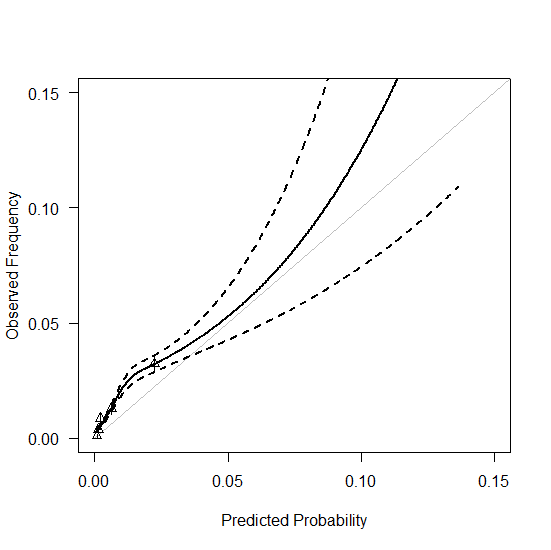


**Figure O: Calibration plots for the TSCE CPS lung cancer death model for 6-year lung cancer incidence in all datasets**

**NLST CT arm NLST CXR arm**

**
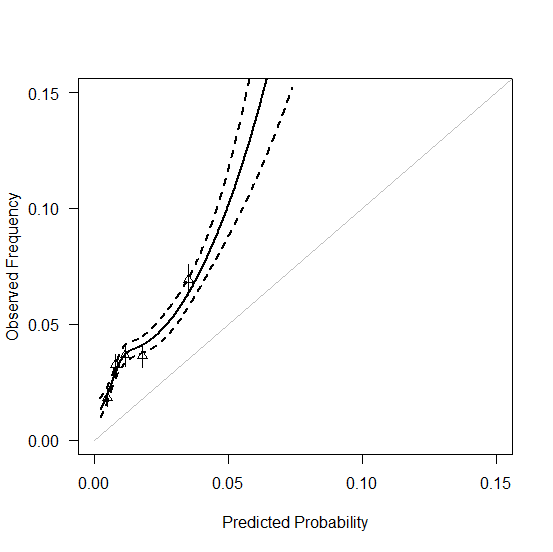
**
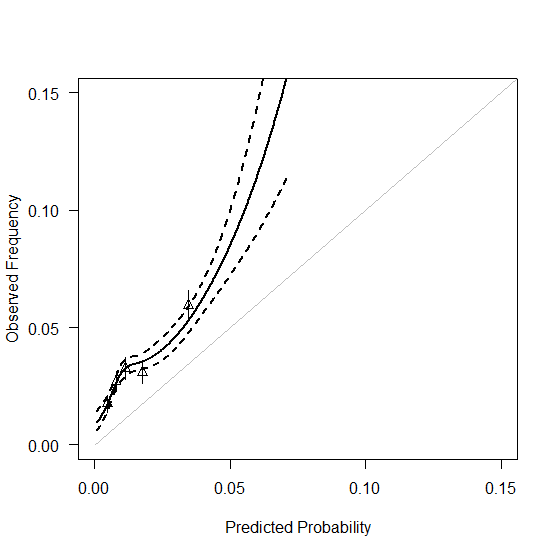


**PLCO CXR arm PLCO Control arm**

**
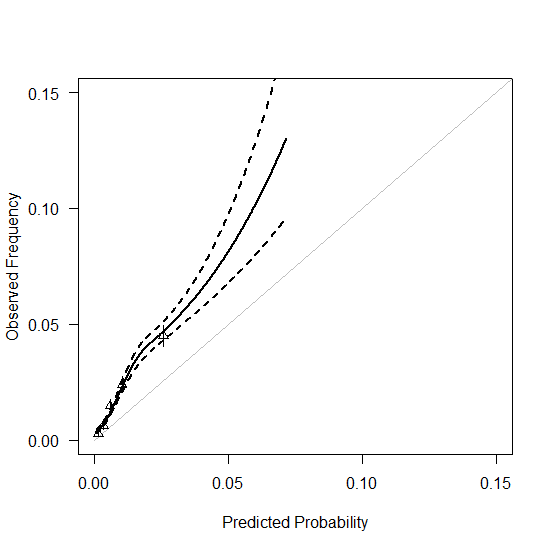

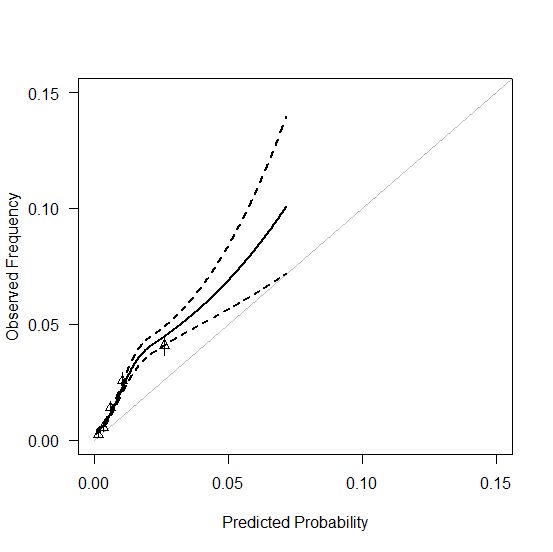
**

**Figure P: Calibration plots for the TSCE CPS lung cancer death model for 6-year lung cancer mortality in all datasets**

**NLST CT arm NLST CXR arm**

**
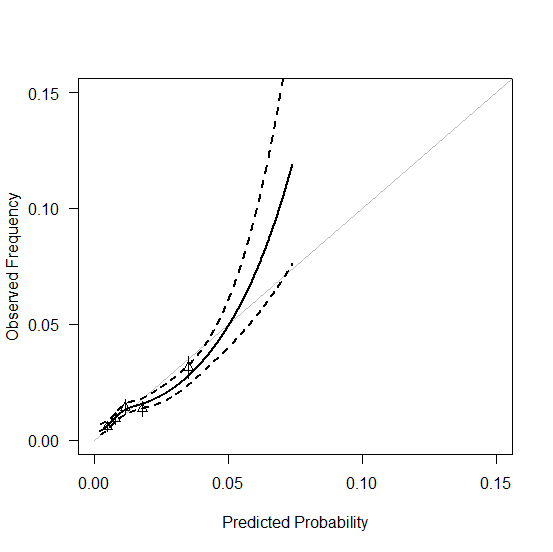
**
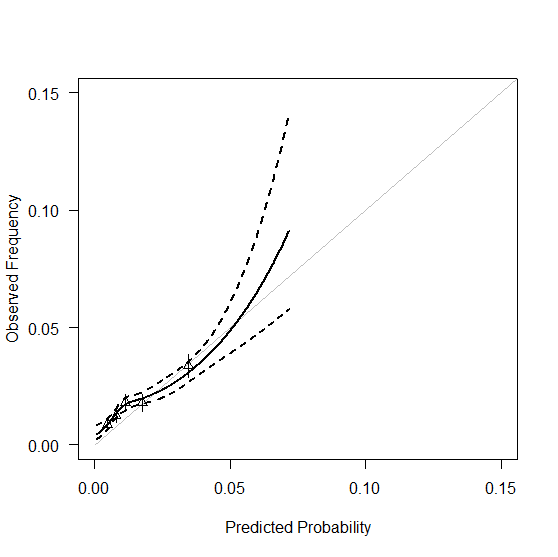


**PLCO CXR arm PLCO Control arm**

**
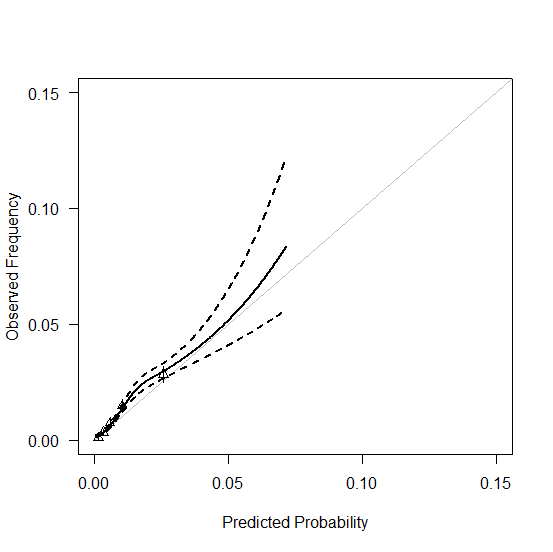

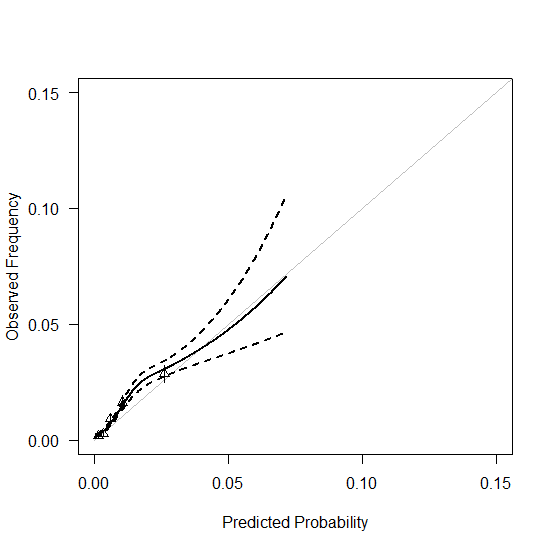
**

**Figure Q: Calibration plots for the TSCE NHS/HPFS lung cancer death model for 6-year lung cancer incidence in all datasets**

**NLST CT arm NLST CXR arm**

**
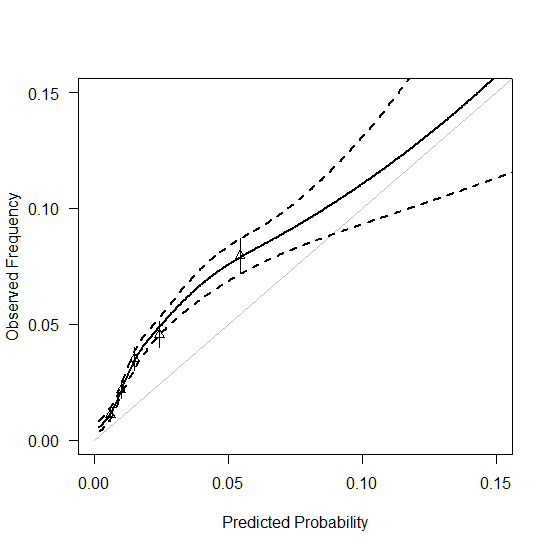
**
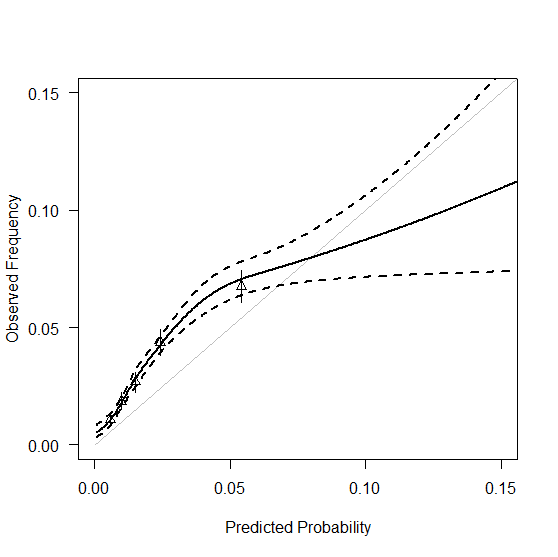


**PLCO CXR arm PLCO Control arm**

**
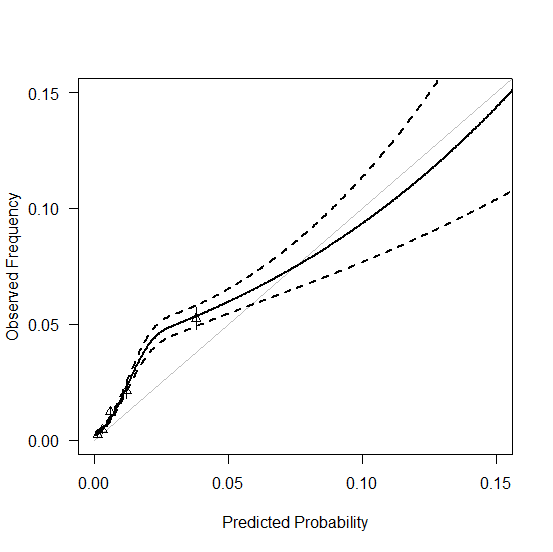

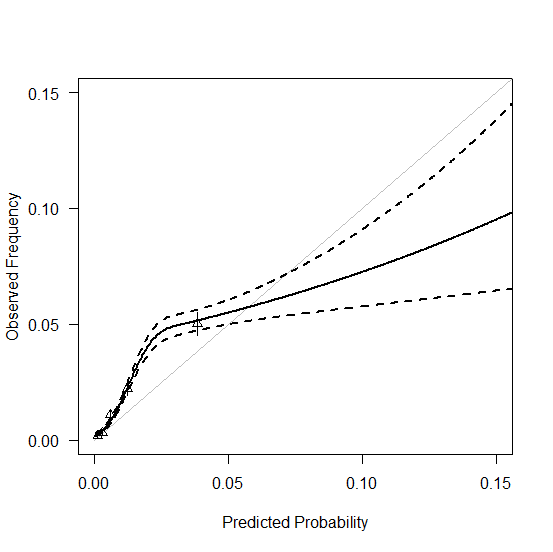
**

**Figure R: Calibration plots for the TSCE NHS/HPFS lung cancer death model for 6-year lung cancer mortality in all datasets**

**NLST CT arm NLST CXR arm**

**
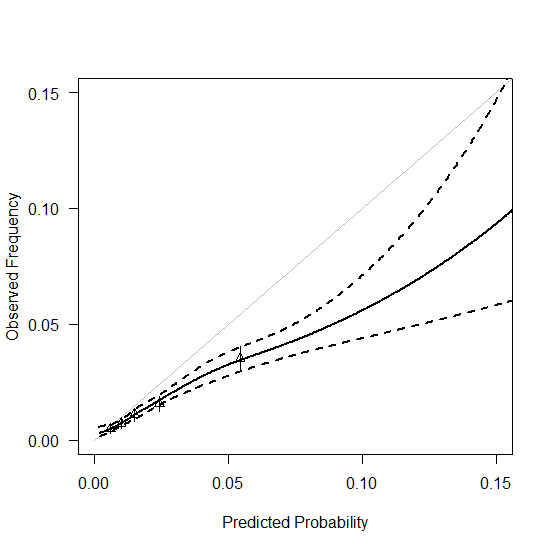
**
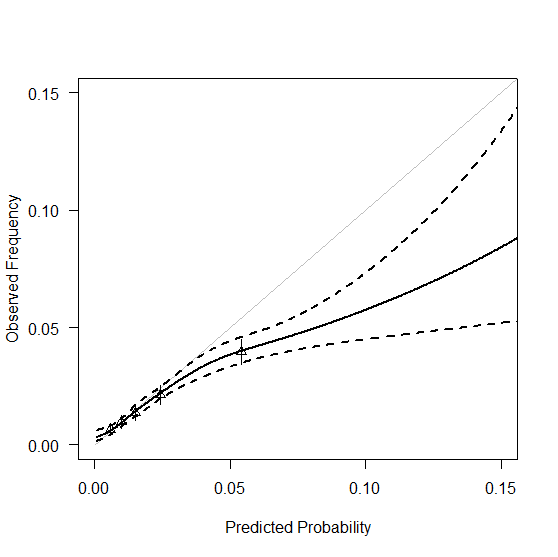


**PLCO CXR arm PLCO Control arm**

**
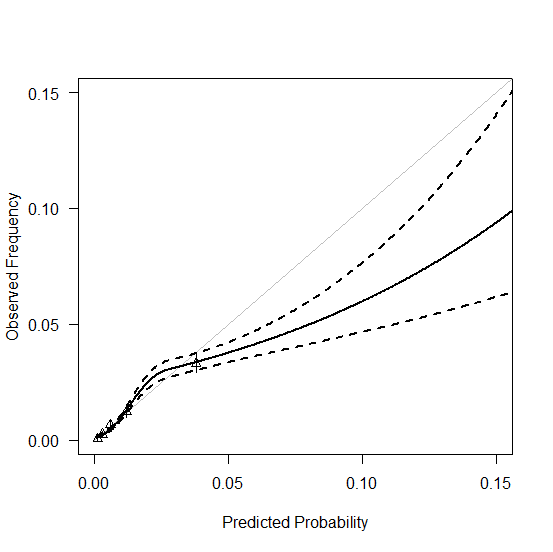

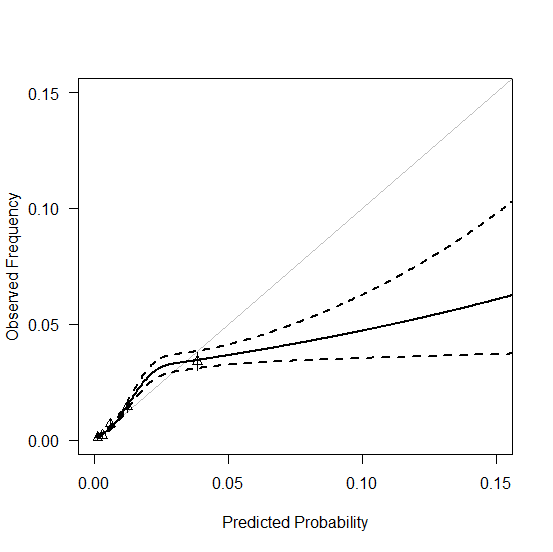
**

**Table A: Calibration intercepts of models for 6-year lung cancer incidence**

| **Intercepts** | **NLST (CT-arm)** | **NLST (CXR arm)** | **PLCO (CXR arm)** | **PLCO (Control arm)** |
| --- | --- | --- | --- | --- |
| **Bach model** | 0.16 | 0.01 | 0.06 | -0.01 |
| **LLP model** | 0.22 | 0.07 | -0.12 | -0.19 |
| **Simplified LLP model** | 0.47 | 0.32 | 0.1 | 0.03 |
| **PLCOm2102 model** | 0.13 | -0.02 | 0.06 | 0.00 |
| **Simplified PLCOm2102 model** | 0.45 | 0.30 | 0.32 | 0.26 |
| **TSCE incidence model** | 0.33 | 0.18 | 0.20 | 0.13 |
| **Knoke model** | 1.06 | 0.91 | 0.78 | 1.02 |
| **TSCE CPS death model** | 0.96 | 0.82 | 0.72 | 0.66 |
| **TSCE NHS/HPFS death model** | 0.60 | 0.45 | 0.47 | 0.40 |

**Table B: Calibration slopes of models for 6-year lung cancer incidence**

| **Slopes** | **NLST (CT-arm)** | **NLST (CXR arm)** | **PLCO (CXR arm)** | **PLCO (Control arm)** |
| --- | --- | --- | --- | --- |
| **Bach model** | 1.04 | 1.09 | 0.99 | 0.99 |
| **LLP model** | 0.68 | 0.73 | 1.05 | 1.05 |
| **Simplified LLP model** | 0.76 | 0.80 | 1.07 | 1.05 |
| **PLCOm2102 model** | 0.87 | 0.91 | 0.94 | 0.98 |
| **Simplified PLCOm2102 model** | 1.03 | 1.04 | 1.02 | 1.04 |
| **TSCE incidence model** | 0.80 | 0.79 | 0.87 | 0.87 |
| **Knoke model** | 0.70 | 0.72 | 1.09 | 0.79 |
| **TSCE CPS death model** | 0.63 | 0.59 | 0.90 | 0.89 |
| **TSCE NHS/HPFS death model** | 0.77 | 0.76 | 0.85 | 0.84 |

**Table C: Calibration intercepts of models for 6-year lung cancer mortality**

| **Intercepts** | **NLST (CT-arm)** | **NLST (CXR arm)** | **PLCO (CXR arm)** | **PLCO (Control arm)** |
| --- | --- | --- | --- | --- |
| **Bach model** | -0.83 | -0.62 | -0.49 | -0.44 |
| **LLP model** | -0.77 | -0.57 | -0.67 | -0.61 |
| **Simplified LLP model** | -0.52 | -0.31 | -0.44 | -0.39 |
| **PLCOm2102 model** | -0.87 | -0.66 | -0.49 | -0.42 |
| **Simplified PLCOm2102 model** | -0.54 | -0.33 | -0.22 | -0.17 |
| **TSCE incidence model** | -0.67 | -0.46 | -0.35 | -0.30 |
| **Knoke model** | 0.06 | 0.27 | 0.53 | 0.59 |
| **TSCE CPS death model** | -0.03 | 0.19 | 0.18 | 0.23 |
| **TSCE NHS/HPFS death model** | -0.40 | -0.19 | -0.08 | -0.03 |

**Table D: Calibration slopes of models for 6-year lung cancer mortality**

| **Slopes** | **NLST (CT-arm)** | **NLST (CXR arm)** | **PLCO (CXR arm)** | **PLCO (Control arm)** |
| --- | --- | --- | --- | --- |
| **Bach model** | 1.21 | 1.17 | 1.12 | 0.97 |
| **LLP model** | 0.85 | 0.69 | 1.18 | 1.09 |
| **Simplified LLP model** | 0.91 | 0.79 | 1.21 | 1.09 |
| **PLCOm2102 model** | 1.01 | 0.92 | 1.01 | 0.95 |
| **Simplified PLCOm2102 model** | 1.19 | 1.10 | 1.11 | 1.02 |
| **TSCE incidence model** | 0.91 | 0.84 | 0.94 | 0.86 |
| **Knoke model** | 0.89 | 0.80 | 0.85 | 0.76 |
| **TSCE CPS death model** | 0.82 | 0.65 | 0.99 | 0.92 |
| **TSCE NHS/HPFS death model** | 0.87 | 0.80 | 0.92 | 0.84 |
