# Supplementary material for: Risk prediction models for selection of lung cancer screening candidates: A retrospective validation study
Source: PLoS Med. 2017 Apr 4;14(4):e1002277. doi: 10.1371/journal.pmed.1002277 (PMC5380315; doi:10.1371/journal.pmed.1002277)
Supplement: S5 Appendix — (DOCX) [file pmed.1002277.s005.docx]

**S5 Appendix: Discriminative performance of the investigated risk models, by dataset, predicted outcome and timeframe**

**Figure A: Area under the receiver operator curve (AUC) of the investigated risk models (with 95% confidence interval), by dataset predicted outcome and 5- and 6-year timeframe**


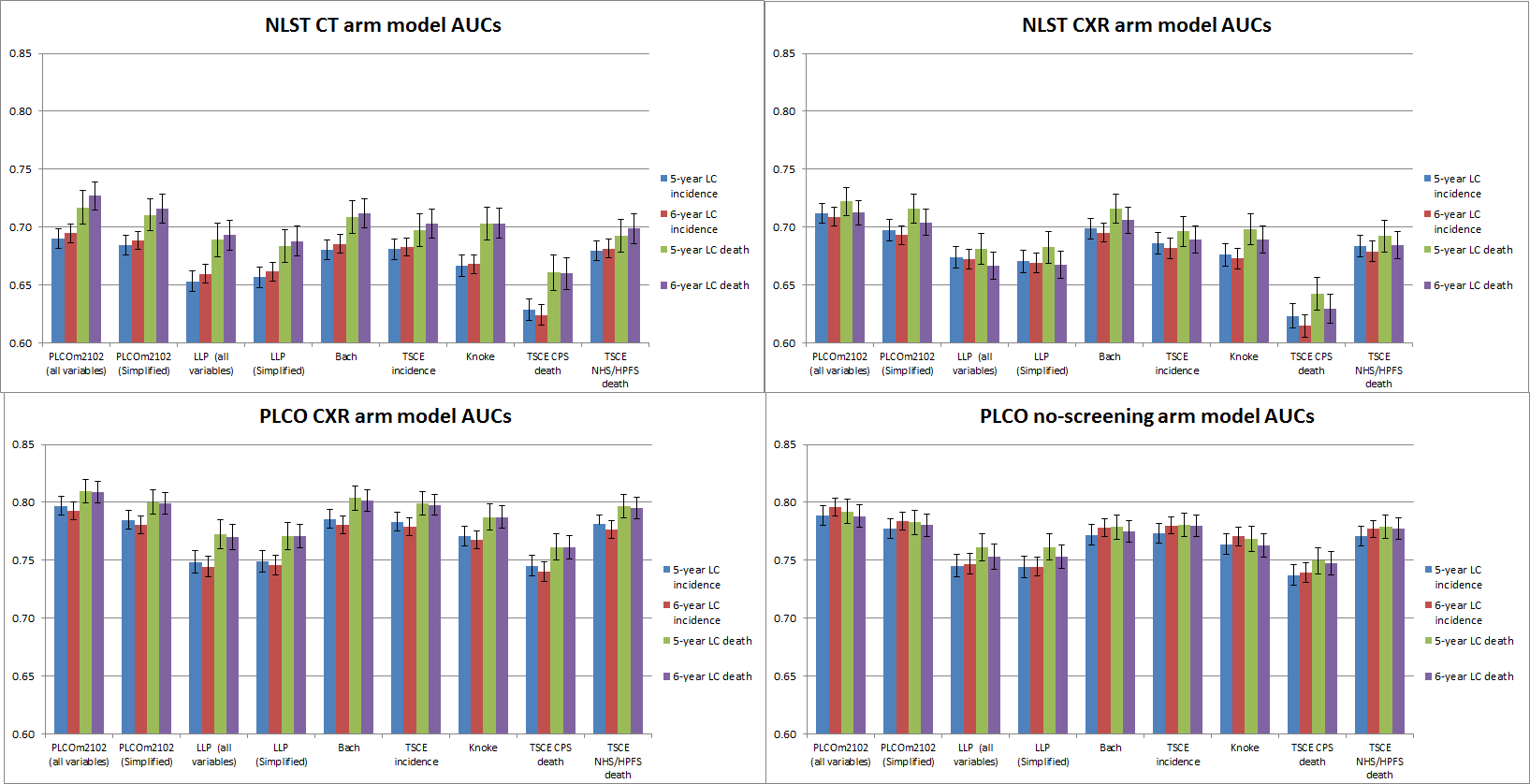


**Abbreviations:** National Lung Screening Trial (NLST); Prostate, Lung, Colorectal and Ovarian Cancer Screening Trial (PLCO); computed tomography (CT); chest radiography (CXR); Lung Cancer (LC).
