# Supplementary material for: Risk prediction models for selection of lung cancer screening candidates: A retrospective validation study
Source: PLoS Med. 2017 Apr 4;14(4):e1002277. doi: 10.1371/journal.pmed.1002277 (PMC5380315; doi:10.1371/journal.pmed.1002277)
Supplement: S6 Appendix — (DOCX) [file pmed.1002277.s006.docx]

**S6 Appendix: Decision curve analyses for the evaluated lung cancer risk prediction models for 6-year lung cancer incidence and mortality**

**Figure A: Decision curve analyses for the Bach model for 6-year lung cancer incidence in all datasets**

**NLST CT arm NLST CXR arm**

**
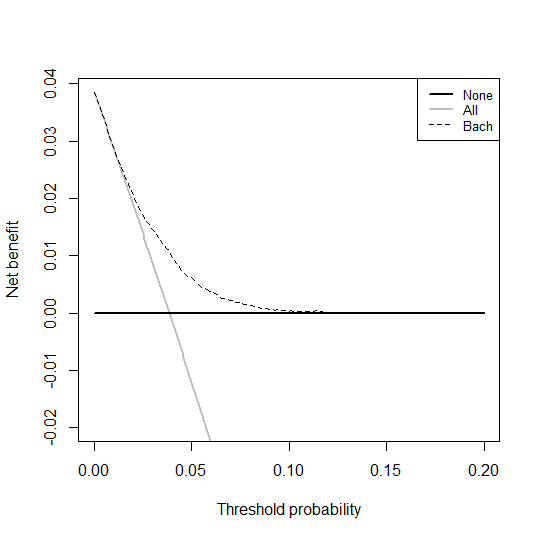
**
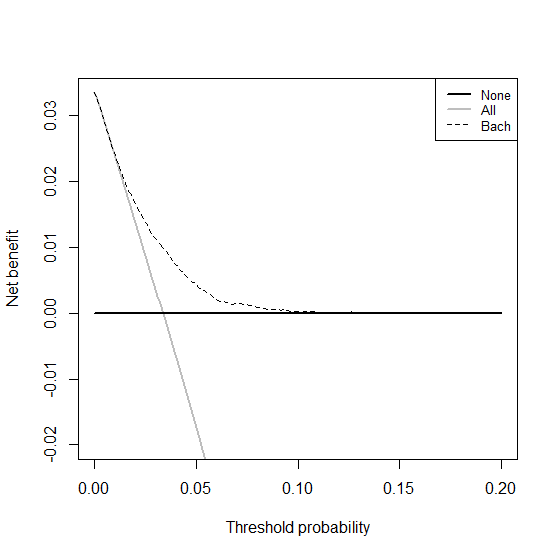


**PLCO CXR arm PLCO Control arm**

**
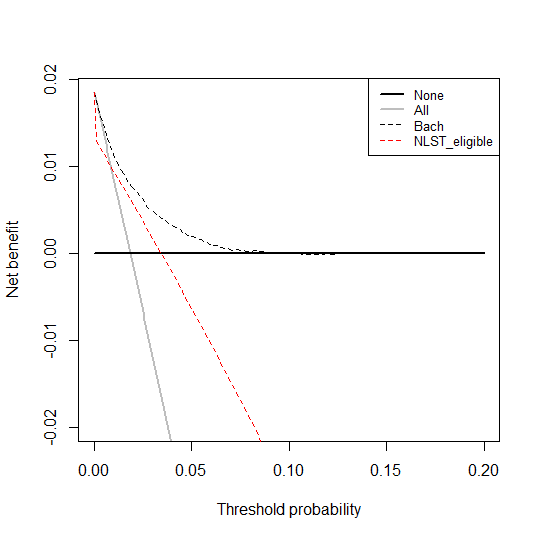

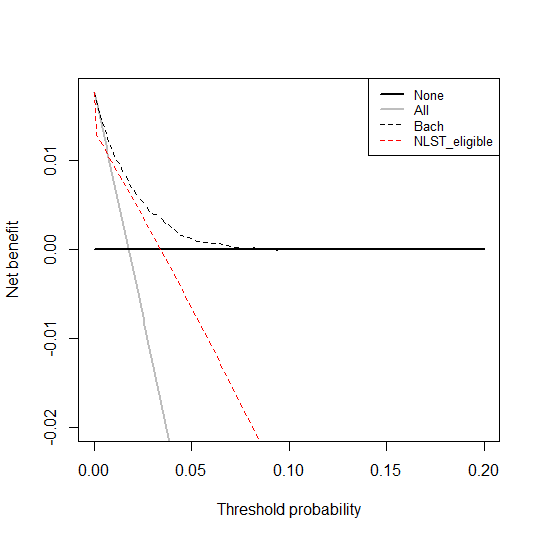
**

**Figure B: Decision curve analyses for the Bach model for 6-year lung cancer mortality in all datasets**

**NLST CT arm NLST CXR arm
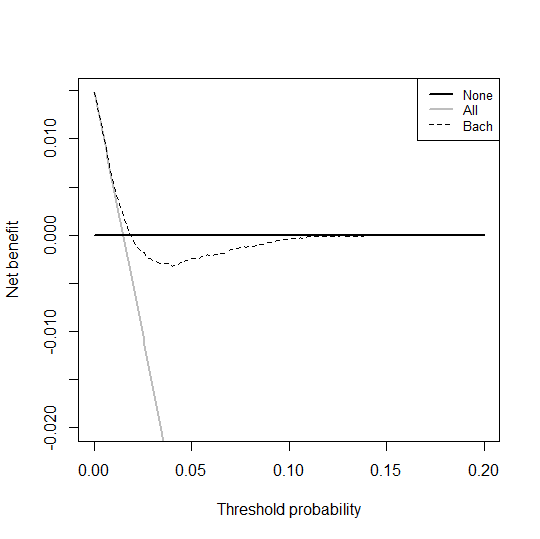
**
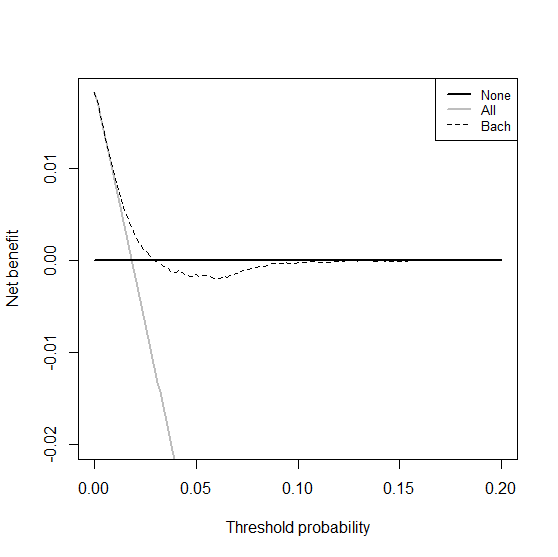
 **PLCO CXR arm PLCO Control arm
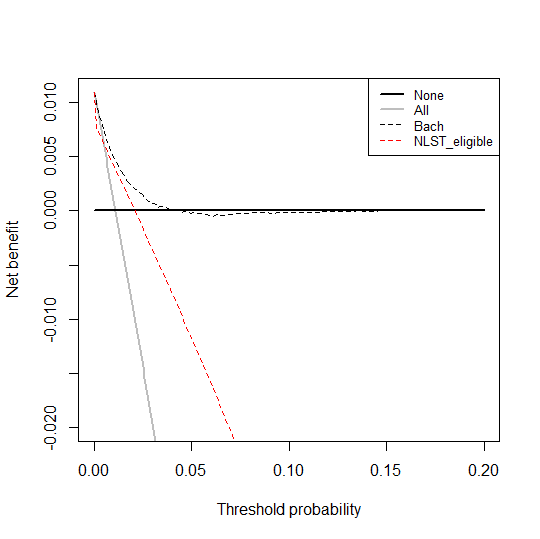

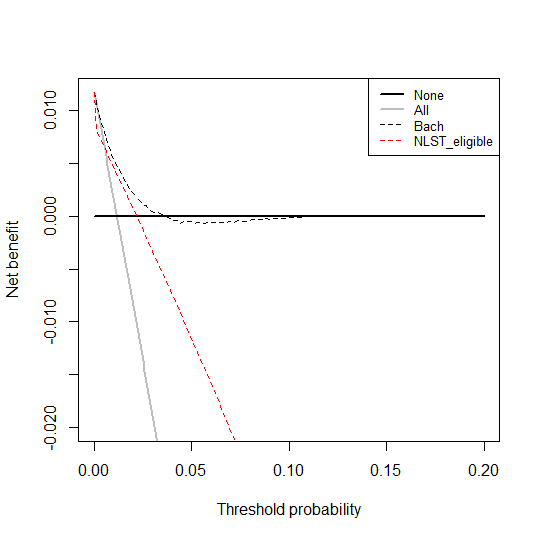
**

**Figure C: Decision curve analyses for the LLP model for 6-year lung cancer incidence in all datasets**

**NLST CT arm NLST CXR arm
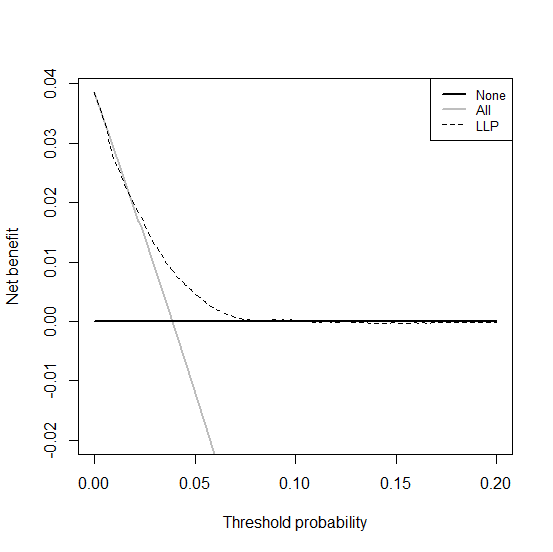
**
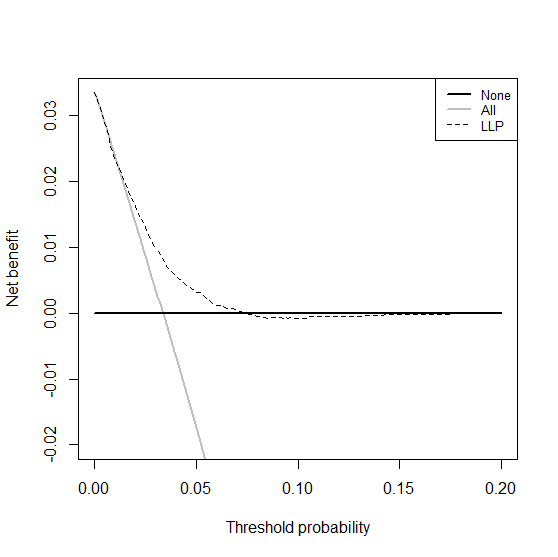
 **PLCO CXR arm PLCO Control arm
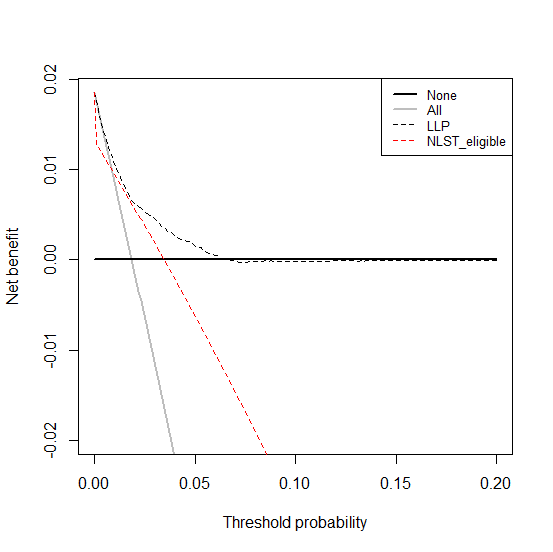

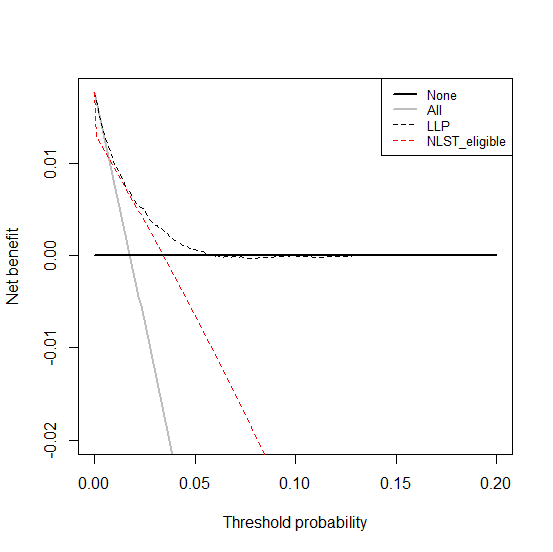
**

**Figure D: Decision curve analyses for the LLP model for 6-year lung cancer mortality in all datasets**

**NLST CT arm NLST CXR arm**

**
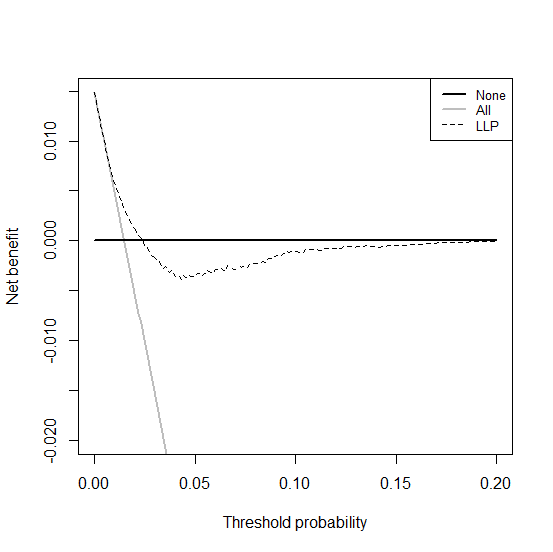
**
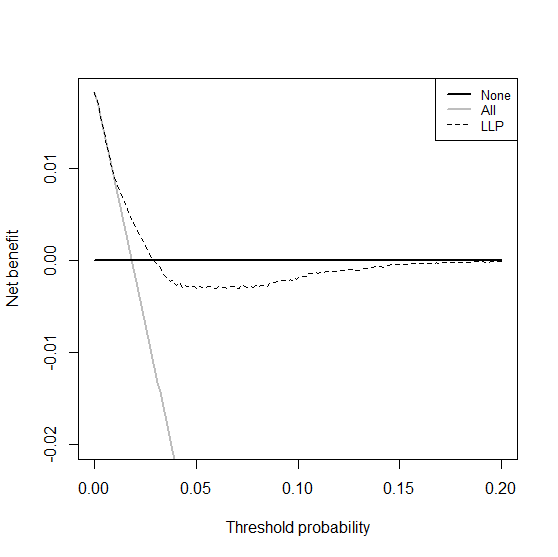


**PLCO CXR arm PLCO Control arm**

**
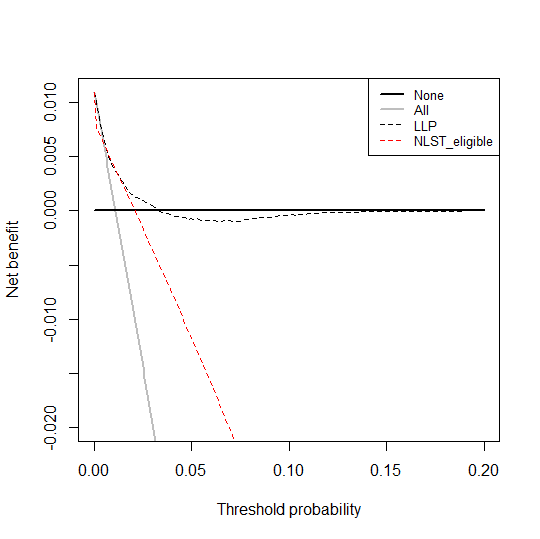

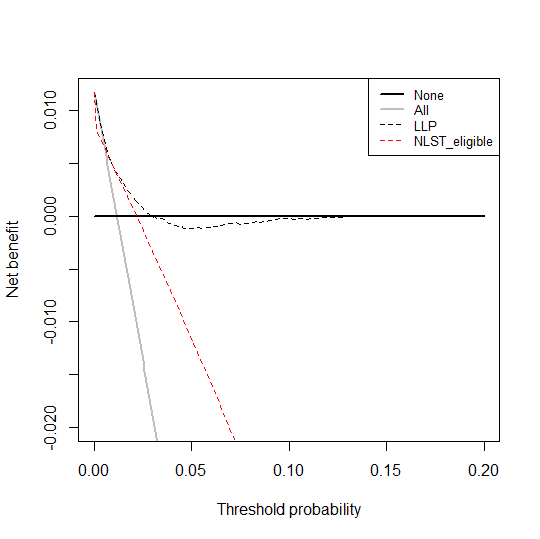
**

**Figure E: Decision curve analyses for the simplified LLP model for 6-year lung cancer incidence in all datasets**

**NLST CT arm NLST CXR arm**

**
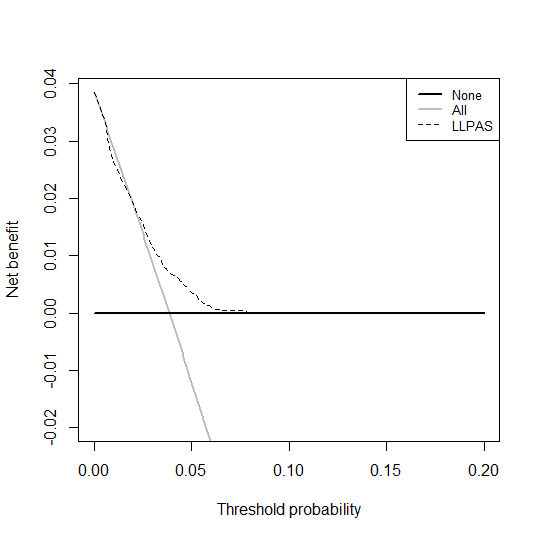
**
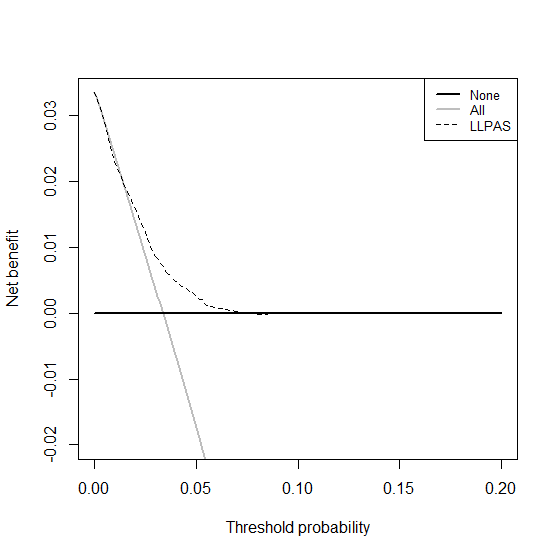


**PLCO CXR arm PLCO Control arm**

**
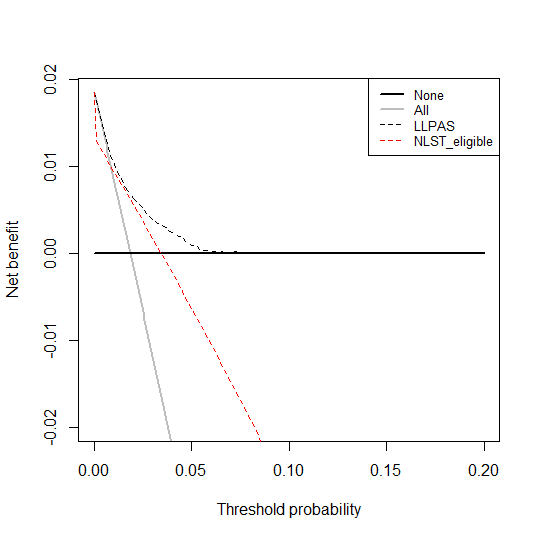

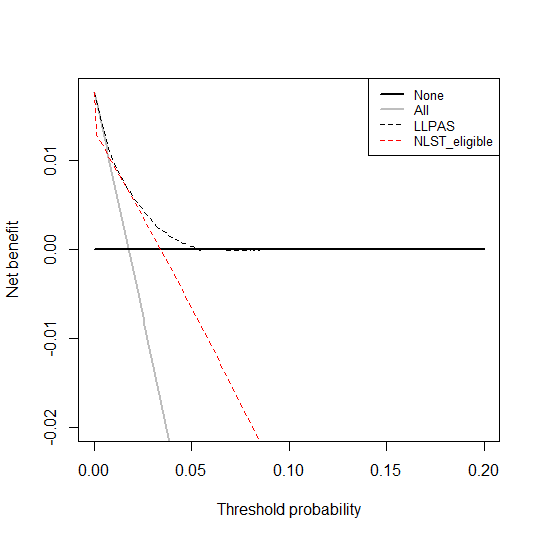
**

**Figure F: Decision curve analyses for the simplified LLP model for 6-year lung cancer mortality in all datasets**

**NLST CT arm NLST CXR arm**

**
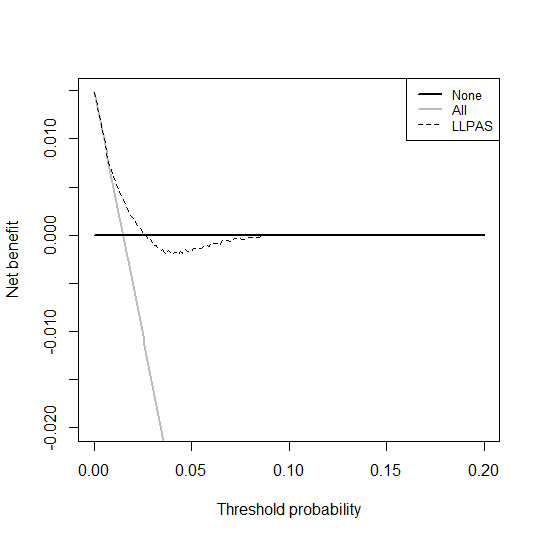
**
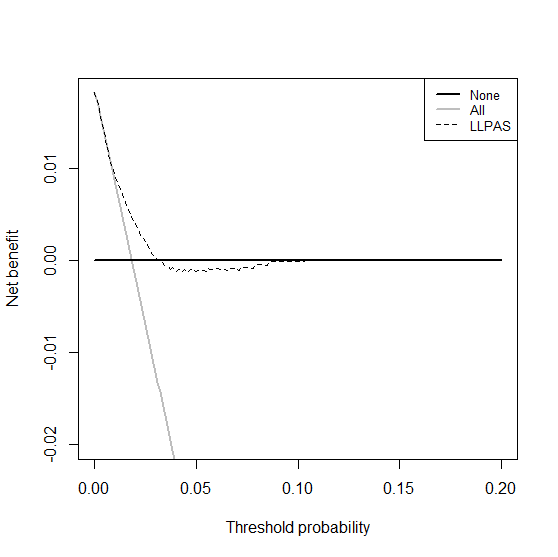


**PLCO CXR arm PLCO Control arm**

**
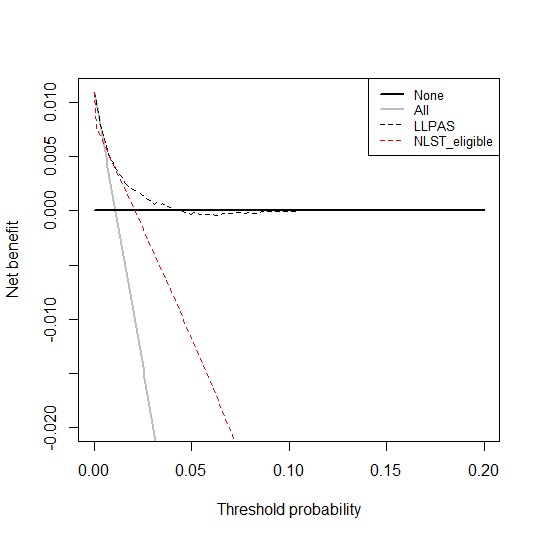

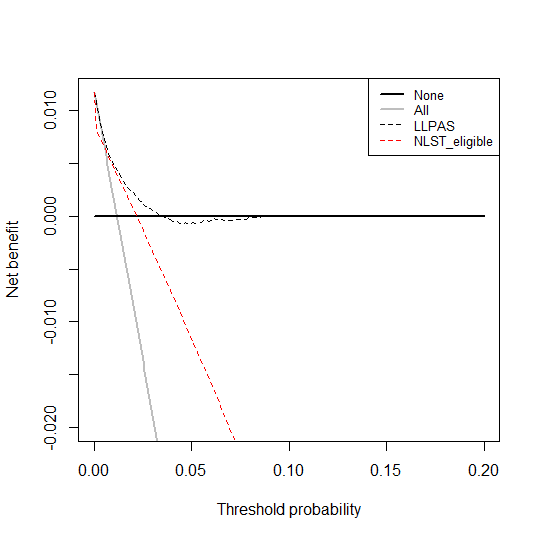
**

**Figure G: Decision curve analyses for the PLCOm2012 model for 6-year lung cancer incidence in all datasets**

**NLST CT arm NLST CXR arm**

**
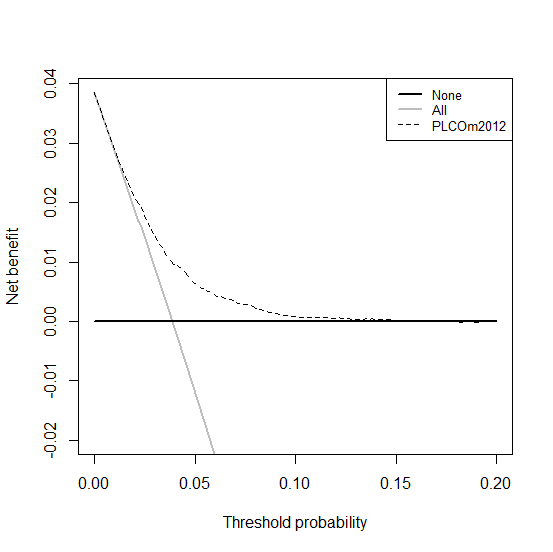
**
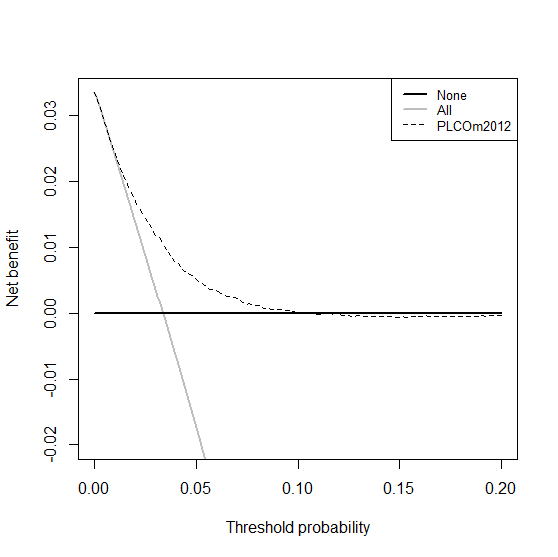


**PLCO CXR arm PLCO Control arm**

**
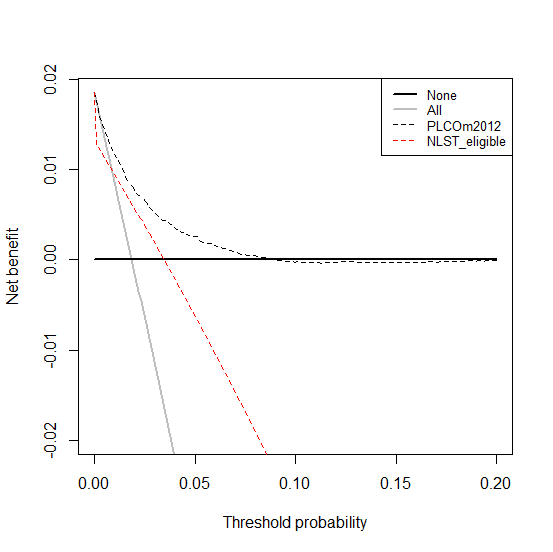
** **
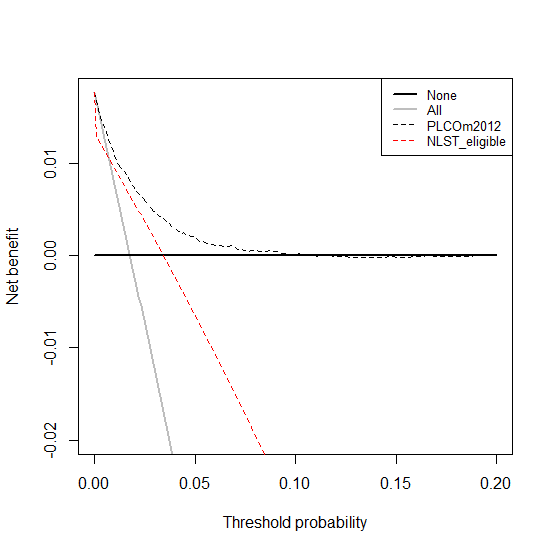
**

**Figure H: Decision curve analyses for the PLCOm2012 model for 6-year lung cancer mortality in all datasets**

**NLST CT arm NLST CXR arm**

**
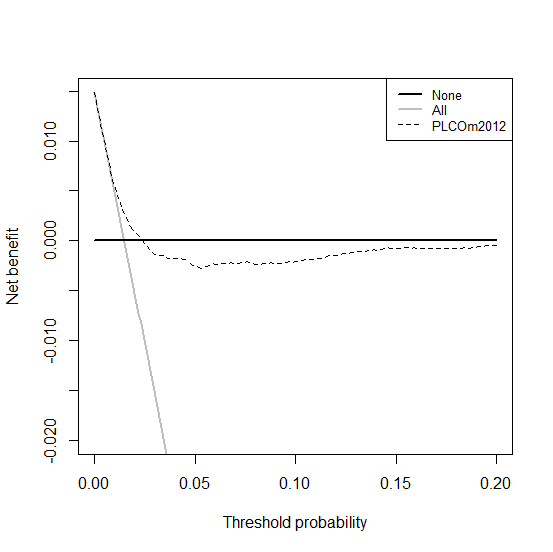
**
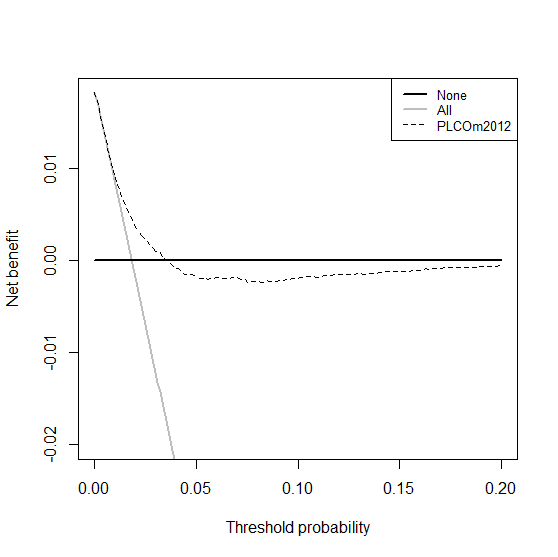


**PLCO CXR arm PLCO Control arm**

**
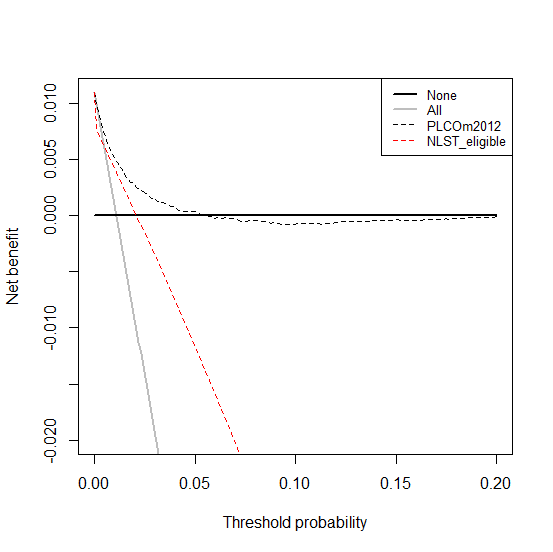
** **
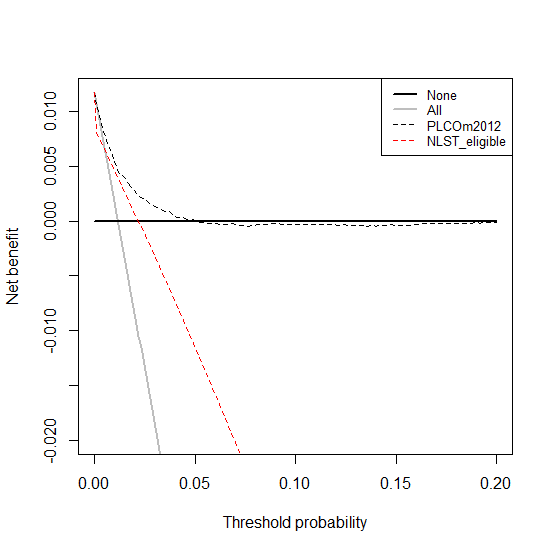
**

**Figure I: Decision curve analyses for the simplified PLCOm2012 model for 6-year lung cancer incidence in all datasets**

**NLST CT arm NLST CXR arm**

**
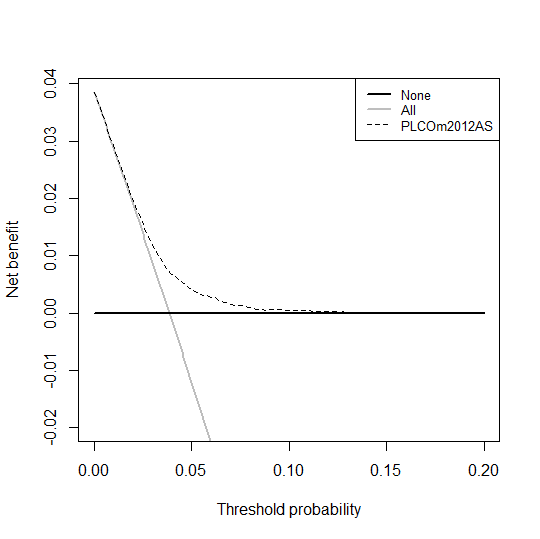
**
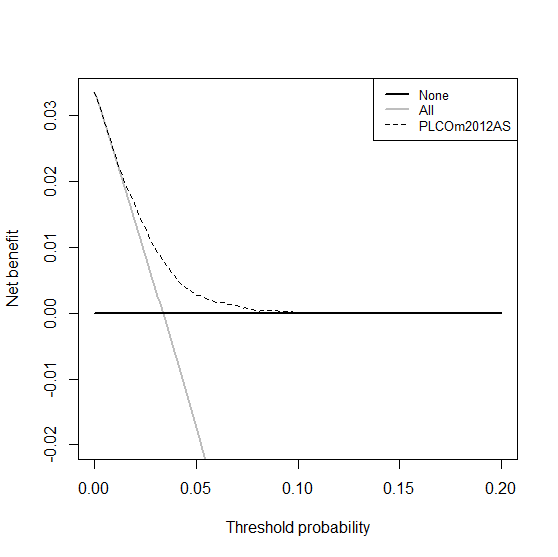


**PLCO CXR arm PLCO Control arm
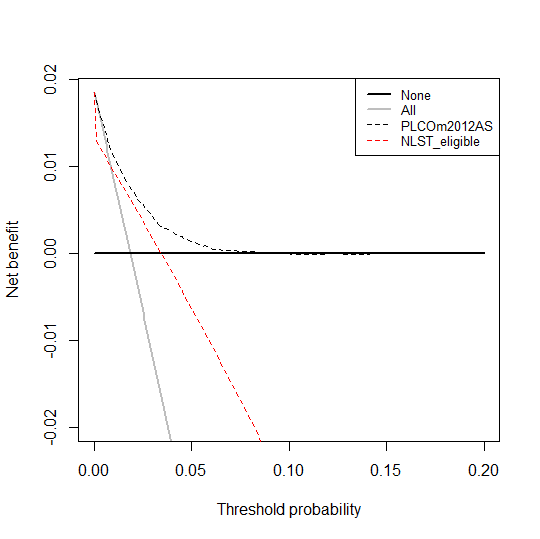

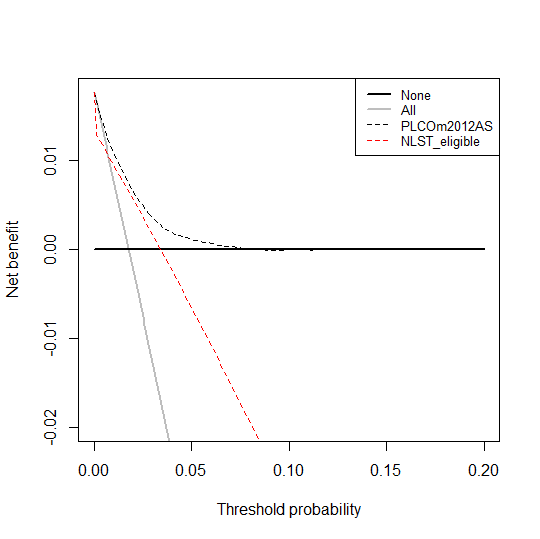
**

**Figure J: Decision curve analyses for the simplified PLCOm2012 model for 6-year lung cancer mortality in all datasets**

**NLST CT arm NLST CXR arm**

**
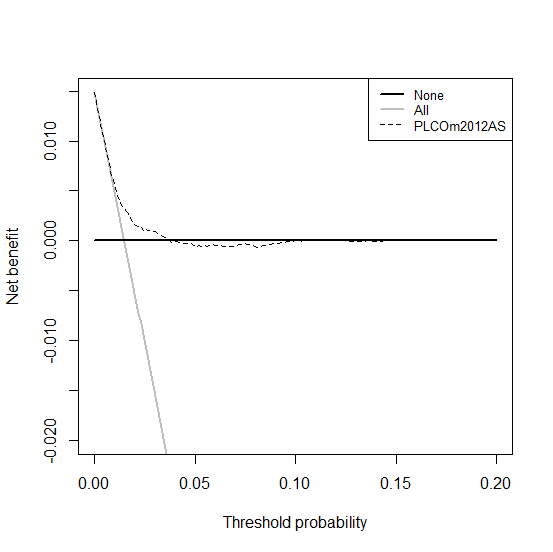
**
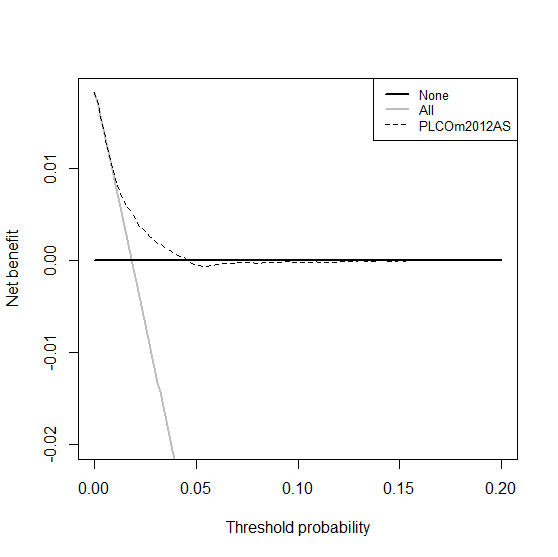


**PLCO CXR arm PLCO Control arm
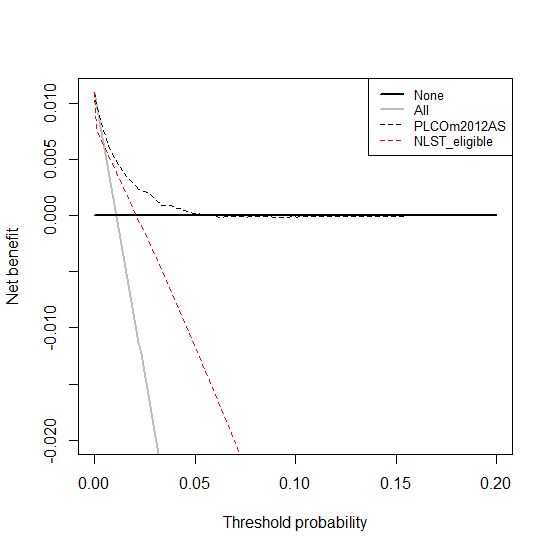

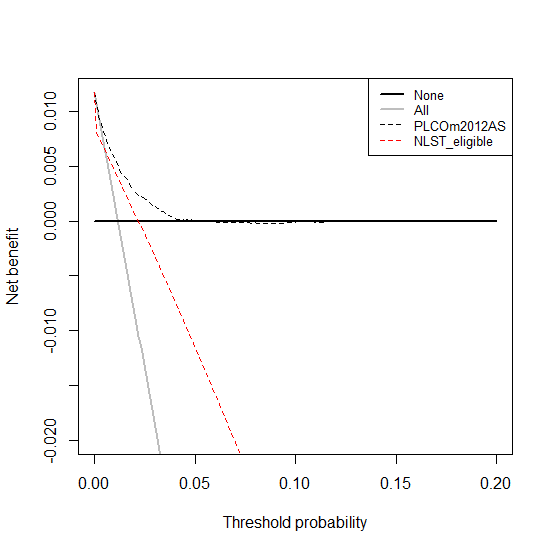
**

**Figure K: Decision curve analyses for the TSCE incidence model for 6-year lung cancer incidence in all datasets**

**NLST CT arm NLST CXR arm**

**
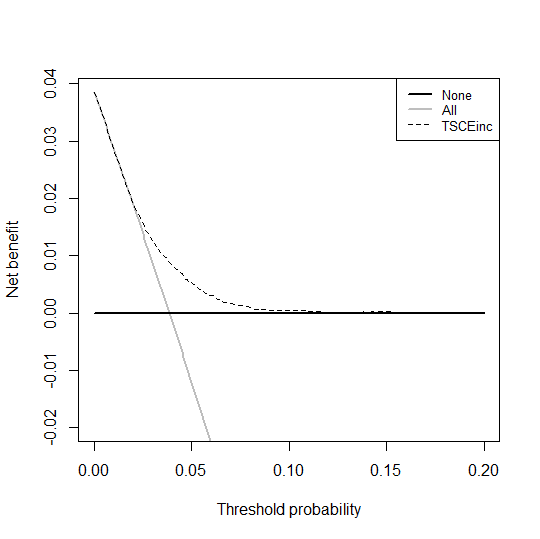
**
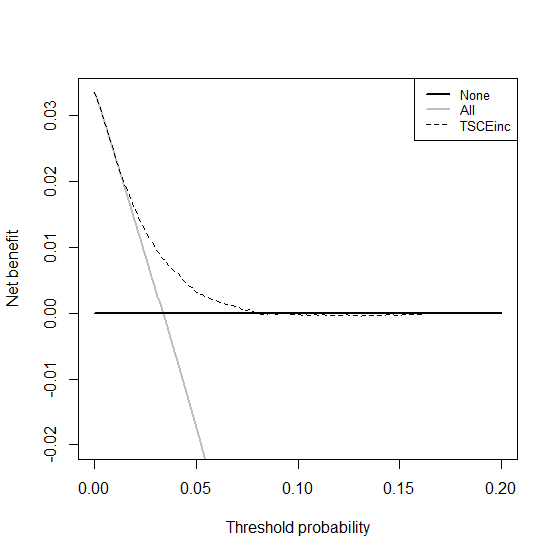


**PLCO CXR arm PLCO Control arm**


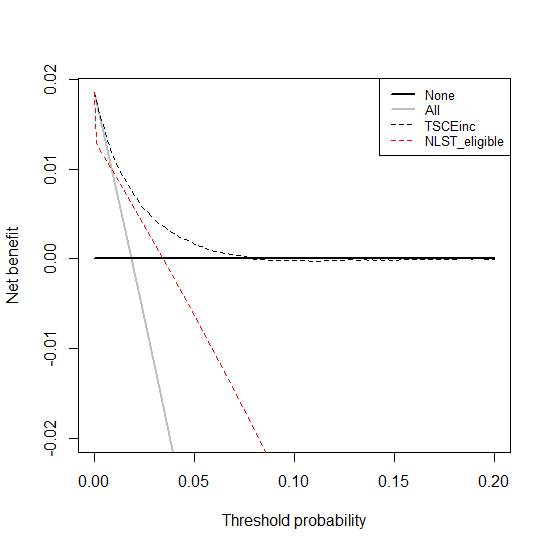

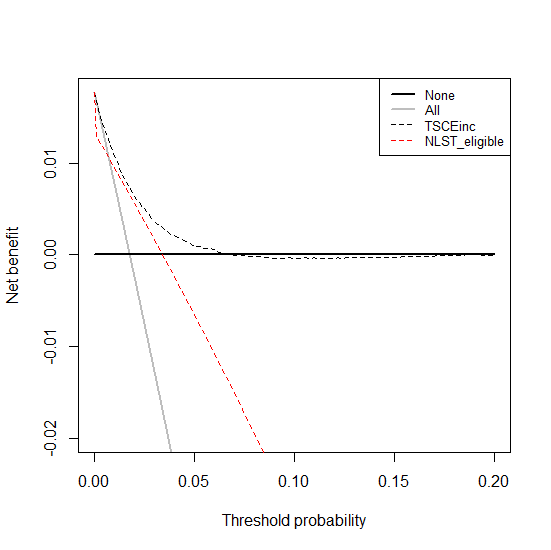


**Figure L: Decision curve analyses for the TSCE incidence model for 6-year lung cancer mortality in all datasets**

**NLST CT arm NLST CXR arm**

**
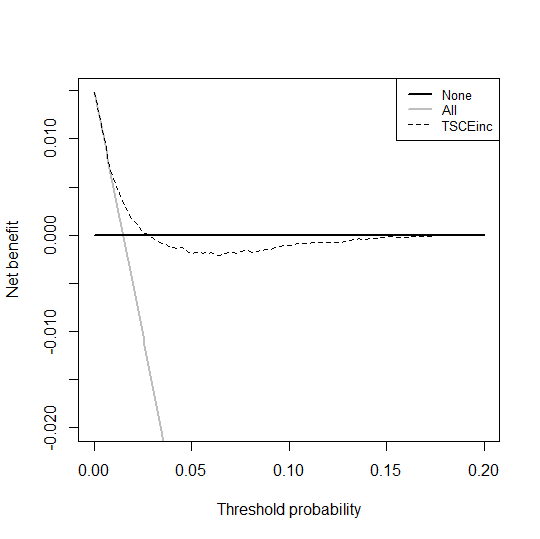
**
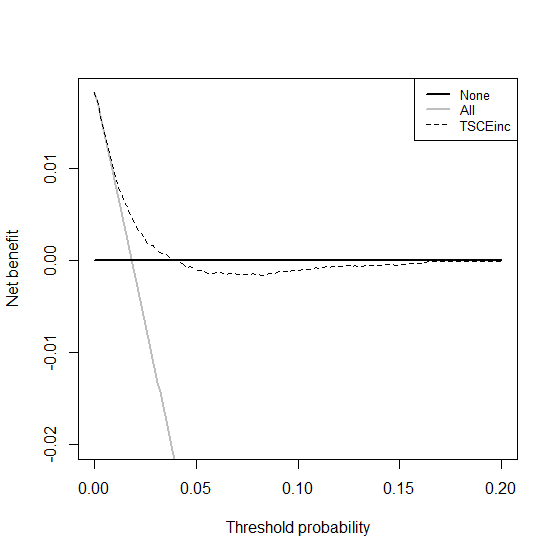


**PLCO CXR arm PLCO Control arm**


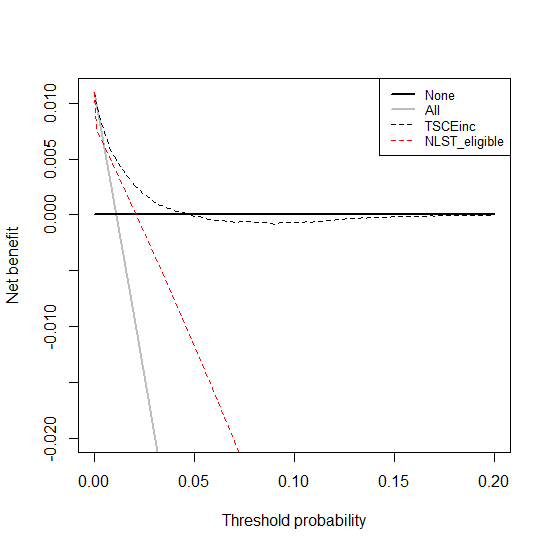

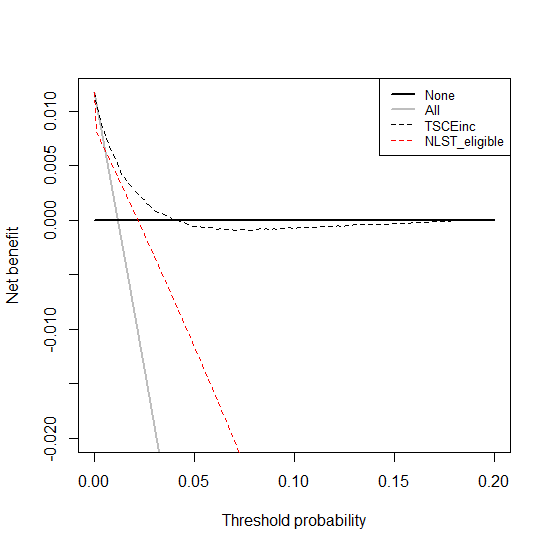


**Figure M: Decision curve analyses for the Knoke model for 6-year lung cancer incidence in all datasets**

**NLST CT arm NLST CXR arm**

**
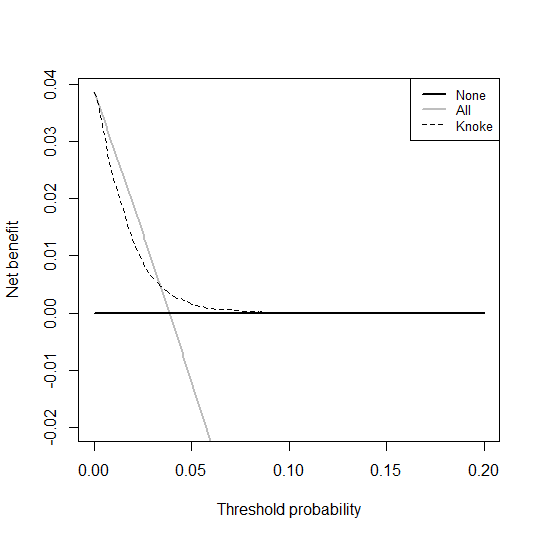
**
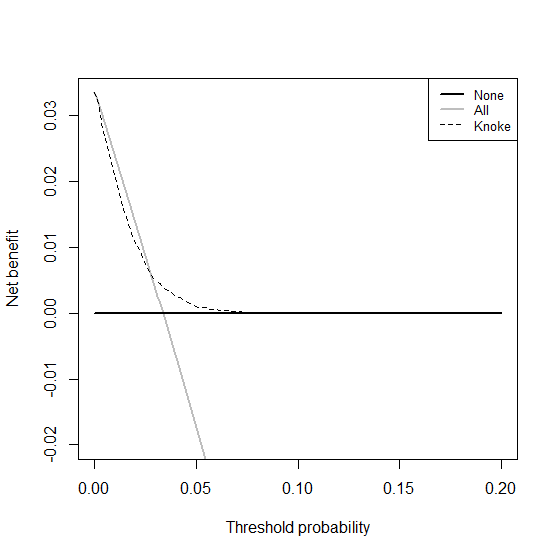


**PLCO CXR arm PLCO Control arm**


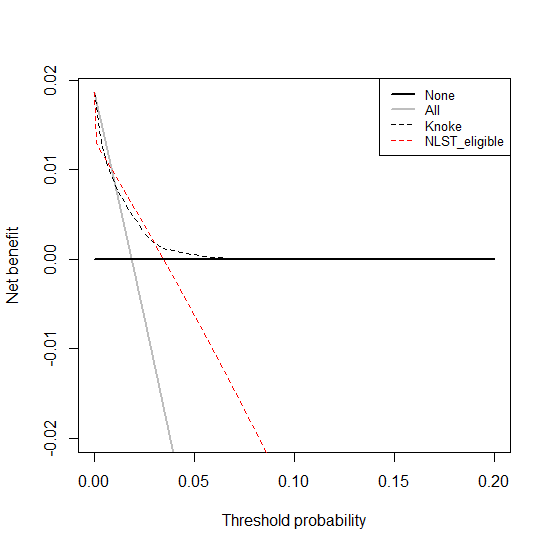

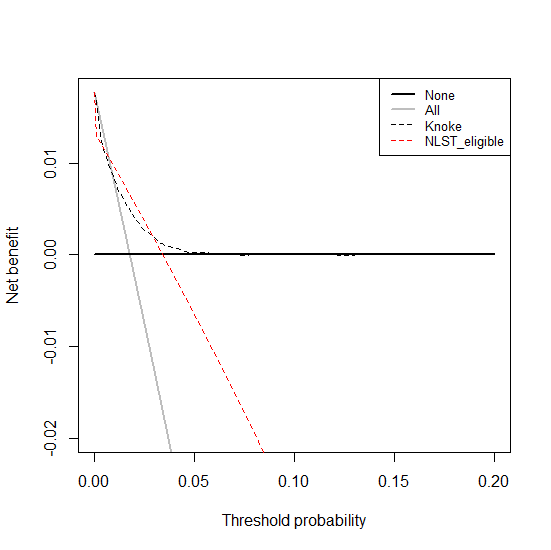


**Figure N: Decision curve analyses for the Knoke model for 6-year lung cancer mortality in all datasets**

**NLST CT arm NLST CXR arm**

**
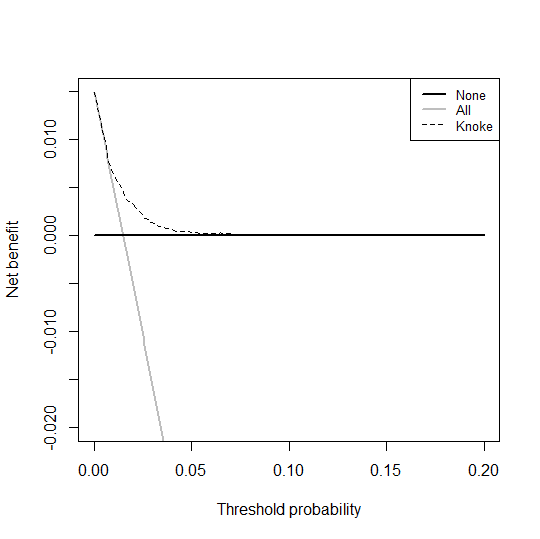
**
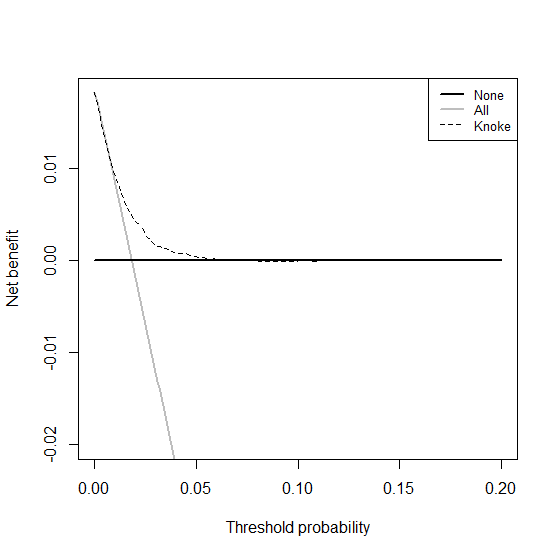


**PLCO CXR arm PLCO Control arm**


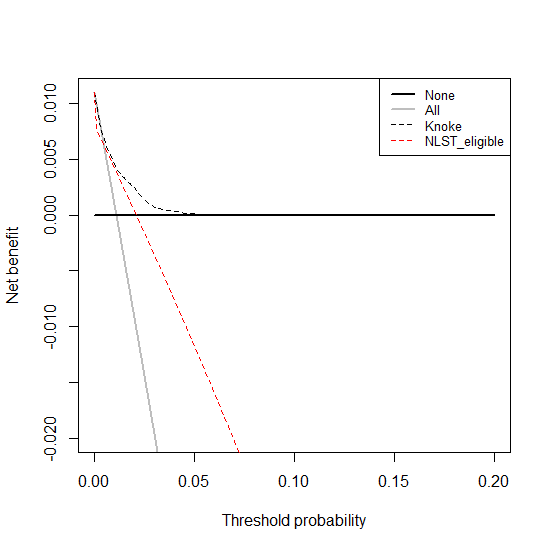

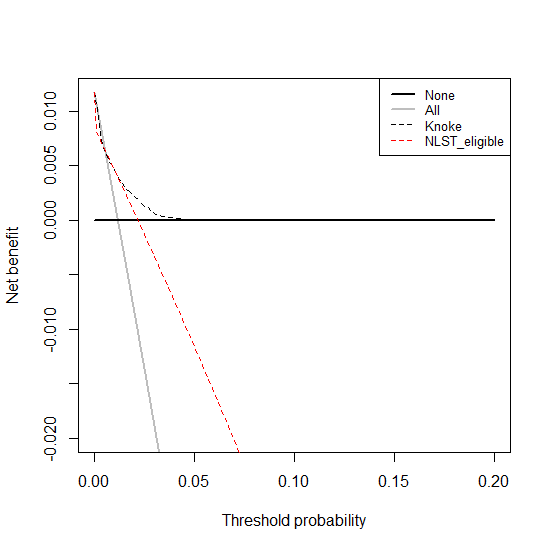


**Figure O: Decision curve analyses for the TSCE CPS lung cancer death model for 6-year lung cancer incidence in all datasets**

**NLST CT arm NLST CXR arm**

**
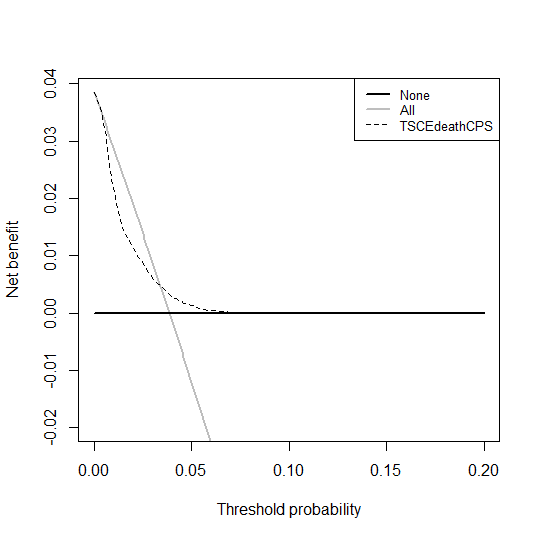
**
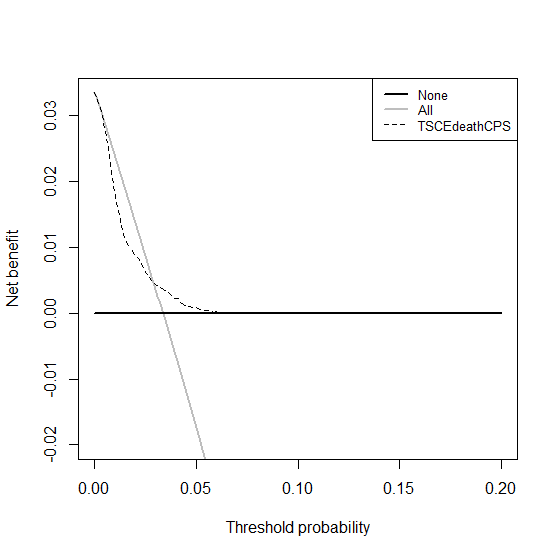


**PLCO CXR arm PLCO Control arm**

**
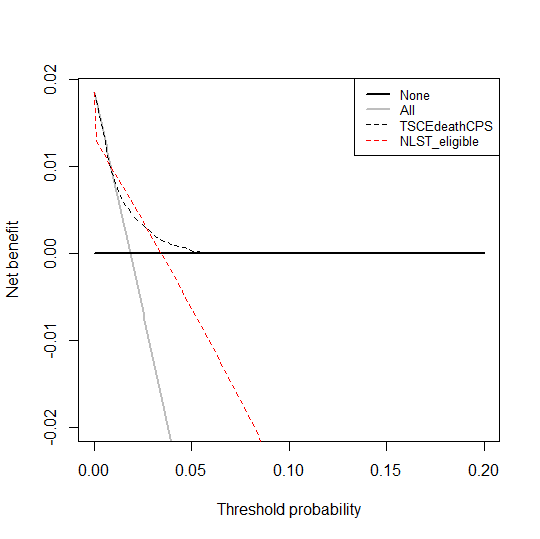
** **
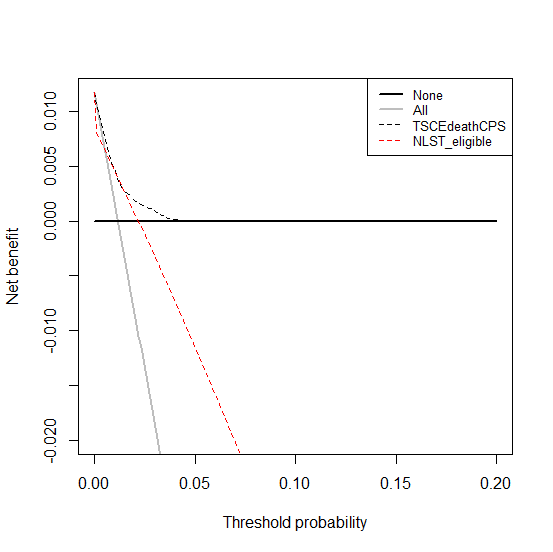
**

**Figure P: Decision curve analyses for the TSCE CPS lung cancer death model for 6-year lung cancer mortality in all datasets**

**NLST CT arm NLST CXR arm**

**
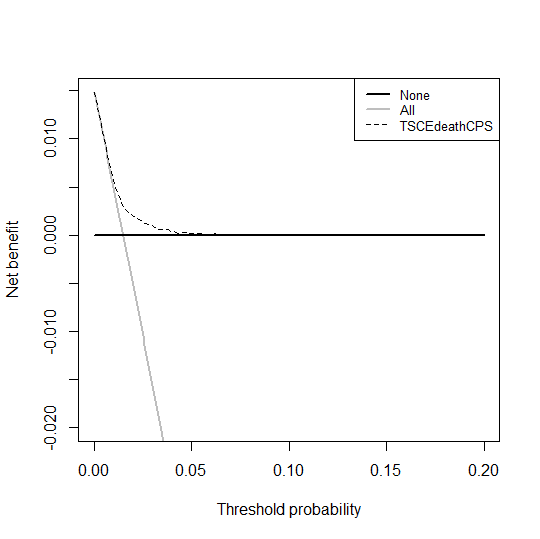
**
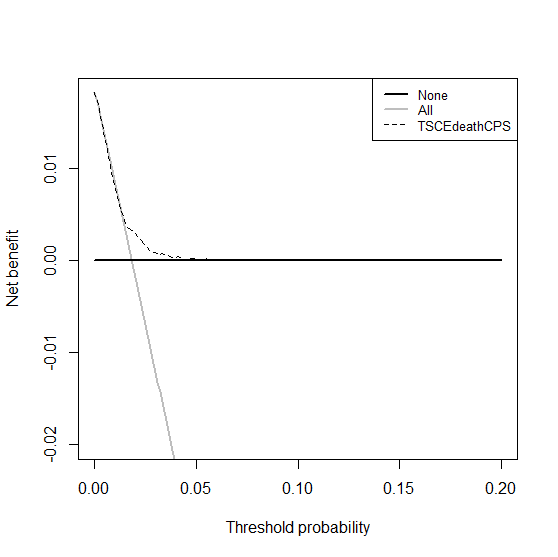


**PLCO CXR arm PLCO Control arm**

**
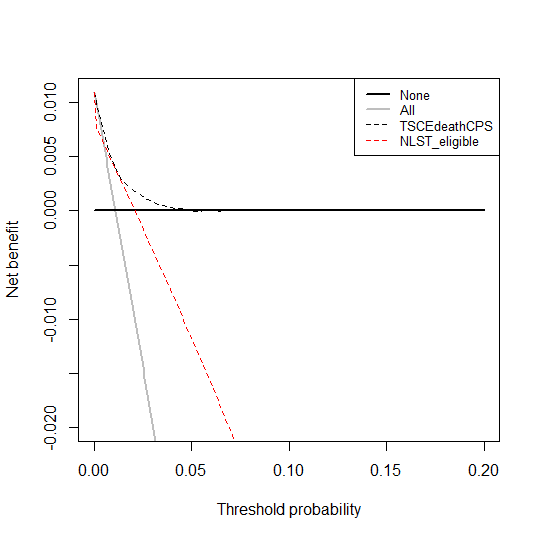
** **
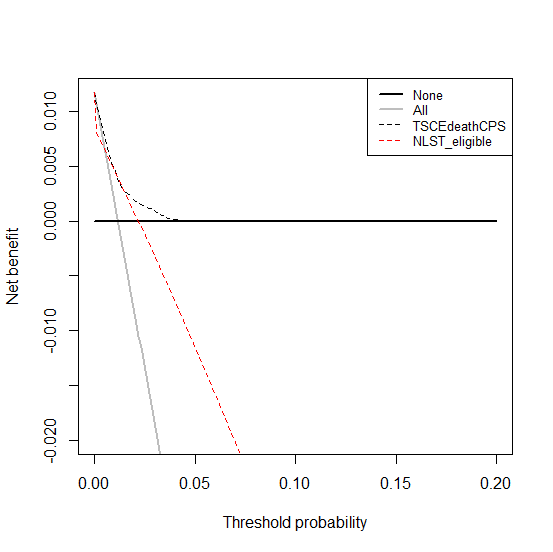
**

**Figure Q: Decision curve analyses for the TSCE NHS/HPFS lung cancer death model for 6-year lung cancer incidence in all datasets**

**NLST CT arm NLST CXR arm**

**
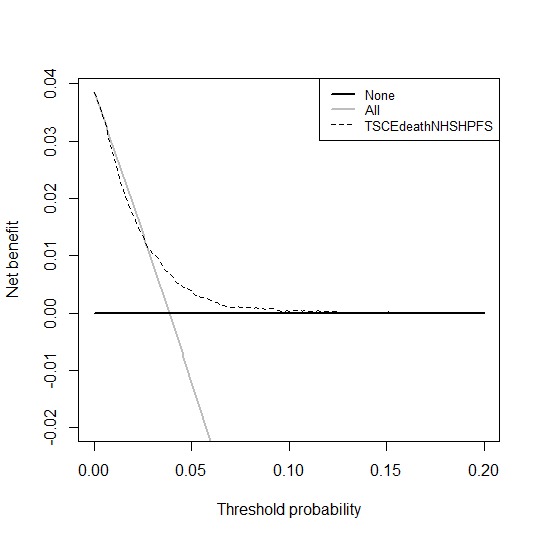
**
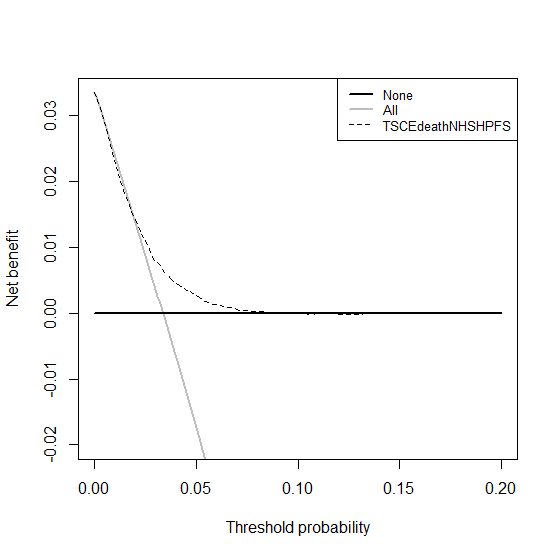


**PLCO CXR arm PLCO Control arm**

**
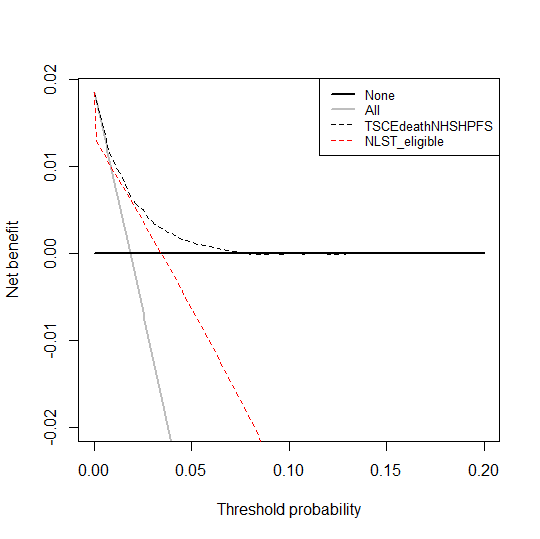

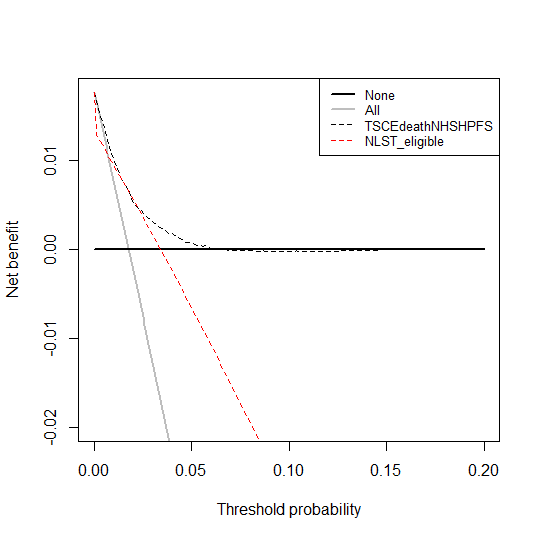
**

**Figure R: Decision curve analyses for the TSCE NHS/HPFS lung cancer death model for 6-year lung cancer mortality in all datasets**

**NLST CT arm NLST CXR arm**

**
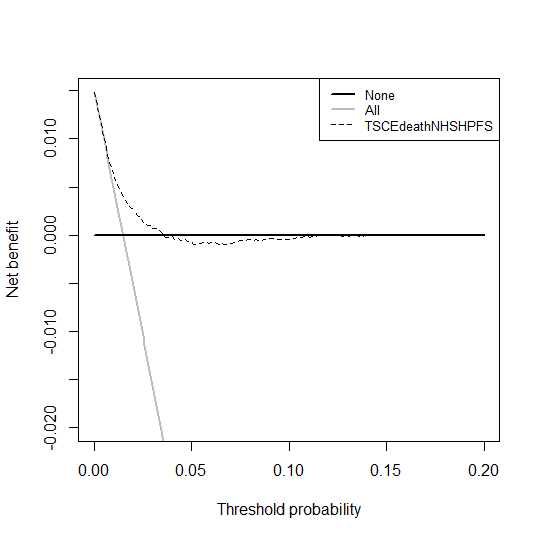
**
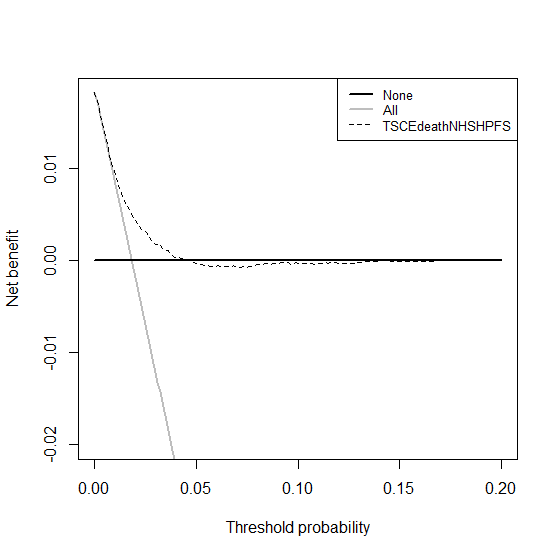


**PLCO CXR arm PLCO Control arm**

**
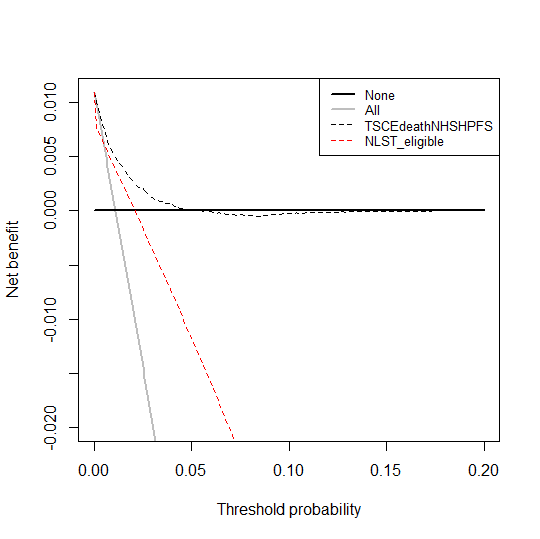

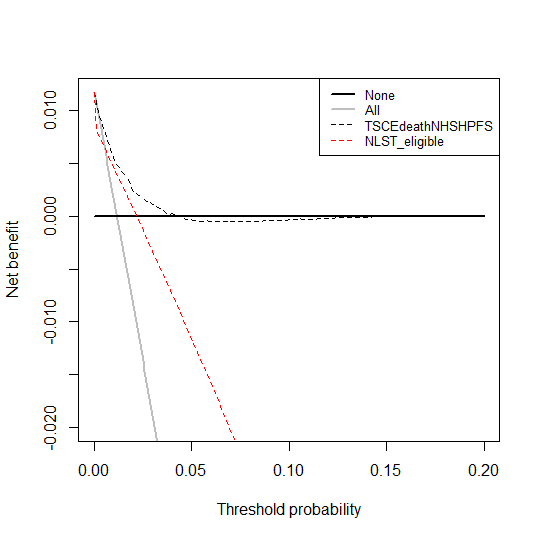
**

**Table A: Lower and upper risk thresholds for which the risk prediction models have a positive net-benefit compared to the National Lung Screening Trial (NLST) criteria for 6-year lung cancer death**

| **Model/dataset** | **NLST (computed tomography-arm)** | |  | **NLST (chest radiography arm)** | |  | **PLCO (chest radiography arm)** | |  | **PLCO (Control arm** | |
| --- | --- | --- | --- | --- | --- | --- | --- | --- | --- | --- | --- |
|  | Lower risk threshold (WF*) | Upper risk threshold (WF*) |  | Lower risk threshold (WF*) | Upper risk threshold (WF*) |  | Lower risk threshold (WF*) | Upper risk threshold (WF*) |  | Lower risk threshold (WF*) | Upper risk threshold (WF*) |
| Bach model | 0.2% (499.0) | 1.8% (54.6) |  | 0.9%  (110.1) | 2.9%  (33.5) |  | 0.1% (999.0) | 4.3% (22.3) |  | 0.2% (499.0) | 3.6% (26.8) |
| LLP model | 0.7% (141.9) | 2.3% (42.5) |  | 0.9%  (110.1) | 2.9%  (33.5) |  | 1.3% (75.9) | 3.3% (29.3) |  | 1.2% (82.3) | 2.9% (33.5) |
| Simplified LLP model | 0.7% (141.9) | 2.6% (37.5) |  | 0.9%  (110.1) | 3.0%  (32.3) |  | 0.3% (332.3) | 4.2% (22.8) |  | 0.1% (999.0) | 3.5% (27.6) |
| PLCOm2012 model | 0.3% (332.3) | 2.3% (42.5) |  | 0.1%  (999.0) | 3.5%  (27.6) |  | 0.1% (999.0) | 5.4% (17.5) |  | 0.1% (999.0) | 5.0% (19.0) |
| Simplified PLCOm2012 model | 0.5% (199.0) | 3.7% (26.0) |  | 0.7%  (141.9) | 4.5%  (21.2) |  | 0.1% (999.0) | 5.9% (16.0) |  | 0.2% (499.0) | 4.9% (19.4) |
| TSCE lung cancer incidence model | 0.2% (499.0) | 2.7% (36.0) |  | 0.1%  (999.0) | 3.9%  (24.6) |  | 0.2% (499.0) | 4.8% (19.8) |  | 0.2% (499.0) | 4.1% (23.4) |
| Knoke model | 0.5% (199.0) | 7.4% (12.5) |  | 0.7%  (141.9) | 7.6%  (12.2) |  | 0.4% (249.0) | 7.7% (12.0) |  | 1.3% (75.9) | 5.7% (16.5) |
| TSCE CPS lung cancer death model | 0.6% (165.7) | 7.1% (13.1) |  | 1.3%  (75.9) | 6.2%  (15.1) |  | 0.1% (999.0) | 5.0% (19.0) |  | 1.5% (65.7) | 4.2% (22.8) |
| TSCE NHS/HPFS lung cancer death model | 0.2% (499.0) | 3.5% (27.6) |  | 0.1%  (999.0) | 4.7%  (20.3) |  | 0.1% (999.0) | 5.5% (17.2) |  | 0.2% (499.0) | 4.1% (23.4) |

*Weighting factor corresponding to the risk threshold; i.e. the ratio of how much worse missing one case of lung cancer that could be detected through screening is valued compared to unnecessarily screening one person.

**Abbreviations:** National Lung Screening Trial (NLST); Prostate, Lung, Colorectal and Ovarian Cancer Screening Trial (PLCO), Liverpool Lung Project (LLP), Two-Stage Clonal Expansion (TSCE), Cancer Prevention Study (CPS), Nurses’ Health Study / Health Professionals Follow-up Study (NHS/HPFS).
