# Supplementary material for: Risk prediction models for selection of lung cancer screening candidates: A retrospective validation study
Source: PLoS Med. 2017 Apr 4;14(4):e1002277. doi: 10.1371/journal.pmed.1002277 (PMC5380315; doi:10.1371/journal.pmed.1002277)
Supplement: S7 Appendix — (DOCX) [file pmed.1002277.s007.docx]

**S7 Appendix: Comparison of the evaluated lung cancer risk prediction models at risk thresholds similar to the National Lung Screening Trial eligibility criteria**

**Figure A: Sensitivity, specificity and risk thresholds for the investigated risk models and the NLST eligiblity criteria for 6-year lung cancer mortality in the PLCO chest radiography arm**


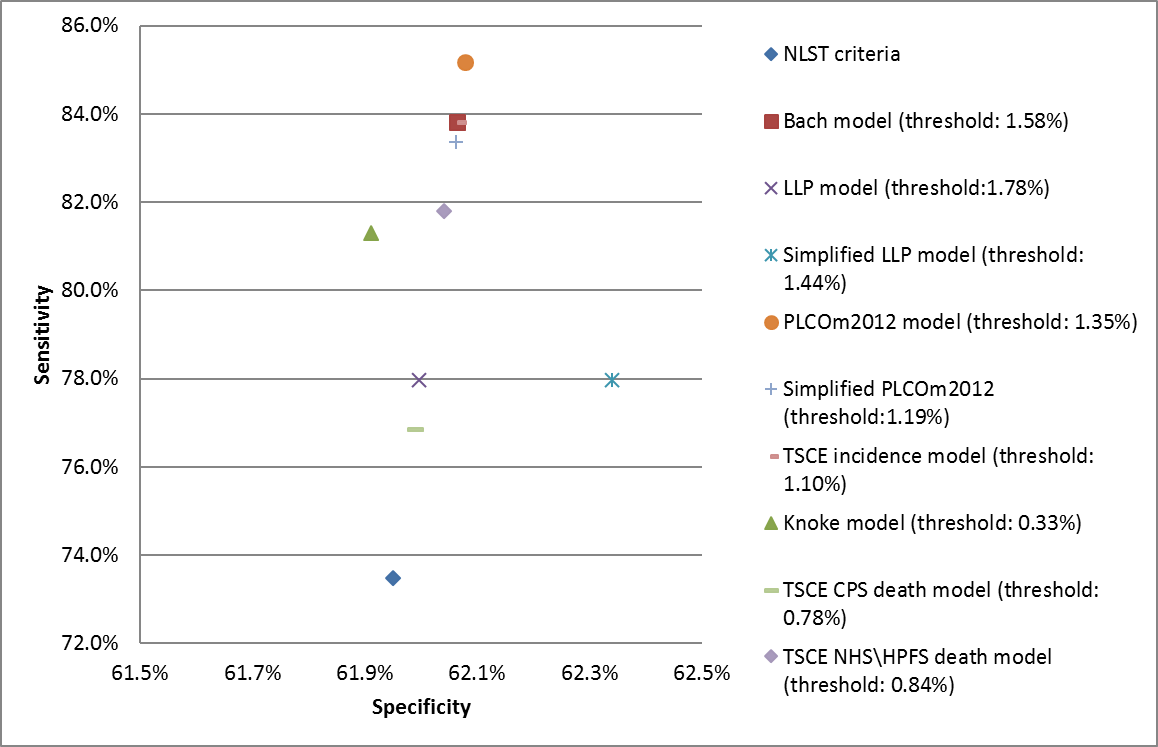


**Abbreviations:** National Lung Screening Trial (NLST); Prostate, Lung, Colorectal and Ovarian Cancer Screening Trial (PLCO).

|  | **NLST criteria** | **Bach model** | **LLP model** | **Simplified LLP model** | **PLCOm2012 model** | **Simplified PLCOm2012 model** | **TSCE incidence model** | **Knoke model** | **TSCE CPS death model** | **TSCE NHS/HPFS model** |
| --- | --- | --- | --- | --- | --- | --- | --- | --- | --- | --- |
| **Sensitivity (95% confidence interval)** | 73.5% (69.1-77.5%) | 83.8% (80.1-87.1%) | 78.0% (73.8-81.7%) | 78.0% (73.8-81.7%) | 85.2% (81.5-88.3%) | 83.4% (79.6-86.7%) | 83.8% (80.1-87.1%) | 81.3% (77.4-84.8%) | 76.9% (72.7-80.7%) | 81.8% (77.9-85.3%) |
| **Specificity (95% confidence interval)** | 61.9% (61.5-62.4%) | 62.1% (61.6-62.5%) | 62.0% (61.5-62.5%) | 62.3% (61.9-62.8%) | 62.1% (61.6-62.6%) | 62.1%  (61.6-62.5%) | 62.1%  (61.6-62.5%) | 61.7% (61.4-62.4%) | 62.0% (61.5.62.5%) | 62.0% (61.6-62.5%) |

**Figure B: Sensitivity, specificity and risk thresholds for the investigated risk models and the NLST eligiblity criteria for 6-year lung cancer incidence in the PLCO control arm**


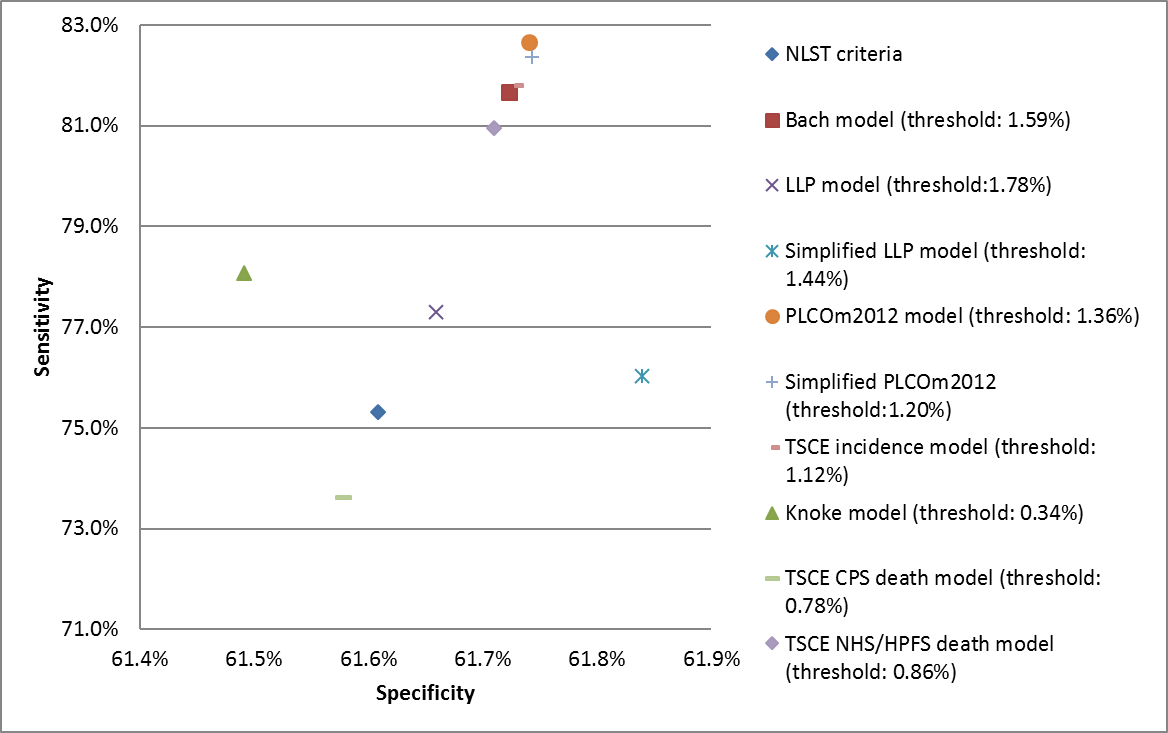


**Abbreviations:** National Lung Screening Trial (NLST); Prostate, Lung, Colorectal and Ovarian Cancer Screening Trial (PLCO).

|  | **NLST criteria** | **Bach model** | **LLP model** | **Simplified LLP model** | **PLCOm2012 model** | **Simplified PLCOm2012 model** | **TSCE incidence model** | **Knoke model** | **TSCE CPS death model** | **TSCE NHS/HPFS model** |
| --- | --- | --- | --- | --- | --- | --- | --- | --- | --- | --- |
| **Sensitivity (95% confidence interval)** | 75.3%  (72.0-78.5%) | 81.7% (78.6-84.4%) | 77.3%  (74.0-80.3%) | 76.0%  (72.7-79.1%) | 82.7%  (79.7-85.4%) | 82.4%  (79.4-85.1%) | 81.8%  (78.8-84.6%) | 78.1%  (74.8-81.1%) | 73.6%  (70.2-76.8%) | 81.0%  (77.9-83.8%) |
| **Specificity (95% confidence interval)** | 61.6%  (61.1-62.1%) | 61.7%  (61.2-62.2%) | 61.7%  (61.2-62.1%) | 61.8%  (61.4-62.3%) | 61.7%  (61.3-62.2%) | 61.7%  (61.3-62.2%) | 61.7%  (61.2-62.2%) | 61.5%  (61.0-62.0%) | 61.6%  (61.1-62.1%) | 61.7%  (61.2-62.2%) |

**Figure C: Sensitivity, specificity and risk thresholds for the investigated risk models and the NLST eligiblity criteria for 6-year lung cancer mortality in the PLCO control arm**


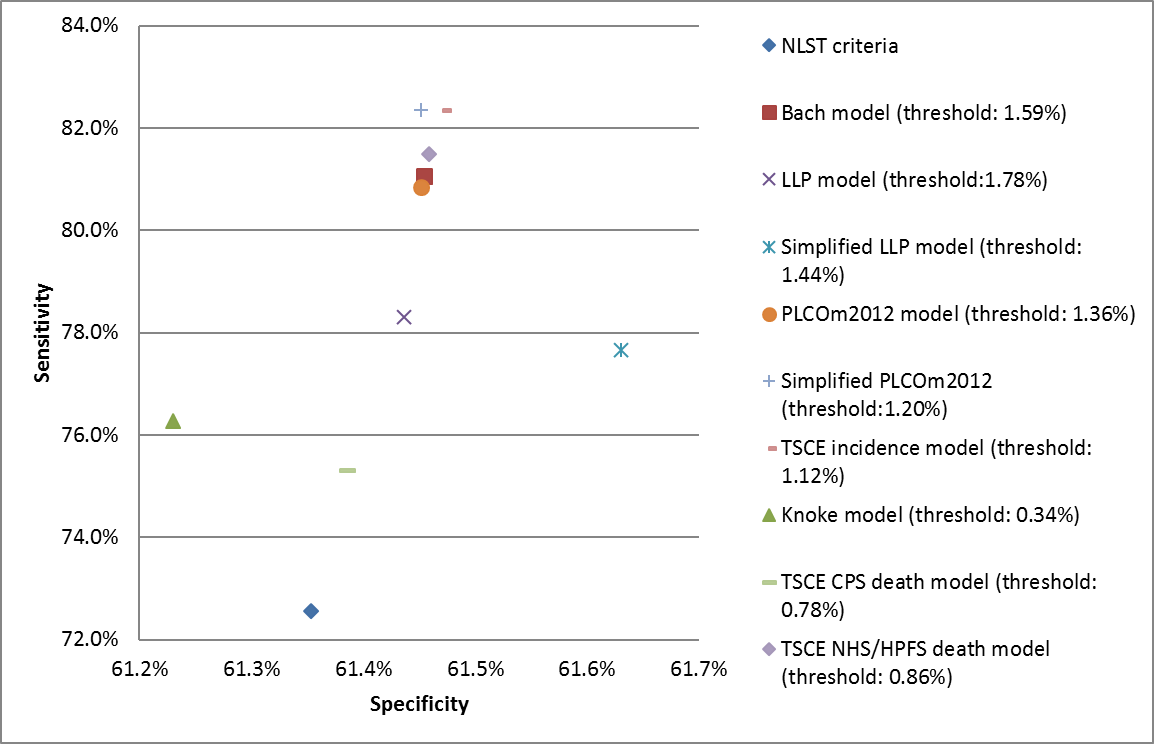


|  | **NLST criteria** | **Bach model** | **LLP model** | **Simplified LLP model** | **PLCOm2012 model** | **Simplified PLCOm2012 model** | **TSCE incidence model** | **Knoke model** | **TSCE CPS death model** | **TSCE NHS/HPFS model** |
| --- | --- | --- | --- | --- | --- | --- | --- | --- | --- | --- |
| **Sensitivity (95% confidence interval)** | 72.6%  (68.3-76.5%) | 81.1% (77.2-84.5%) | 78.3% (74.3-81.9%) | 77.7% (73.6-81.3%) | 80.9% (77.0-84.3% | 82.3% (78.6-85.7%) | 82.3% (78.6-85.7%) | 76.3% (72.2-80.1%) | 75.3% (71.2-79.2%) | 81.5% (77.7-84.9%) |
| **Specificity (95% confidence interval)** | 61.4% (60.9-61.8%) | 61.5% (61.0-61.9%) | 61.4% (61.0-61.9%) | 61.6% (61.1-62.1%) | 61.5% (61.0-61.9%) | 61.5% (61.0-62.0%) | 61.5% (61.0-62.0%) | 61.2% (60.7-61.7%) | 61.4% (60.9-61.9%) | 61.5% (61.0-61.9%) |

**Abbreviations:** National Lung Screening Trial (NLST); Prostate, Lung, Colorectal and Ovarian Cancer Screening Trial (PLCO).
