# Supplementary material for: Risk prediction models for selection of lung cancer screening candidates: A retrospective validation study
Source: PLoS Med. 2017 Apr 4;14(4):e1002277. doi: 10.1371/journal.pmed.1002277 (PMC5380315; doi:10.1371/journal.pmed.1002277)
Supplement: S8 Appendix — (DOCX) [file pmed.1002277.s008.docx]

| **Section/Topic** | **Item** | **Checklist Item** | **Page** |
| --- | --- | --- | --- |
| **Title and abstract** | | | |
| Title | 1 | Identify the study as developing and/or validating a multivariable prediction model, the target population, and the outcome to be predicted. | Title |
| Abstract | 2 | Provide a summary of objectives, study design, setting, participants, sample size, predictors, outcome, statistical analysis, results, and conclusions. | Abstract |
| **Introduction** | | | |
| Background and objectives | 3a | Explain the medical context (including whether diagnostic or prognostic) and rationale for developing or validating the multivariable prediction model, including references to existing models. | Introduction paragraphs 2 and 3 |
|  | 3b | Specify the objectives, including whether the study describes the development or validation of the model or both. | Introduction paragraph 3 |
| **Methods** | | | |
| Source of data | 4a | Describe the study design or source of data (e.g., randomized trial, cohort, or registry data), separately for the development and validation data sets, if applicable. | Materials and Methods section “Study population”, References 1, 22-24 |
|  | 4b | Specify the key study dates, including start of accrual; end of accrual; and, if applicable, end of follow-up. | Materials and Methods section “Study population”, References 1, 22-24 |
| Participants | 5a | Specify key elements of the study setting (e.g., primary care, secondary care, general population) including number and location of centres. | Materials and Methods section “Study population”, References 1, 22-24 |
|  | 5b | Describe eligibility criteria for participants. | Materials and Methods section “Study population”, References 1, 22-24 |
|  | 5c | Give details of treatments received, if relevant. | Materials and Methods section “Study population”, References 1, 22-24 |
| Outcome | 6a | Clearly define the outcome that is predicted by the prediction model, including how and when assessed. | Materials and Methods section “Lung cancer risk prediction models” paragraphs 1-2, Table 1 and S1 Appendix. |
|  | 6b | Report any actions to blind assessment of the outcome to be predicted. | Materials and Methods section “Study population”, References 1, 22-24 |
| Predictors | 7a | Clearly define all predictors used in developing or validating the multivariable prediction model, including how and when they were measured. | Materials and Methods sections “Study population”, “Lung cancer risk prediction models”, Tables 1-2 and S1 Appendix |
|  | 7b | Report any actions to blind assessment of predictors for the outcome and other predictors. | Materials and Methods section “Study population” paragraphs 1 and 2. References 1, 22-24 |
| Sample size | 8 | Explain how the study size was arrived at. | Materials and Methods section “Study population” paragraphs 1 and 2. References 1, 22-24 |
| Missing data | 9 | Describe how missing data were handled (e.g., complete-case analysis, single imputation, multiple imputation) with details of any imputation method. | Materials and Methods sections “Lung cancer risk prediction models” paragraphs 3-5, and “Multiple imputation of missing values” |
| Statistical analysis methods | 10a | For validation, describe how the predictions were calculated. | S1 Appendix |
|  | 10b | Specify all measures used to assess model performance and, if relevant, to compare multiple models. | Materials and Methods sections: “Statistical analyses”, “Aspects of calibration performance”, “Discrimination” and “Clinical usefulness” |
|  | 10c | Describe any model updating (e.g., recalibration) arising from the validation, if done. | Not applicable |
| Risk groups | 11 | Provide details on how risk groups were created, if done. | Materials and Methods section “Clinical usefulness”, paragraph 7 |
| Development vs. validation | 12 | For validation, identify any differences from the development data in setting, eligibility criteria, outcome, and predictors. | Materials and Methods sections “Study population”, “Lung cancer risk prediction models”, Tables 1-2, Results sections “Characteristics of study populations” and “Differences in levels of absolute risk”, Figure 1, S1 Appendix |
| **Results** | | | |
| Participants | 13a | Describe the flow of participants through the study, including the number of participants with and without the outcome and, if applicable, a summary of the follow-up time. A diagram may be helpful. | Table 2 and S2 Appendix |
|  | 13b | Describe the characteristics of the participants (basic demographics, clinical features, available predictors), including the number of participants with missing data for predictors and outcome. | Table 2, S2 Appendix and S3 Appendix |
|  | 13c | For validation, show a comparison with the development data of the distribution of important variables (demographics, predictors and outcome). | Table 2, S1 Appendix and S2 Appendix |
| Model performance | 16 | Report performance measures (with CIs) for the prediction model. | Results sections “Aspects of calibration performance”, “Discrimination”, “Clinical Usefulness” and “Comparison to NLST eligibility criteria”, Figures 2-3, Tables 3-4, S4 Appendix, S5 Appendix, S6 Appendix, S7 Appendix |
| Model-updating | 17 | If done, report the results from any model updating (i.e., model specification, model performance). | Not applicable |
| **Discussion** | | | |
| Limitations | 18 | Discuss any limitations of the study (such as nonrepresentative sample, few events per predictor, missing data). | Discussion paragraphs 5-7 |
| Interpretation | 19a | For validation, discuss the results with reference to performance in the development data, and any other validation data. | Discussion paragraphs 1-4 and S1 Appendix |
|  | 19b | Give an overall interpretation of the results, considering objectives, limitations, results from similar studies, and other relevant evidence. | Discussion section |
| Implications | 20 | Discuss the potential clinical use of the model and implications for future research. | Discussion paragraphs 1-2 and 9-10 |
| **Other information** | | | |
| Supplementary information | 21 | Provide information about the availability of supplementary resources, such as study protocol, Web calculator, and data sets. | S1 Appendix through S8 Appendix |
| Funding | 22 | Give the source of funding and the role of the funders for the present study. | Acknowledgements paragraphs 1-2 and Funding statement |
